# Nanotechnology in Glycomics: Applications in Diagnostics, Therapy, Imaging, and Separation Processes

**DOI:** 10.1002/med.21420

**Published:** 2016-11-15

**Authors:** Erika Dosekova, Jaroslav Filip, Tomas Bertok, Peter Both, Peter Kasak, Jan Tkac

**Affiliations:** ^1^ Department of Glycobiotechnology, Institute of Chemistry Slovak Academy of Sciences Dubravska cesta 9 845 38 Bratislava Slovakia; ^2^ Center for Advanced Materials Qatar University P.O. Box 2713 Doha Qatar; ^3^ School of Chemistry, Manchester Institute of Biotechnology The University of Manchester 131 Princess Street Manchester M1 7DN UK

**Keywords:** nanotechnology, nanomaterials, glycomics, glycan sensing, glycan enrichment and active glycoprofiling, glycan‐based vaccines/therapeutics, cell tissue targeting/imaging and drug delivery

## Abstract

This review comprehensively covers the most recent achievements (from 2013) in the successful integration of nanomaterials in the field of glycomics. The first part of the paper addresses the beneficial properties of nanomaterials for the construction of biosensors, bioanalytical devices, and protocols for the detection of various analytes, including viruses and whole cells, together with their key characteristics. The second part of the review focuses on the application of nanomaterials integrated with glycans for various biomedical applications, that is, vaccines against viral and bacterial infections and cancer cells, as therapeutic agents, for in vivo imaging and nuclear magnetic resonance imaging, and for selective drug delivery. The final part of the review describes various ways in which glycan enrichment can be effectively done using nanomaterials, molecularly imprinted polymers with polymer thickness controlled at the nanoscale, with a subsequent analysis of glycans by mass spectrometry. A short section describing an active glycoprofiling by microengines (microrockets) is covered as well.

## 1. NANOTECHNOLOGY

**Figure 1:**
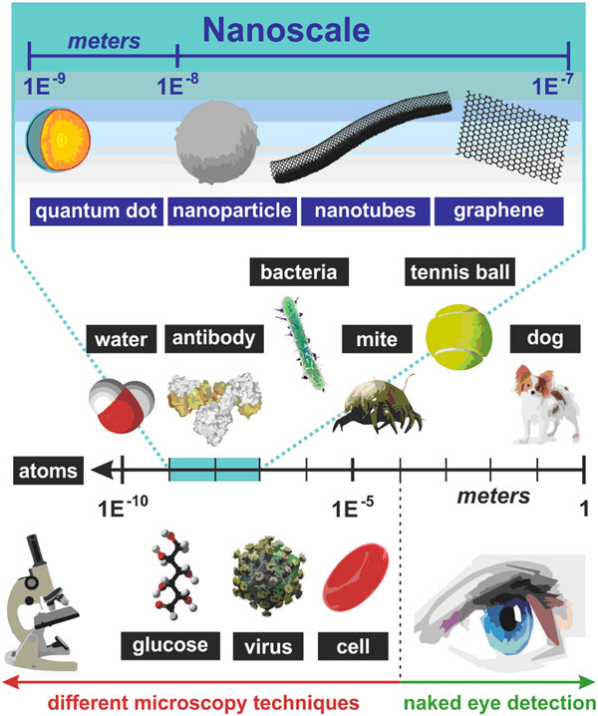
Various forms of nanomaterials, which can be integrated with biomolecules for applications in glycomics with their size related to other objects of our world, micro‐ and nano‐world.

The term nanotechnology was first used in 1974 by Professor Nario Taniguchi,^1^ but the idea and concepts behind nanoscience began more than a decade earlier with a talk by Professor Richard Feynman, “There´s Plenty of Room at the Bottom.”^2^ In this talk, the process by which science could control and manipulate single atoms was described. The real breakthrough in nanoscience came in 1981,^3^ when the scanning tunneling microscope was developed, enabling the observation of individual atoms, and after the invention of atomic force microscopy (AFM), nanotechnology as a scientific discipline was born. Currently, nanotechnology covers processes for the design, preparation, and application of extremely small things. Materials and structures could be designated as “nano” only if their size (at least one dimension) is within the range of 1 to 100 nm.^4^ The discovery of nanomaterials, such as fullerenes^5^ and graphene,^6^ awarded the Nobel Prize to theirs discoverers. Nanomaterials now form a large family of materials, including metal/semiconducting nanoparticles (NPs), quantum dots (QDs), nanowires, fullerenes, graphene and its derivatives, graphene QDs, and carbon nanotubes (CNTs, Fig. 1).^7^, ^8^ It is not only the size of nanomaterials that matters, but their remarkable physical and chemical properties are gaining increasing attention from scientists both from fundamental and application points of view, using nanomaterials in biology, chemistry, and applied physics.^9^^–^ 14 An interesting feature of nanomaterials is that their dimension is similar to that of biomolecules, such as DNA/RNA, proteins, lipids, and carbohydrates, with numerous applications in biology and biomedicine as well.^9^^–^ 14

## 2. GLYCOMICS

**Figure 2:**
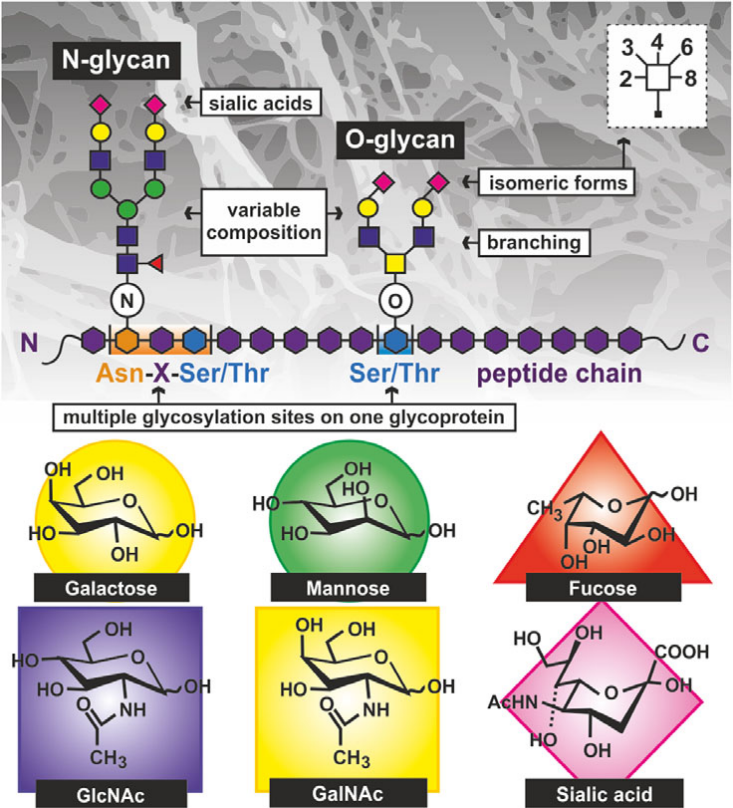
The challenges in glycan analysis and separation due to complex and heterogeneous structure of glycans as a result of a variable monosaccharide composition, branching, and multiple glycosylation sites of glycoconjugates. Structure of the most common carbohydrates moieties within glycans is shown as well.

For quite a long time, carbohydrates were considered only as reservoirs of energy and as building blocks providing the organisms strength, that is, cellulose and chitin, which are the most abundant polymers on Earth.^15^ Glycans are complex carbohydrates consisting of saccharide units that link together and are attached to proteins and lipids to form glycoproteins and glycolipids, respectively (Fig. 2).^16^ Glycans are bound to proteins during co‐ and posttranslational modifications via a multistep enzymatic process in the endoplasmic reticulum and Golgi apparatus.^17^ Glycans can be classified into several categories based on the bond between a glycan and a protein: *N*‐glycans (via –NH_2_ group to asparagine), *O*‐glycans (via –OH group to serine, threonine, or hydroxylated amino acids), and less‐abundant forms of glycans, such as *C*‐glycans (C—C bond via tryptophan) and the quite unusual *S*‐glycans (C—S bond via cysteine).^18^, ^19^ Moreover, glycans can be branched by the formation of biantennary, triantennary, and more complex antennary structures.^20^, ^21^

Glycomics as a scientific discipline studying the structure and function of glycans is a younger sister of more developed genomics and proteomics. There are several reasons why glycomics is still behind genomics and proteomics: (i) glycans and glycoconjugates are more structurally complex than proteins and DNA/RNA; (ii) it is quite challenging to determine glycan identity/sequences using traditional instrumental techniques; and (iii) glycan biosynthesis cannot be predicted from a template as in the case of DNA and proteins. In addition, the chemical synthesis of oligosaccharides is very challenging (protection of all functional groups with a variety of protecting groups in order to generate the site‐specific deprotection of those which are intended to form a chemical bond) and represents yet another obstacle to obtain valuable intermediate or incomplete structures. Despite intensive research in genomics and proteomics, there are still many questions that cannot be answered by analyzing genome and proteome alone, and glycomics has to be added into the equation. Currently, it is estimated that 70% of human cytosolic proteins and 80% of membrane proteins are glycosylated, underlining the important involvement of glycans within the human body.^16^

The function of glycans in living organisms has been revealed at an amazing pace with involvement of glycans in cell—cell and host—pathogen interactions, the binding of cells to the extracellular matrix, the immune response, the differentiation of cells, and other physiological and pathological processes.^22^, ^23^, ^24^, ^25^, ^26^, ^27^, ^28^, ^29^, ^30^, ^31^, ^32^, ^33^ Since glycosylation involves numerous proteins during synthesis, including transport proteins, glycosyltransferases/glycosidases, and other glycan‐processing enzymes, depending on the actual physiological state of the cell, distinct glycoforms of the same protein within a particular cell can be formed.^34^ Each form of such protein can possess different properties, such as distribution in the cell, folding, or stability; thus, glycan alterations can influence the physiological functions of proteins and can indicate pathological processes as well.^35^ The correlation between diverse forms of cancer and altered glycan structures has been intensively studied. An increasing amount of evidence suggests that the occurrence of a certain glycan structure correlates with cancer invasiveness, the presence of tumor circulating cells, and the ability to metastasize distant organs.^35^, ^36^, ^37^, ^38^, ^39^ Thus, glycoproteins can be used as cancer biomarkers for screening, diagnostic, predictive, and monitoring purposes.^40^ Changes in protein glycosylation have also been observed in other pathological processes, such as inflammatory and autoimmune diseases, and in processes of aging.^41^, ^42^ Changes in the glycosylation of immunoglobulin G (IgG) are influenced by pregnancy and gender,^43^ which should to be considered in future studies.

Several methods have been developed for structural glycan analysis. The most common method is mass spectrometry (MS) in combination with other separation techniques, including a battery of electrophoretic and chromatographic methods and nuclear magnetic resonance. Instrumental techniques can be effectively applied for glycan analysis, but the identification of glycan isoforms and the linkages between carbohydrates within a glycan structure are not trivial issues.^44^ Moreover, such an analysis requires sophisticated instrumentation and skilled operators to correctly interpret data with low analysis throughput. Moreover, various pretreatment steps (glycan release, enrichment, and modification) are needed prior to analysis.

Lectins are carbohydrate‐binding proteins other than enzymes or antibodies and other than carbohydrate sensor/transport proteins^45^ with potential therapeutic applications.^46^ Lectins as natural glycan‐recognizing proteins can be effectively applied to analyze glycans because they can detect intact glycans still attached to proteins or even cells.^47^, ^48^, ^49^, ^50^ The first step toward the effective utilization of lectins in glycan analysis and for diagnostic purposes was the introduction of lectin microarrays/biochips.^51^, ^52^, ^53^, ^54^ Glycan microarrays are a valuable tool in glycomics for the identification of glycan‐binding proteins,^55^, ^56^ and together with lectin microarrays, such highly parallel analyses with a minute consumption of reagents had led to numerous important discoveries regarding glycan involvement in various cellular processes. However, the main disadvantages of microarray‐based analysis are the low sensitivity of detection with a rather high limit of detection (LOD) and the need to fluorescently label either the ligand or sample.^51^, ^52^ This is why alternatives to glycan/lectin microarrays with a biosensor detection platform as a viable option have been intensively sought.^57^, ^58^, ^59^

Because of the above‐mentioned importance of glycans in pathological processes, there is substantial interest in visualizing them directly within intact cells and tissues. Numerous strategies have been developed to achieve this, using glycan interactions to allow for a targeted delivery of an imaging probe to a chosen cell/tissue; hence, a precise and accurate detection of, for example, tumors or other cells/tissues with specific receptors was possible. Moreover, when a therapeutic agent is selectively delivered to a targeted cell/tissue, a significant reduction in adverse side effects can be achieved. Chemotherapy is good example of this approach—cytotoxic agents are delivered to and released only in tumor tissues, while healthy cells are not affected. Recently, a theranostic approach has been frequently used, relying on particles selectively delivering both an imaging probe and a drug to a targeted cell/tissue. Furthermore, the specificity of these interactions can be used to design vaccines and novel therapeutic approaches, as glycans bound to receptors that are naturally targeted by pathogens prevent the first step of infection (e.g., pathogen binding). Finally, a partial functional restoration of glycosidases used to cure “liposomal storage diseases” (e.g., Gaucher disease) mediated via glycan interactions has been described as well.

## 3. NANOTECHNOLOGY IN GLYCOMICS

**Scheme 1:**
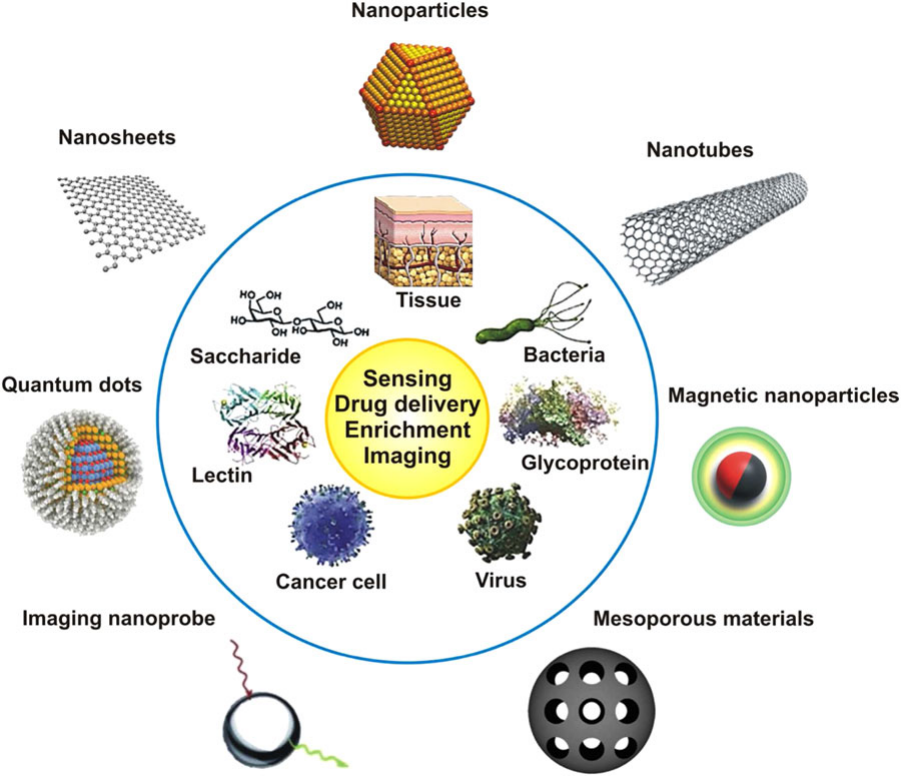
Overview of different nanomaterials (outside the blue circle) used in combination with biomaterials/biomolecules (inside the blue circle) for various applications (in yellow circle) reviewed in this study. The figure was redrawn from 7. Copyright (2014), with permission from Elsevier.

One of the earliest efforts to describe the beneficial properties of nanomaterials in the field of glycomics was published by Reichardt et al. in 2013, applying nanomaterials as support for the delivery of vaccines, cellular imaging, biosensors/diagnostics, glycan isolation, and enrichment.^9^ Since this review was published, there has been an explosion of papers focusing on the integration of nanomaterials in the field of glycomics (Scheme 1), and in this article, we would like to provide a summary of the achievements in this field in the last 3 years (from 2013).

In the sections describing the integration of nanomaterials within various types of transducers, you will find a comprehensive review of recently developed biosensors, bioanalytical devices, and protocols for the detection of various analytes, including viruses and whole cells, together with their main characteristics (Section 4.). It is divided into subsections according to nanomaterials used, that is, metal NPs (Section 4.A), hybrid nanomaterials (Section 4.B), carbon nanomaterials (Section 4.C), QDs (Section 4.G), and others—mostly glycopolymers (Section 4.D) and synthetic receptors (Section 4.E), used as an immobilization platform or as a part of an amplification strategy. Some of these applications have been summarized in recent reviews.^7^, ^60^

Sections describing the application of nanomaterials for various biomedical applications will basically cover the progress in the fabrication of glycan‐based NPs that can either induce an immune response (vaccines against viral and bacterial infections and, more recently, even against cancer cells) or possess another therapeutic effect, including antibacterial effect or enzyme inhibition (Section 5.). An extensive section covering glycan‐targeted nanocarriers for drug delivery (Section 6.A); therapeutic purposes prepared from carbonaceous, metallic, or polymeric nanomaterials (Section 6.B); and advances in in vivo imaging (Section 6.C), that is, the selective delivery of imaging probes, is provided as well. There are also sections and subsections dealing with the application of carbon, metal, polymer, and magnetic NPs (MNPs) for nuclear magnetic resonance imaging (NMRi) and another subsection focused on other imaging methods besides fluorescence and NMRi (Section 6.C). There are only two review papers either not comprehensively covering the application of a diverse range of NPs—covering biomedical applications only of gold‐ and polymer‐based glyconanoparticles^61^—or not focused on recent developments in this field.^62^

The final part of the paper will describe various ways for glycan release (Section 7.A) and enrichment (Section 7.C), which can occur in a timely way with a minute consumption of precious samples using various nanomaterials (Sections 7.D–7.K). Glycan‐enrichment procedures performed directly on matrix‐assisted laser desorption/ionization (MALDI) plates (Section 8.) will be provided as well. The application of nanomaterials or nanoporous materials for matrix‐free MS (Section 8.) will also be described. This section will also discuss the preparation of molecularly imprinted polymers (MIP) with polymer thickness controlled at the nanoscale for the selective affinity‐like enrichment of specific glycoproteins (Section 7.E). Moreover, active glycoprofiling by microengines/microrockets (Section 9.) will be covered as well. Only one review paper has focused on the application of nanotechnology in glycan enrichment; it was published in 2014 and only covers advancements in the field until the end of 2013.^63^

## 4. NANOGLYCOSENSING

Glycan moieties attached to protein backbones or lipids are destined mostly for the cell surface. Since glycosylation is the most common co‐ and posttranslational modification of proteins, it is also increasingly recognized as a phenotype modulator of various pathological changes on cell surfaces, mostly during cancer development,^35^, ^64^ and glycomics is thus believed to soon provide relevant information and detection strategies for glycan‐based diagnostics.^65^ A relatively small amount of glycans within a sample of interest could be present (e.g., a prostate‐specific antigen [PSA] contains only one glycan per molecule).^66^ Thus, for the structural analysis and identification of glycans using mainly MS, capillary electrophoresis, and liquid chromatography,^67^ enrichment methods must be performed prior to analysis.^63^ For the analysis alone, the use of glycosylated nanomaterials has gained increasing attention in recent years because NPs decorated with suitable biorecognition ligands, that is, glycan structures in a multivalent form similar to the glycocalyx structures on cell surfaces,^68^ could be used for the detailed study of glycan—lectin (Latin: *legere*—to choose, to read) interactions.^69^ The following section describes in detail the most recent progress in the area of glycosensing using various nanostructures, including (but not limited to) metal, carbon, and polymer nanomaterials with respect to the analytical performance of such devices for the analysis of glycoproteins, lectins, viruses, and bacteria or for dynamic *N*‐glycan evaluation on cell surfaces.

### A. Metal Nanomaterials

Different lectins, which are ubiquitously found in nature, often provide a model system for studying glycan—protein interactions. These interactions play a pivotal role in viral infections, cell adhesion, and the differentiation and progression of various diseases and are thus of great interest for many clinical applications. Despite this fact, they have attracted much less attention than antibodies.^70^ The most commonly used NPs are gold NPs (AuNPs) because they can be easily prepared and modified using standard thiol chemistry (a covalent‐like modification).^4^, ^71^ The synthesis, modification, and engineering of different (glyco)NPs, including metal, magnetic, and carbon NPs and QDs with a focus on biosensing applications, have been reviewed elsewhere.^60^, ^72^, ^73^

#### 1. Optical Biosensors

Moreover, using precisely engineered inter‐NP spacers (e.g., polymer grafts), it was possible to control the degree of plasmonic coupling between NPs.^74^ This principle was demonstrated with poly[(lactose)_m_‐*b*‐(pyridine)_n_] and bivalent galactose‐binding lectin from *Ricinus communis* (RCA_120_, *M*_w_ = 120 kDa) on gold nanorods (AuNRs), where the authors claimed to develop a protein assay with an LOD for lectin down to pictogram per milliliter (fM range).^74^

Other study^75^ focused on the preparation of a sandwich configuration with graphene oxide (GO) and phenoxy‐derivatized dextran (conjugated together through π—π stacking interactions) deposited on gold substrate for specific Concanavalin A (Con A) binding with a final amplification of the signal using dextran‐capped AuNPs. This system was, however, less sensitive than a previous one, offering an LOD of 0.39 μg mL^−1^ (3.75 nM), but still with a 28.7‐fold increase in sensitivity compared to a direct assay (without any amplification reagent).^75^ The application of different graphene‐based interfaces for the surface plasmon resonance (SPR) analysis of biospecific interactions between different ligand—receptor couples was recently reviewed.^76^

Since the change in the localized SPR (LSPR) signal of metal NPs is not very sensitive and provides only a little information about the nature of interacting molecules, Craig et al. aimed to develop an on/off surface‐enhanced Raman scattering (SERS) aggregation system for picomolar Con A detection. The authors took advantage of the multivalent character of the interaction (since Con A is a homotetrameric molecule at a physiological pH with four glycan‐binding sites) with aggregated NPs producing a strong electromagnetic field between their interfaces, resulting in an increased SERS intensity.^77^ The LOD for this system was as low as 40 pM for the chosen silver NPs (AgNPs) because they provided a tenfold increase in scattering efficiency compared to AuNPs.^77^ Plasmonic and, in general, label‐free methods are of a particular interest for glycan‐lectin‐based biosensing because of a negative effect of lectin/glycan labeling on the biorecognition. In 2015, a novel approach called plasmon waveguide resonance was introduced based on glass prisms coated with 50 nm silver and 460 nm silica layers derivatized with mannose and lactose using Cu^I^‐catalyzed Huisgen azide‐alkyne cycloaddition (CuAAC).^78^ In addition to “click chemistry” based techniques, photocoupling reactions with underivatized glycans represent an alternative approach comparable to CuAAC.^79^

AuNPs can be easily covalently attached on a plane gold surface via thiol linkers containing an –SH or –NH_2_ head group for sensitive glycoprotein detection using immobilized lectin molecules,^80^ providing an increased surface area for immobilization, as well as for the suppression of steric hindrance between lectin molecules because of the spherical shape of AuNPs. This effect proved to be so crucial that prepared impedimetric, a *Sambucus nigra* agglutinin (SNA) based biosensor, was more sensitive, with an LOD 3 orders of magnitude lower (shift from fM to aM range for the detection of sialylated glycoprotein fetuin) even though the total amount of lectin on the surface was lower in the NP‐based 3D configuration compared to the 2D (planar gold) configuration.^81^ Most often, metal NPs are used as signal amplifiers. NPs decorated with saccharide structures (d‐maltoheptaose as the largest carbon source among maltodextrins that can be transported to *Escherichia coli* cytoplasm) promote particle internalization for silica, magnetic, silica‐coated MNPs and silica‐coated QDs.^82^

Other optical detection platforms are based on colorimetric bioassays. The possibilities for the improvement of the selectivity and sensitivity of such assays for the detection of lectins, toxins, and viruses were recently reviewed.^83^ Besides lectins, different toxins possess the ability to specifically bind various saccharide residues. Within the so‐called AB_5_ bacterial toxin family (which also includes cholera toxin, shiga and shiga‐like toxins, and pertussis toxin), a protein is produced by enterotoxic *E. coli* causing “traveller´s diarrhoea”: heat‐labile enterotoxin. Poonthiyil et al. prepared an efficient colorimetric sensor using 12 nm AuNPs with attached thiol‐modified galactose moieties binding to the B‐subunit of heat‐labile enterotoxin (while the A‐subunit is typically the one causing a particular disease among all members of this family) that is able to detect the toxin down to 100 nM.^84^

**Figure 3:**
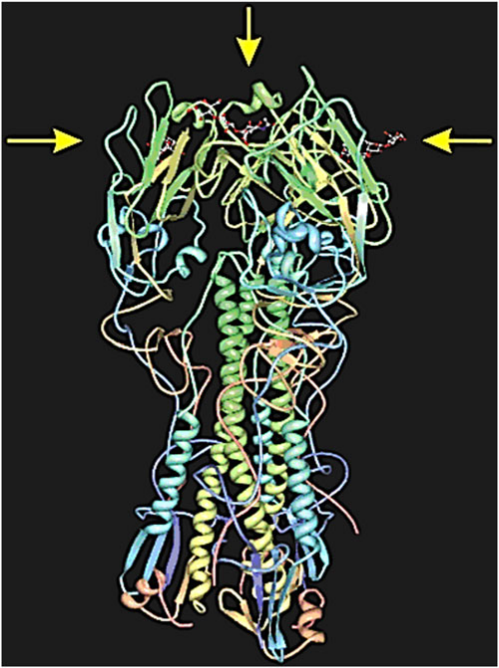
A hemagglutinin from H1N1 *Influenza* A virus (complexed to three molecules of 3´‐sialyllactose, denoted with yellow arrows), an interaction used to bound virus to glycan epitopes of host cells (http://pdb.org, code 3HTO).

Glycan‐decorated AuNPs may also be used for the rapid evaluation of viral hemagglutinin´s specificity because viral pathogens use lectins encoded by their own or host genome to replicate and spread^85^; these AuNPs can also be used in antiviral drugs strategies. In general, avian‐adapted influenza viruses prefer α2,3‐linked sialic acid (ligands commonly found in the intestinal epithelia of birds), and human‐adapted viruses bind preferentially to α2,6‐linked sialic acid. Using host cell receptors as biorecognition elements immobilized on AuNPs, it was possible to easily and rapidly evaluate a potential threat to the human host without using complex immunoanalytical strategies. Since influenza hemagglutinin molecules contain several binding sites (Fig. 3), the aggregation of NPs occurred with a red‐to‐purple shift.^86^

This is an alternative to using highly specific monoclonal antibodies, where an LOD of 7.8 hemagglutinin units (i.e., 7.8×10^6^ particles) was achieved recently for human H3N2 influenza A virus without any additional amplification.^87^ By using the epitope glycan structures, however, knowing the exact influenza subtype is not required. Moreover, glycans‐based biosensors can benefit from the multivalent character of the protein—glycan bond.^88^, ^89^, ^90^ For instance, NPs modified with thiolated monovalent and trivalent α2,6‐linked sialic acid and thiolated polyethyleneglycol (PEG) were successfully used to discriminate between human influenza virus X31 (H3N2) and avian RG14 (α2,3‐sialic acid binding H5N1 strain) virus with an LOD of 2.55 μg mL^−1^. In addition, the trivalent configuration provided more rapid results and a greater sensitivity for plasmonic detection relying on glycoNPs’ aggregation compared to a monovalent configuration.^91^ As shown in this and other studies, when self‐assembled monolayers (SAMs) on gold surfaces are used, the concentration of a diluting thiol as well as its functional head group and length are crucial for assay performance. Finally, using glycan‐modified nanostructured surfaces, it is also possible to reliably detect any new and, for people, potentially pathogenic viral strains without needing any other reagents just by investigating the specificity of the viral strains toward the human glycan epitopes present in exposed epithelia. SAMs as a form of nanotechnology^4^ allow the fine‐tuning of other surface properties, such as wettability and adsorption processes,^92^ in addition to tuning interfacial chemical reactivity toward the subsequent covalent immobilization of biomolecules.

#### 2. Electrochemical Biosensors

**Figure 4:**
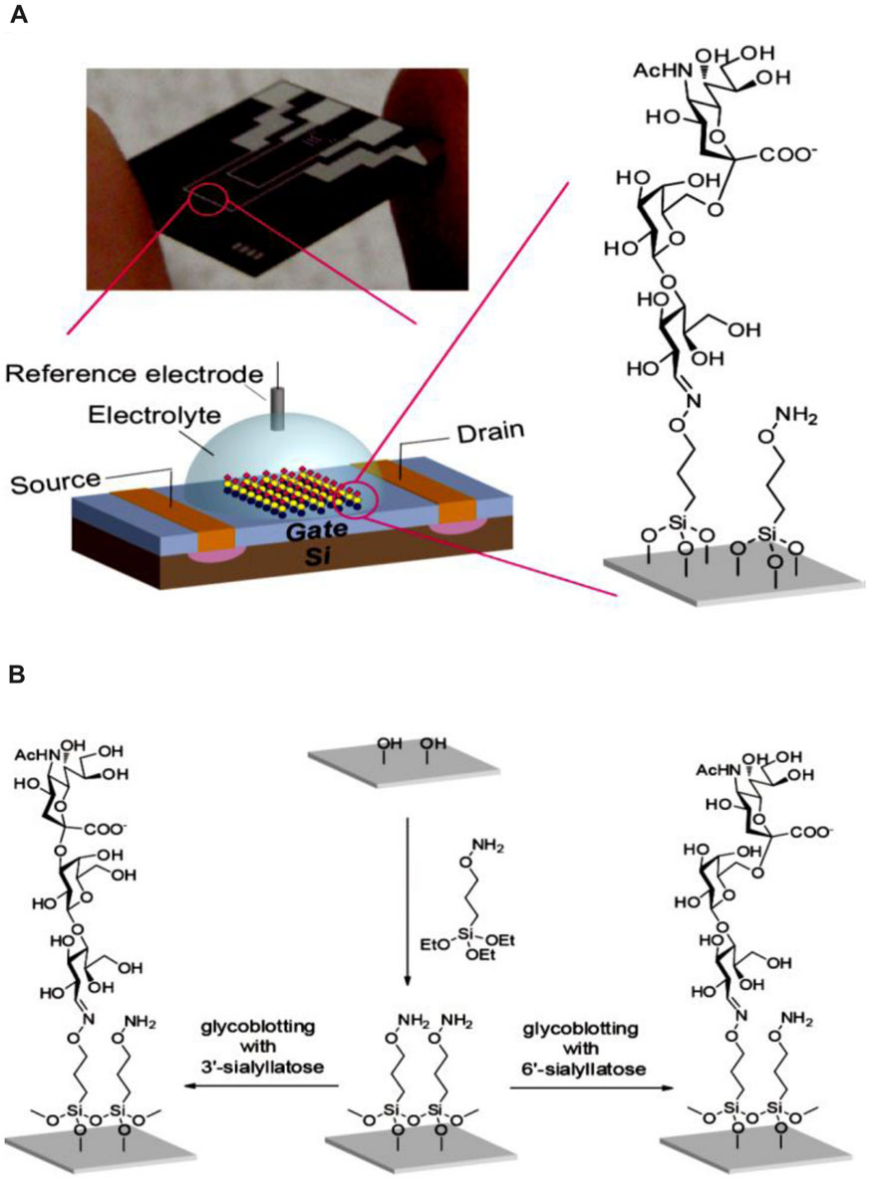
A field‐effect transistor (FET) biosensor device for detection of viral hemagglutinins down to attomolar level using glycoblotting protocol; (A) typical scheme and picture of the described device and (B) modification scheme of FET device surface using silane chemistry. The slight difference in the glycan epitope (3´‐ vs. 6´‐sialyllactose) is enough to distinguish between *Influenza* viruses able to infect a human and other types of *Influenza*. Reprinted with permission from 97. Copyright 2013 American Chemical Society.

Among others, electrochemical methods (e.g., increasingly popular electrochemical impedance spectroscopy [EIS], suitable for single‐molecule to whole‐cell detection)^93^ are highly sensitive for providing an alternative platform for viral hemagglutinins or whole‐particle detection down to attomolar detection limits or single‐viral particles, respectively.^94^, ^95^ Tung et al. prepared a nanostructured (gold nanohemisphere‐modified) biosensor surface.^96^ The impedimetric biosensor with enhanced sensitivity due to increased surface reaction area was used for the ultra‐weak binding of C‐type lectin domain family 5, member A and mosquito‐borne dengue virus particles, causing hemorrhagic fever and shock syndrome, with tens of millions infected people every year.^96^ Another ultrasensitive attomolar detection of human H1 and avian H5 viral hemagglutinin was successfully performed on a field‐effect transistor (FET) based device (Fig. 4), modified with 3‐aminooxypropyltriethoxysilane (using standard silane coupling) and the simple glycan blotting of two different trisaccharide receptors (α2,3‐ and α2,6‐sialyllactose).^97^ It is worth mentioning here that the above‐mentioned PEG molecules have become a gold standard for surface modification (even in a microarray format)^98^ for the suppression of nonspecific interactions and for the stabilization of NPs when used for biosensing applications.^99^ However, the introduction of glycan moieties, such as *N*‐acetylglucosamine (GlcNAc) and lactose, on the surface of AuNPs and AuNRs was proposed to be an alternative to PEG stabilization, ensuring colloidal stability in protein‐rich media and preventing phagocytosis by macrophages, but at the same time exhibiting an excellent sensitivity toward carbohydrate‐binding proteins.^100^ In addition to PEG, zwitterionic thiol derivatives (e.g., carboxy‐ or sulfobetaine derivatives in single‐component SAMs and mixed binary SAMs)^101^, ^102^ are promising ways to prepare nanostructured interfaces resisting nonspecific interactions.^103^

The multivalent amplification effects of glycan—receptor interactions are naturally present in biological systems, for example, complexly branched glycans on glycoproteins or densely packed rafts of glycolipids. Therefore, synthetic multivalent saccharides (among others) have often been used to mimic nature.^104^ Good examples are synthetic polymers, such as poly(acrylamidophenyl‐α‐mannose‐*co*‐acrylamide) attached to AuNPs,^105^ layer‐by‐layer modifications of MNPs coated with polysaccharide shells of hyaluronan and chitosan.^106^ These polymers were applied for the selective enrichment of glycoconjugates in more complex biological samples^106^ and for the non‐covalent modification of hydrophobic, dodecanethiol‐stabilized AuNPs (synthesized by Brust—Schiffrin method^107^) by multivalent glycocalixarenes with four mannose units to improve targeting efficiency toward intact cells.^108^ Glycan parts of different enzymes frequently serve as models to study interactions with lectins. A practical application for the food industry and agriculture is an electrochemical device based on palladium NPs (PdNPs) as catalysts for the 3,3´,5,5´‐tetramethylbenzidine sulfate/H_2_O_2_ system with immobilized mannose‐binding jacalin‐related lectin from rice (*Oryza sativa*, a bioprobe) for the electrochemical detection of *Magnaporthe oryzae* (also *M. grisea*) chitinase (a biochemical marker) during early rice infections. The use of a magnetic‐controllable electrode together with magnetic bead based PdNPs allows the detection of chitinase down to 17 pg mL^−1^ (approximately 420 fM). Moreover, using chronopotentiometry, it was possible to detect chitinase 2 days after rice infection, while a standard enzyme‐linked immunosorbent assay (ELISA) could detect the chitinase only 4 days after infection.^109^ In addition to NPs and nanorods, nanoporous materials, for example, nanoporous gold in combination with square‐wave voltammetry (SWV)^110^ and nanoporous gold monoliths in combination with thermogravimetric analysis,^111^ were used to effectively and sensitively detect glycan—protein interactions, for example, to detect high‐mannose glycan‐containing ovalbumin molecules.^112^

**Figure 5:**
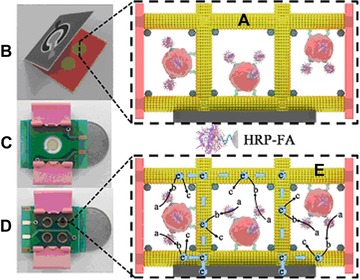
Schematic presentation of a macroporous Au paper electrode (μPECD); (A) cells are incubated with HRP‐folic acid conjugate, (B) folding of μPECD and clamping the μPECD between circuit boards (front (C) and reverse side (D)) and finally the detection principle (E), where *a* is *o‐*phenylenediamine and H_2_O_2_, *b* is 2,2´‐diaminoazobenzene and *c* is the final product of the reaction. The real size of the device is compared to a Chinese 1 yuan coin. Reprinted from 115. Copyright 2014, with permission from Elsevier.

In situ glycosensing on the cell surface may play an important role in determining the physiological status of the whole cells in the biopsy samples acquired from patients suffering from various diseases. EIS was used to detect human colon cancer DLD‐1 cells with an LOD of 40 cells mL^−1^ by bovine serum albumin (BSA) incorporated Ag nanoflowers on a glassy carbon electrode (GCE) with a 3D porous architecture and a large surface area and the retention of immobilized cells activity after binding (attributed to the presence of BSA as a biocompatible support).^113^ After the conjugation of the cells with the selected lectin (SNA in this case), the average number of sialic acid molecules on a single living cell was counted as approximately 2.16 × 10^12^.^113^ Su et al. developed a novel lab‐on‐a‐paper device for the electrochemical sensing of K562 cancer cells with an LOD of 400 cells mL^−1^ and a wide linear range spanning 5 orders of magnitude based on a macroporous Au‐paper electrode.^114^ Given that the volume used for incubation was as low as 10 μL of cell suspension, the LOD of the device was approximately 4 cells. An in situ monitoring of multiglycan expression in response to drug treatment was achieved using differential pulse voltammetry (DPV) and horseradish peroxidase (HRP) labeled wheat germ agglutinin (WGA), peanut agglutinin, *Dolichos biflorus* agglutinin, and Con A. A similar device designed by the same group later that year based on an aptamer modified 3D macroporous Au‐paper electrode was developed in a microfluidic format to screen anticancer drugs (Fig. 5).^115^ This device could also detect as little as 350 cells mL^−1^ (very similar to a previous case, e.g., approximately four cells in 10 μL) using the biosensor signal generated using an HRP‐labeled annexin V bioprobe. This bioprobe specifically interacts with membrane phosphatidylserine molecules (in the presence of Ca^2+^ ions), whose externalization cannot proceed in healthy and necrotic cells, thus providing a highly specific response toward apoptotic cells (translocation from the inner to the outer leaflet of the membrane is an important indicator of apoptosis).^115^ Recent advances in electrochemical cytosensing amplified by nanostructures and nanocrystals were reviewed by Hasanzadeh et al.^116^

No doubt the most commonly investigated and the most commercially successful biosensors are those for glucose detection based mostly on glucose oxidase (GOx) enzyme, despite the fact that even enzyme‐free nanostructured sensors have been described.^117^ However, the synergistic effect of an NP catalyst with a conducting hydrogel heterostructure‐based interface^118^ and the dependence of the sensor performance on NP shape were demonstrated only recently.^119^ Li et al. not only described a simple glucose detection, but went even further with the imaging of intracellular glucose consumption in living cancer cells.^120^ Their system is based on apo‐GOx (an inactive form of GOx) modified AuNPs and fluorescein isothiocyanate (FITC) dextran. In the presence of glucose in the environment, the quenched fluorescence of FITC‐dextran is recovered as glucose exhibits greater affinity than dextran to apo‐GOx . The LOD of 5 nM, along with the introduction of apo‐GOx instead of GOx, means no consumption of O_2_ with subsequent H_2_O_2_ production (causing cellular damage) and makes this assay a simple, sensitive, and “biofriendly” method for various disease diagnoses and metabolomics studies.^120^ Even though glucose is an important molecule for diagnostics of different conditions and cell metabolism and its importance is highlighted in many sections throughout this review, glucose as a monosaccharide is not considered as a glycan and thus glucose‐based biosensors are not discussed here in more details.

It is quite difficult to analyze electrochemically inactive glycans and oligo‐ and polysaccharides using electrochemical methods.^121^ However, Paleček and his team recently published several papers about the deacetylation of *N*‐acetylated glycans to make the –NH_2_ groups free and thus electrochemically active^122^ and about the modification of glycans with Os(VI)L complexes for subpicomolar detection.^123^ Most recently, a paper about the label‐free electrochemical detection of interaction between Con A lectin and a glycoprotein on an atomically smooth mercury electrode was described.^124^

### B. Hybrid Nanomaterials and Nanocomposites

**Figure 6:**
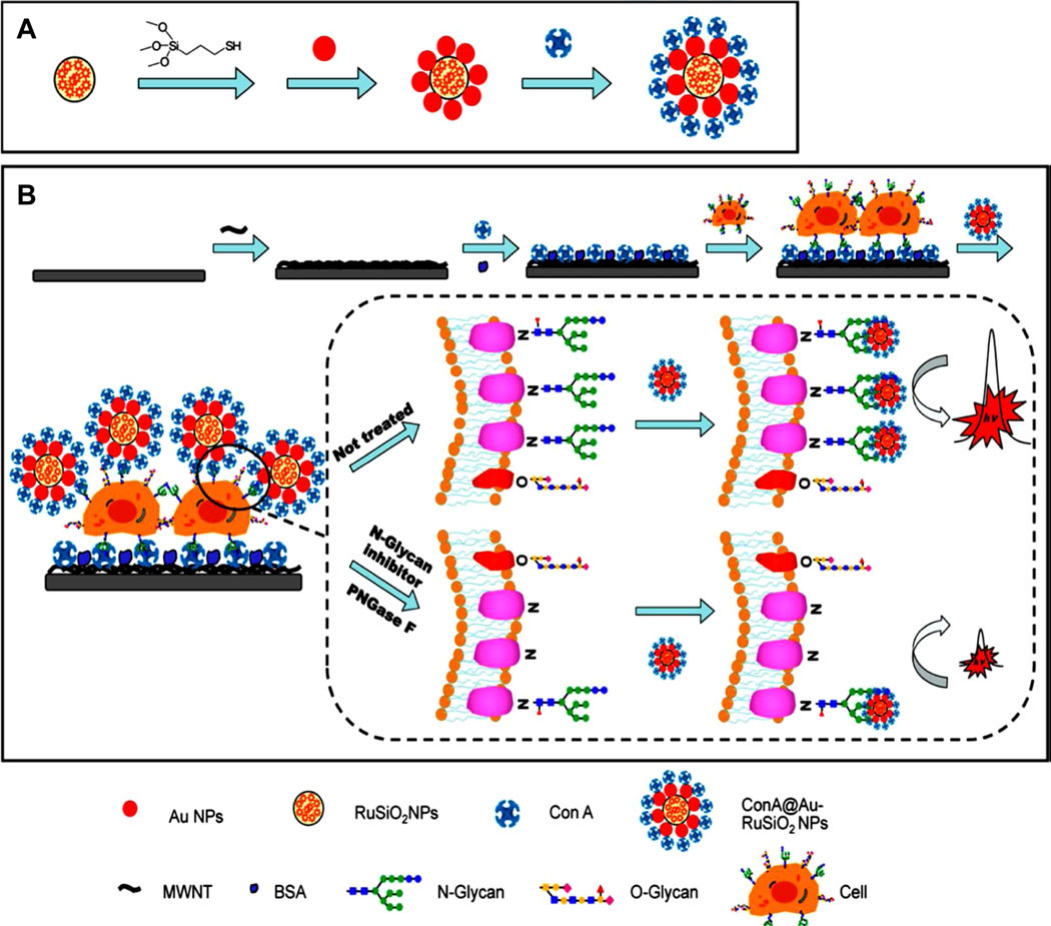
A schematic illustration of electrochemiluminiscent ECL biosensor for dynamic evaluation of cell surface *N*‐glycan expression; (A) fabrication procedures of Con A‐modified NPs presenting the lectin molecules in a multivalent manner and (B) ECL biosensor for cytosensing and evaluating cell surface *N*‐glycans, while the signal is reflecting the action of various inhibitors or glycosidases compared to untreated cells. Reprinted with permission from 127. Copyright 2013 American Chemical Society.

Very often, not only single nanomaterials but also combinations of two materials where at least one is a nanomaterial are used for bio‐ and cytosensing.^125^ AuNPs, forming novel nanocomposites with other materials, are often used as signal amplifiers. Poly(ethylenimine) (PEI)‐reduced GO (rGO) and hollow AuNPs deposited on GCE with immobilized GOx as recognition elements significantly improved the signal intensity of the luminol/H_2_O_2_ electrochemiluminiscent (ECL) system for Con A detection down to 0.31 ng mL^−1^ (approximately 3 pM).^126^ Chen et al. developed a sandwich electrochemiluminiscent biosensor with a Con A‐integrating AuNP‐modified Ru(bpy)_3_^2+^‐doped silica nanoprobe and a multiwalled CNTs (MWCNTs) modified electrode with another Con A on the surface (Fig. 6).^127^ They were able to detect myelogenous leukemia K562 cells with an LOD of 600 cells mL^−1^ and, more importantly, to dynamically observe the cell surface glycoprofile during different phases of growth in vitro in response to external stimuli—glycan release by peptide‐*N*‐glycosidase (PNGase) F or incubation with the *N‐*glycan inhibitor tunicamycin.^127^

**Figure 7:**
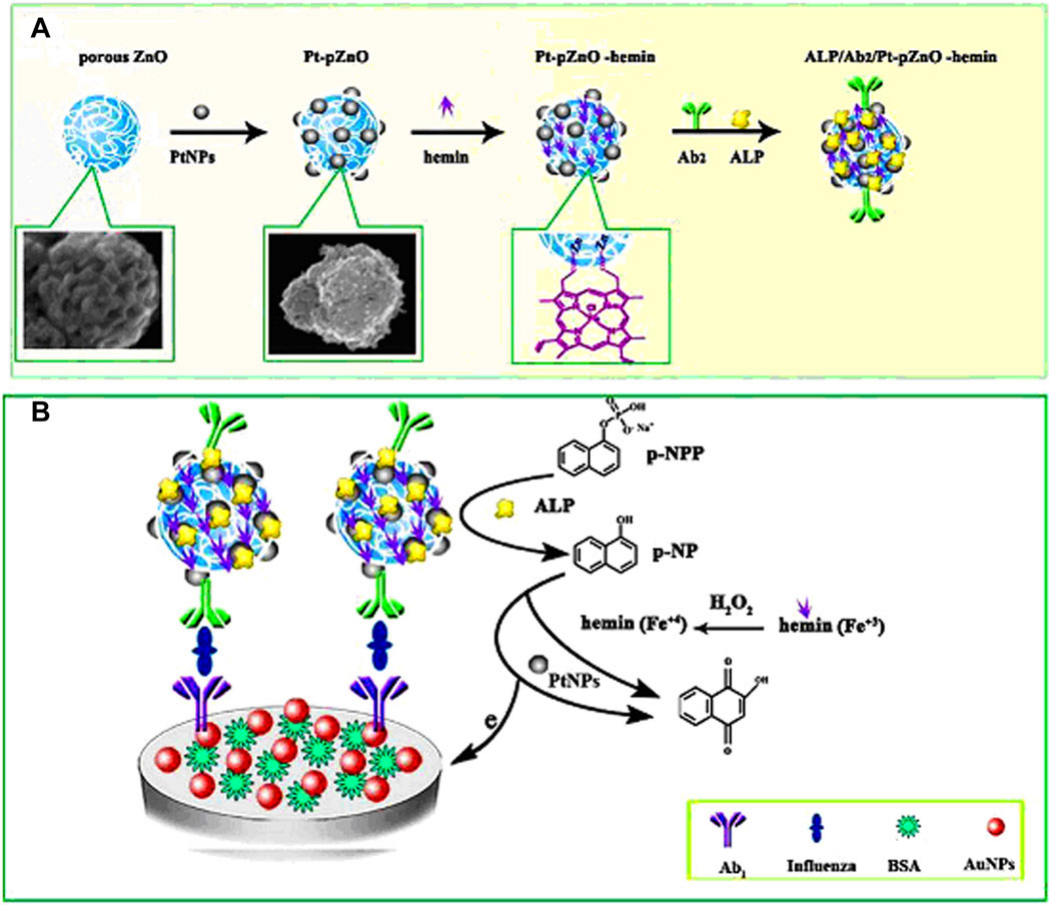
(A) A scheme for preparation of a Pt‐pZnO‐hemin conjugate with a secondary antibody (green) and alkaline phosphatase (yellow). (B) A working principle of the proposed biosensor for influenza antigen detection using a primary antibody (purple) immobilized on an AuNPs modified electrode. Reprinted from 130. Copyright 2016, with permission from Elsevier.

The same team later that year published another study describing competitive recognition and a signal amplification strategy using AuNPs modified with GOx.^128^ They counted all of the mannose moieties on a single K562 cell (1.8 × 10^10^) and again demonstrated the importance of the multivalent character of glycan—protein interactions, as the apparent dissociation constant between GOx‐Au and Con A nanoprobes was 1.64 nM—approximately 5 orders of magnitude lower than in the interaction of Con A with mannose.^128^ The low LOD for K562 (50 cells mL^−1^ with a working volume of 200 μL and a linear response of up to 800 cells mL^−1^) was achieved using a graphene‐hemin‐AuNRs ternary composite as a peroxidase mimetic.^129^ For influenza detection, a nanohybrid of the Pt NPs (PtNPs), porous ZnO spheres and hemin was synthesized for an amplified electrochemical immunosensor (Fig. 7).^130^ Briefly, by the in situ generation of a redox probe by alkaline phosphatase (i.e., the release of 1‐naphthol from inactive 1‐naphthyl phosphate) and the excellent behavior of the Pt‐pZnO‐hemin nanocomposite applied as a signal enhancer, the influenza antigen was successfully detected on an antibody‐modified electrode in a sandwich configuration using DPV with an LOD of 0.76 pg mL^−1^ and with a linear range spanning 4 orders of magnitude.^130^

More recently, He et al. introduced a novel sandwich strategy for a dual‐potential responsive, ECL biosensor for simultaneous cytosensing and surface *N*‐glycan evaluation.^131^ At a potential of 1.25 V, chemiluminescence was generated by Ru(phen)_3_^2+^ ECL probes intercalated in the grooves of double‐stranded DNA consisting of a DNA aptamer for MCF‐7 (breast cancer) cell recognition and a complementary capture DNA strand and immobilized on electrochemically reduced MoS_2_ nanosheets. In the presence of cells, the capture DNA and the ECL probe were released from the electrode interface. The sandwich was then completed by a Con A‐conjugated AuNP‐modified graphite‐C_3_N_4_ to detect cell surface mannose units at a negative potential of –1.6 V.^131^ Zhang et al. published a paper on an ECL biosensor based on PEI‐rGO and hollow AuNPs.^126^ The interaction between AuNPs and the—NH_2_ groups of PEI was used for AuNP and GOx immobilization in this case, where GOx served as a producer of H_2_O_2_ for the luminol/H_2_O_2_ ECL system. In the presence of Con A, a decrease in the ECL intensity was observed, with an LOD down to 310 pg mL^−1^ (approximately 3 pM) and with a linear range from 1 to 20 ng mL^−1^. The authors claimed that they developed an assay with a nearly 1000‐fold improved detection limit for Con A compared to previously published methods.^126^ The same team prepared a similar device using a nanocomposite consisting of C_60_ fullerene and rGO as a detection interface and hollow Au nanosphere‐conjugated GOx as a label.^132^ The interaction of GOx with the electrode interface was mediated by phenoxy‐derivatized dextran, which served as a recognition element for Con A. Using a luminol/H_2_O_2_ based ECL system, the LOD for Con A was estimated to be 30 pg mL^−1^ (approximately 288 fM) with a linear range spanning 3 orders of magnitude (from 0.1 to 100 ng mL^−1^).^132^

Multivalent recognition and dual‐signal amplification strategies with Con A‐conjugated poly(amidoamine) (PAMAM) on a chemically rGO interface and HRP‐aptamer‐AuNPs nanoprobes were reported to detect CCRF‐CEM (human acute lymphoblastic leukemia) cells down to 10 cells mL^−1^ with excellent selectivity and could dynamically evaluate surface *N*‐glycans.^133^ Graphene could also be used as a support in combination with other metal NPs for biosensing applications. For instance, an rGO‐ and AgNP‐based nanocomposite was used as a redox probe together with phenoxy‐derivatized dextran and GOx as biorecognition elements for sensitive Con A detection on GCE.^134^ Different electrochemical techniques, that is, cyclic voltammetry, DPV, and EIS, were used for signal generation with an LOD for Con A as low as 0.67 ng mL^−1^ (approximately 6.44 pM) and with a linear range from 2.0 to 322 ng mL^−1^. Furthermore, the device was successfully used in diluted real human sera with recoveries from 92 to 108% and showed no major interference from BSA, cytochrome c, or phytohemagglutinin, suggesting possible applications for rapid and reliable clinical diagnostics.^134^ Another example of the application of a nanocomposite for biosensing purposes is an rGO‐tetraethylene pentamine‐1‐butyl‐3‐methylimidazolium hexafluorophosphate hybrid composite.^135^ A dense adsorption of bimetallic AuPtNPs, subsequently used for SNA lectin immobilization, was achieved by free –NH_2_ groups from tetraethylene pentamine. This biosensor was used for the electrochemical detection of α2,6‐sialylated glycan down to 3 fg mL^−1^ and showed a wide linear range covering 8 orders of magnitude. As in a previous case, the recovery when analyzing real human sera was very similar, that is, with a range from 100.8 to 101.4% for Neu5Ac‐α2,6‐Gal‐β‐MP glycoside (4‐methoxyphenyl group *via O*‐glycosidic linkage) spiked to a final concentration of 1 pg mL^−1^ to 100 ng mL^−1^.^135^

Because α2,6‐sialylated glycans might play an important role in clinical diagnostics using various biomarkers (e.g., on PSA),^136^ new methods for their ultrasensitive detection are still emerging.^137^ For example, the nanocomposite composed of graphite oxide, Prussian blue, and PTC‐NH_2_ (ammonolysis product of 3,4,9,10‐perylenetetracarboxylic dianhydride) was used on a GCE to immobilize AuNPs through free –NH_2_ groups and SNA‐I lectin for the DPV analysis of α2,6‐bound sialic acid on serum glycoproteins down to 0.03 pg mL^−1^ with a linear range spanning 5 orders of magnitude.^138^ Although many different and successful strategies and protocols have been proposed relaying on different electroanalytical approaches for the analysis of complex glycan structures, the main challenge to be addressed for the application of affinity biosensors for real sample analysis is the efficient blocking of nonspecific interactions on the biorecognition interface, although BSA could be effectively applied as a blocking agent in some cases. Additionally, it is necessary to note that despite the fact that SNA lectin is routinely used to detect α2,6‐bound sialic acid, according to some specialized vendors, there is a minor α2,3‐sialic acid binding activity present as well.^139^

### C. Carbon Nanomaterials

Engineered carbon nanomaterials (nanotubes, graphite, fullerenes, and graphene as main matrices for the conjugation of biomolecules) can be successfully applied for the preparation of biosensors.^140^ There are different conjugation techniques for the functionalization of carbon structures with carbohydrates; the most common is the use of a carboxylic group formed on carbon surfaces using strong acidic oxidation with the subsequent conversion of –COOH groups to acyl chlorides, direct (carbodiimide activated) amidation, or ligation with an azide (Staudinger ligation).^141^ Single‐walled CNTs (SWCNTs) were also functionalized through microwave‐assisted functionalization using perfluorophenyl azides with mannose and galactose.^142^ This interface provided a reliable platform for agglomeration studies using FITC‐Con A lectin, specifically binding to α‐d‐mannopyranoside and, to a lower extent, to α‐d‐glucopyranoside residues, but not to Gal‐modified SWCNTs.^142^ Through phenylacetylene‐SWCNTs and few‐layer graphene flakes, Ragoussi et al. prepared a carbohydrate‐modified (α‐d‐mannosyl glycodendron‐bearing) carbon nanostructures for Con A detection (using AFM, fluorescence, and UV/VIS studies), connected by means of CuAAC “click reaction” mechanism.^143^ This group even managed to capture and observe the same object for their AFM study, leading to a reliable height profile analysis of the nanostructures before and after treatment.^143^ Other detection platforms may be useful for studying glycan—lectin interactions using graphene‐modified surfaces. For SPR‐based experiments, graphene was grown through chemical vapor deposition (CVD) on polycrystalline Cu foils in a five‐step process on a 50‐nm‐thick Au film as single and double layers.^144^ A simple immersion of this interface into a 100 nM mannose solution was sufficient for the mannose modification of the interface through the interaction of carbohydrate with the aromatic ring structure of graphene. Using mannose‐specific *Lens culinaris* agglutinin and GlcNAc‐ and sialic acid‐specific *Triticum vulgaris* agglutinin, it was shown that noncovalent surface modification by a simple mannose adsorption allowed the tuning of surface selectivity towards a specific receptor in a simple manner, allowing an LOD of approximately 1 μg mL^−1^ (low nM range) for lectins with a linear range of up to 1000 μg mL^−1^.^144^ However, electrochemical and ECL‐based devices are more sensitive than optical platforms.

Since the Nobel Prize in physics in 2010, graphene has become an increasingly popular material and has recently been applied for biosensing purposes, as well as for glycomics, mainly in cytosensing applications using different detection strategies. As already discussed in Section B..B regarding nanohybrids, the combination of different materials, the preparation of nanocomposites, and the very frequent utilization of a sandwich format analysis is common for biosensor construction. It has been previously reported that polymer dendrimers (e.g., PAMAM in this case) provide excellent support for the immobilization of glycans, allowing them to interact with proteins involving a multivalency effect. Molecular recognition using so‐called corona‐phase complexes consisting of synthetic polymers and CNTs, where the two components show affinity toward a selected analyte only if they stick together via surface forces stabilizing them and giving the polymer its final configuration (with a possibility to predict recognition specificity in advance), was also reported.^145^ SPR gold surface coated with rGO (electrophoretically deposited) can be easily modified by a simple immersion in a solution of a particular polymer to prepare strongly negative (poly(sodium 4‐styrenesulfonate)) or positive (PEI) surfaces, as well as a surface modified by different saccharide moieties (mannose, lactose) through π–π stacking and electrostatic interactions. Subramanian et al. modified SPR chips with rGO to study the affinity of three different pathogenic *E. coli* strains to surfaces mediated by the presence of different adhesins on a bacterial cell membrane because those are responsible for the colonization of different epithelial structures and surfaces.^146^ The modified SPR interface interacted strongly with highly pathogenic *E. coli* 107/86 strain in a quantitative manner with a linear response spanning 7 orders of magnitude and with an LOD of ∼100 cfu mL^−1^ (cfu—colony‐forming units) for bacterial strains 107/86 and UTI89.^146^

ECL methods based on carbon nanomaterials could be used for cytosensing applications, as well. The simplest sandwich configuration, in which GO‐modified GCE served to immobilize the antibodies of interest (anti‐PSA in this case for the specific biorecognition of membrane PSA) and with a subsequent surface blocking by the use of BSA, was used to detect PC‐3 (prostate cancer) cells down to 260 cells mL^−1^.^147^ With a linear range spanning almost 2 orders of magnitude, ruthenium complex‐labeled WGA served as a signal probe.^147^ A similar concept used for the impedimetric detection of HL‐60 (human promyelocytic leukemia) cells down to 500 cells mL^−1^ was based on a graphene surface modified by carboxymethyl chitosan.^148^ This composite served to support the layer‐by‐layer assembly of PEI and folic acid for the fabrication of a label‐free cytosensor. Folic acid served as a biorecognition element because the overexpression of folate receptors often occurs in some tumor cell lines.^148^

Graphene may be successfully utilized in various forms in biosensing technologies using not only plane graphene sheets but also monolithic and macroporous graphene foam. Such a 3D matrix (grown by CVD) was used to prepare an immunosensor for carcinoembryonic antigen (CEA, a tumor biomarker).^149^ Briefly, a graphene substrate was used for the polymerization of dopamine, which subsequently served as a matrix for noncovalent Con A immobilization and interaction with HRP‐labeled anti‐CEA as a biorecognition element bound to Con A via a glycan part of HRP. After the surface was blocked by HRP, various electrochemical methods (mainly DPV using an electrochemical mediator) were used to detect CEA down to 90 pg mL^−1^ (approximately 500 fM), and the biosensor did not show any response toward other biomolecules, such as BSA, PSA, HRP, or glucose. Noncovalent graphene modification could be achieved not only by unmodified saccharides,^144^ but as well as using “clickable” monosaccharide derivatives, such as azido galactosides immobilized on an alkynyl anthraquinone‐modified graphene electrode for the label‐free EIS detection of cancer cells.^150^

### D. Other Nanostructures, Glycopolymers, and Boronic Acid Derivatives

Commonly used nanomaterials in glycomics in addition to metal and carbon nanostructures are glycopolymer‐based micelles, vesicles, or nonspherical NPs that are able to interact with lectins as multivalent ligands in a manner similar to natural glycoproteins.^48^ Block copolymers often self‐assemble into diverse morphologies in solution depending on their properties, providing a promising bottom‐up engineering strategy for different applications of such nanostructures.^61^ Polymer scaffolds may also be effectively glycosylated in vitro using a wide variety of available glycosyltransferases to prepare glycan structures mimicking those present in nature for the biorecognition of multivalent glycans by their specific lectin receptors.^151^ Any information contained in the “sugar code” must be controlled with an extreme precision during glycopolymer preparation in laboratories for diagnostic and other purposes because every small difference in vivo may significantly affect a biorecognition event and lead to structural and functional abnormalities in the organism. Therefore, structural control of carbohydrate sequences during the synthesis of glycomimetics and multivalent glycopolymers is of highest importance to obtain reliable data.^152^, ^153^ Such synthetic glycopolymers are promising tools for use in emerging biomedical applications and research, including biosensing, biomolecular recognition, and vaccine development.^154^, ^155^

Multivalency and complexity of lectin—glycan interactions are applied in numerous processes in nature. For the study of such a complex combination of binding mechanisms in real time, dendrimers may serve as useful tools to evaluate the binding capacity of lectin receptors and the effect of avidity. Mannosylated gallic acid‐triethylene glycol‐based dendrimers in combination with SPR provided important structural data for studying biorecognition between Con A and mannose‐modified dendrimers.^156^ An amphiphilic block copolymer consisting of hydrophilic lactose and hydrophobic pyridine was synthesized via reversible addition‐fragmentation chain transfer polymerization.^157^ Glycosurface prepared on Au quartz crystal microbalance (QCM) chips was used to reliably detect RCA_120_ in the nanomolar concentration range without any significant binding of BSA as a nonspecific probe.^157^ Moreover, the authors in this study calculated the *K*_A_ for the system, obtaining a value of 6.3 × 10^6^ M^−1^; this value is normally in the range of 10^3^ M^−1^ for monovalent lactose and its receptor. A similar value of *K*_A_ (same order, 2.3 × 10^6^ M^−1^) was obtained in another study using QCM and RCA_120_ lectin binding to galactose‐containing gradient glycopolymer synthesized by RAFT polymerization.^158^ By synchronizing enzymatic monomer transformation with polymerization, the authors obtained a gradient sugar distribution in a final amphiphilic polymer.^158^ The lowest detectable concentration (5 μg mL^−1^) was again in the low nanomolar region. Superior lectin binding was achieved for the gradient polymer compared to the statistical glycopolymer, underlining the relevance of multivalency in the case of lectin—glycan interactions.^158^ Glycoconjugated amphiphilic polymers can also be used for the encapsulation of fluorescent QDs.^159^ Prior to its encapsulation, amphiphilic poly(isoprene)‐*b*‐poly(ethylene glycol) diblock copolymer was covalently modified by a carbohydrate moiety (d‐manno‐heptulose, d‐glucose, d‐galactose, bis(nitroso)‐streptozotocin, or d‐maltose) using Huisgen‐type click chemistry, and interaction with Con A was studied again using the SPR method, showing enhanced affinity constants due to multivalent binding effects.^159^ Supramolecular structures, which are of high importance in nanotechnology these days, may also be prepared by click chemistry reactions, as in the work published by Assali et al., in which the authors managed to synthesize poly(diacetylene)‐based nanomaterials with different morphologies.^160^ Neoglycolipids with an amide bond between the hydrophilic and hydrophobic parts of the amphiphilic molecule formed 3D micelles, while triazole‐containing ones (obtained by “click‐reaction”) allowed 1D nanotube formation.^160^ Block glyco‐copolymers may also be used for cell imaging and as an effective drug delivery system (see Section 6.). They may also enhance the uptake of drug‐loaded micelles by cells, as in the case of the increased uptake of doxorubicin‐loaded sugar (glucose or maltose as a biorecognizable hydrophilic block modification) and poly(4‐substituted‐ε‐caprolactone) copolymer micelles by HeLa cells, compared to free doxorubicin.^161^

Since glycans are the most complex biomolecules, there is a need for high‐throughput methods for their analysis. In addition to commercial microarrays, a novel super‐microarray (containing many microarrays on the same slide) for lectin glycan sensing was recently developed. Such arrays use glycan‐labeled dye‐doped silica NPs (SiNPs) and a set of lectins immobilized on epoxy slides with poly(dimethylsiloxane) as an insulator, allowing the generation of many individual lectin microarrays, which significantly increase the assay throughput and, due to the multivalency of glycan‐modified NPs, also increase the affinity (over the free glycan and corresponding lectin) by 4–7 orders of magnitude.^162^ Moreover, fluorescently labeled NPs offer higher stability and fluorescence compared to free organic dyes. Although glycans‐modified NPs have previously been prepared, the first attempt to prepare carbohydrate‐modified SiNPs was published recently by Ahire et al.^163^ d‐mannose‐capped SiNPs (prepared from amine‐terminated NPs using N,N´‐dicyclohexylcarbodiimide) were used to detect Con A when the interaction caused the aggregation of NPs. To show their biochemical activity, the photoluminescence of these NPs after interacting with MCF‐7 human breast cancer cells was also investigated.^163^ For electrochemical analysis, conductive polymers are highly relevant for the enhanced sensitivity of detection when used as a solid‐state redox probe. Thiophene containing fused quinone moieties were electrochemically polymerized on a gold electrode surface to couple thiol‐modified mannose.^164^ Such electropolymerization created a thin film on a solid surface with the ability to control its thickness very precisely up to several nanometers with subsequent application to construct microsensors. This new glycosurface allowed the detection of two major bacterial cell surface biomarkers—namely, fimbriae proteins on bacterial pili and lipopolysaccharides (LPSs) on G^‐^bacteria (by Con A‐mediated binding), using SWV and QCM methods down to 25 and 50 cells mL^−1^, respectively.^164^ Moreover, it was quite simple using this method to selectively distinguish between G‐ and G+ bacteria.^164^

### E. Synthetic Receptors for Glycosensing

Common biorecognition elements for the sensitive detection of various analytes (biomolecules, viruses, or even bacteria) include antibodies and less common nucleic acid aptamers. In the past decade, carbohydrates have been increasingly studied due to their presence on the surfaces of proteins and cells. For the purpose of glycocode deciphering, lectins from various sources are commonly used.^22^ Boronic acids also bind saccharides via reversible interactions, mostly with linear diols or even *cis*‐1,2‐diols on five‐membered rings or 1,3‐diols to form five‐ or six‐membered rings.^165^, ^166^ Fluorescent diboronic acid compounds with dipeptide linkers were synthesized to discriminate cell‐surface Lewis X (Le^x^) trisaccharide present on Chinese hamster ovary (CHO) CHOFUT4 cells at micromolar concentrations.^167^ The control cells (without glycan expression, HEP3B cells predominantly expressing Le^y^, B16FUT3 cells expressing sialyl Lewis a (Le^a^) and COLO205 cells expressing sLe^x^ and sLe^a^ but no Le^x^) were not labeled,^167^ suggesting the possibility of preparing compounds with a specificity toward glycans comparable to that of naturally occurring lectins. As previously mentioned (Section 4.A.2), pathogenic agents, such as viruses and bacteria, use their envelope proteins (agglutinins) and adhesin lectins to recognize and attach themselves to host cells and tissues via glycans. This principle was used to prepare a novel electrochemical displacement sensor based on three different boronic acid derivative tracers (containing a ferrocene molecule).^168^ The displacement of tracers by Con A lectin molecules or *E. coli* cells led to a decrease in the electrochemical signal monitored by SWV. Moreover, the use of thiolated mannose‐OEG conjugate ensured low nonspecific interactions. Con A could be detected with an LOD of 1 μg mL^−1^ (approximately 9.6 nM, with a linear range spanning ∼2 orders of magnitude), and *E. coli* cells could be counted down to 600 cells mL^−1^.^168^ The novel tracer used in this study, 2‐hydroxymethyl phenyl boronic acid derivative, binds to mannose even at a neutral pH, expanding the application of the system toward real biological samples (e.g., urine).^168^ To date, many synthetically prepared “boronolectins” showed only a moderate fluorescence enhancement with a requirement of significant amount of co‐solvents in aqueous solution (i.e., dimethylsulfoxide and ethanol). A newly engineered boronolectin derived from tricarbocyanine combined with a boronic acid fragment linked by a piperazine unit exhibited improved certain properties, such as excellent water solubility and sensitive fluorogenicity, upon binding to carbohydrate moieties under a physiological pH.^169^ To conclude, because boronic acid derivatives are able to successfully mimic lectins as natural glycan decipherers, they may be used not only to detect various analytes but also to selectively bind to free viral particles to inhibit their progression and surface adhesion, as in the case of lipid nanocapsules functionalized with amphiphilic boronic acid for hepatitis C virus inhibition, similar to cyanovirin‐N or griffithsin (both potent HIV inhibitors).^170^ Their use as ultrasensitive solid‐phase microextraction probes for in vivo and in vitro sensing purposes in biofluids and even semisolid biotissues was also demonstrated.^171^

### F. Naked‐Eye Detection Using Nanostructures

**Figure 8:**
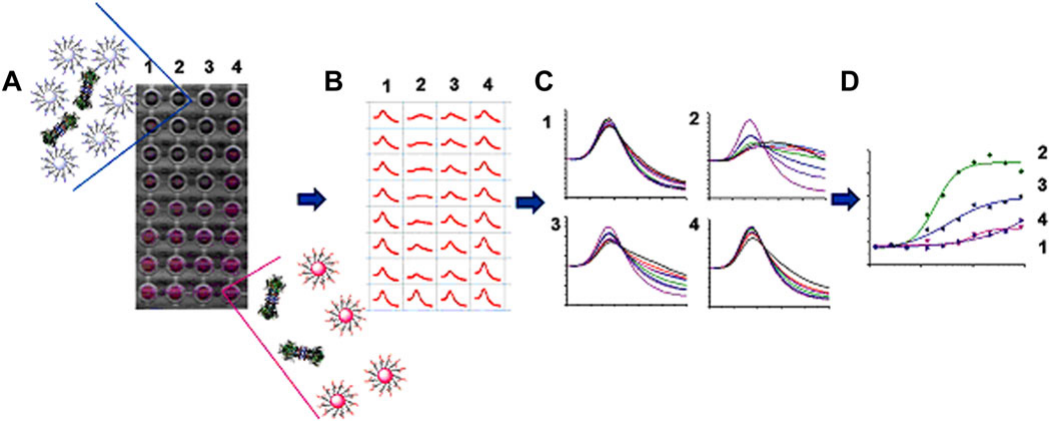
An overview of the colorimetric detection principle of lectin–glycan interactions with naked eye by glyco‐AuNPs aggregation due to lectin interaction. In case of an aggregation of NPs (in presence of lectin molecules), red‐to‐blue shift in color occurred. Reproduced from 173, with permission of the Royal Society of Chemistry.

AuNPs modified with different saccharide moieties (lactose, arabinose, cellobiose, sucrose, mannose, glucose, and galactose) were applied by Jayawardena et al. to successfully distinguish among four different lectins with different specificities.^172^ Con A, soybean agglutinin, *Griffonia simplicifolia* agglutinin, and *Arachis hypogaea* peanut agglutinin were detected by observing a red shift in the λ_max_ of the LSPR absorption (LSPR on NPs, as opposed to propagating SPR biosensors).^172^ Such a library‐oriented approach of glycan‐decorated NPs was later used to prepare polymer‐stabilized glyco‐AuNPs for a rapid, high‐throughput, and 96‐well microplate‐compatible evaluation and identification of pathogenic lectins without a need for any infrastructure because the output of these measurements (red‐to‐blue color shift upon AuNP aggregation) was monitored by a digital camera (Fig. 8).^173^ Plasmonic metal NPs thus have great potential for their use in biosensor technology due to their sensitive spectral response to the local environment of NPs.^174^

SPR, however, lacks the higher throughput capability compared to lectin microarrays. This drawback was overcome recently^175^ by establishing a lectin microarray based on a multiplexed SPR interface for the simultaneous measurement of up to 96 interactions by the immobilization of 18 different unmodified lectins (at different dilutions), including controls. A microarray GOAL (Glyco‐gold NP‐based Oriented immobilized Antibody microarray for Lectin) assay was also introduced as a novel approach for the naked‐eye detection of lectin‐carbohydrate interactions after silver enhancement using oriented, surface‐immobilized anti‐lectin antibodies.^176^ Moreover, these modified AuNPs were highly stable and resistive to any nonspecific protein adsorption.^176^

Human IgGs are extremely important markers of various diseases, which can be applied in a quantitative and qualitative manner because these glycoproteins are responsible for an effective immune response. The glycan part of human IgG was shown to be associated with autoimmune disease progression, mainly rheumatoid arthritis,^16^, ^101^ where the *N*‐linked biantennary complex glycan in the Fc region is terminated with galactose or even GlcNAc, while in healthy individuals, IgG´s glycan can be terminated with sialic acid.^177^ The GalNAc biosensor based on poly(diacetylene) nanovesicles developed by Hao et al. was applied for a noninvasive and real‐time colorimetric analysis of galactose‐deficient IgA1 (playing an important role in the pathogenesis of glomerulonephritis—IgA nephropathy) using nanovesicles modified with *Helix aspersa* agglutinin for naked‐eye detection.^178^

**Figure 9:**
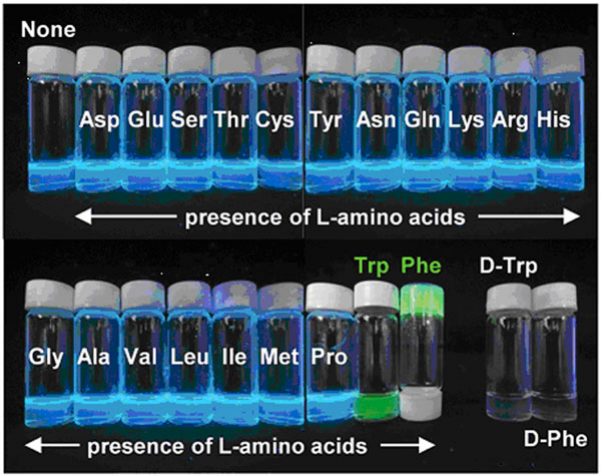
Photographs of the aqueous dispersions of the D‐vesicles (self‐assembled morphology of synthesized fluorescent glycolipid monomer c = 1.5 mmol, average of 48 nm in diameter) in the presence and absence of amino acids (80 nmol) for the naked‐eye detection. Reproduced from 179 with permission of the Royal Society of Chemistry.

In addition to glycoproteins, other glycoconjugates, such as glycolipids, were recently used for sensing applications. A fluorescent glycolipid monomer was synthesized using conjugation between 1‐pyreneboronic acid and a glycolipid based on a condensation reaction between d‐glucose and oleic acid for the qualitative and chiral sensing of 80 nmol of amino acids (l‐ and d‐tryptophan and phenylalanine) by the naked eye (Fig. 9).^179^ In order to study carbohydrate–carbohydrate or carbohydrate–protein interactions using glycolipids, three different strategies could be utilized: (i) insertion of a synthetically prepared glycolipid into a lipid matrix, (ii) preparation of glycolipids that aggregate to form liposomes or micelles, and (iii) modification of a hydrophobic surface by a desired sugar derivative.^180^

### G. Quantum Dots (QDs)

QDs have also attracted considerable attention in many different fields, including bioimaging and the detection of various analytes, mainly because of their tunable optical size‐dependent properties.^181^ In recent years, the importance of detecting various forms of viruses has emerged with a focus on the identification of various glycoforms present on viral surfaces. A two‐step procedure was developed for virus detection, including the isolation of viral hemagglutinins by glycan‐modified paramagnetic beads, labeling hemagglutinins with CdS QDs with their subsequent electrochemical detection by voltammetry using 3D printed microfluidic chips.^182^ The other detection principle employed by Chen and Neethirajan was based on a homogenous fluorescence quenching principle.^183^ They used a sandwich configuration (antibody‐modified AuNPs and glycan‐conjugated QDs) to entrap influenza A hemagglutinins in between these two probes. As a result, a fluorescence decrease due to a nonradiative energy transfer between these two probes was observed. Of course, QDs could be conjugated with a diverse range of different structures in the same way as for the other above‐mentioned nanomaterials. The conjugation of CdSeTe@ZnS‐SiO_2_ QDs modified with 3‐aminophenylboronic acid was used to monitor changes in the relative amount of sialic acid on K562 cell surfaces after a 3´‐azido‐3´‐deoxythymidine treatment, showing a significant increase in sialic acid expression.^183^ Another paper^184^ aimed to develop a photoelectrochemical biosensor using low‐toxic Ag_2_S QDs for glucose detection as well as for the detection of MCF‐7 breast cancer cells down to 32 μM and 98 cells mL^−1^.

## 5. CARBOHYDRATE‐BASED VACCINES, ADJUVANTS, AND THERAPEUTICS

With ongoing progress in glycomics, NPs displaying glycan moieties have been gradually recognized as potential therapeutic agents. There are numerous reviews mapping this broad research field. Hence, the commercialization and real application of developed glyco‐based vaccines are well documented. For example, in a recent study,^185^ a list of glycoconjugates that were already approved or used in clinical trials as vaccines is provided, while other authors have focused on the application of nanomaterials in vaccine design.^186^, ^187^

### A. Vaccines and Adjuvants

When saccharide‐based vaccines are developed using nanomaterials, the latter components are primarily applied as glycan carriers, acting as effective immunogenic moieties. The first glycan‐based vaccine was Pneumo Vax produced by Merck (Darmstadt, Germany)—an unconjugated capsular polysaccharide isolated from *Pneumonia* serotypes.^188^ Capsular antigenic glycans are in fact the only choice in the development of glycan‐based antibacterial vaccines, but the application potential of glycans given by their combinatoric diversity is very large. The immunogenic effect of these vaccines increases significantly by the increased valency of glycans employed, that is, typically by their conjugation to proteins, polymeric scaffolds, or other NPs. As already summarized,^188^ carbohydrate‐based vaccines were developed against viruses, some prokaryotes and, quite recently but with ever‐growing interest and success, even against cancer cells. Although noncarbohydrate immunogens (i.e., protein‐based antibodies) have been more broadly used for vaccine development, carbohydrate immunogens are similarly important and useful. Recent achievements in the efficient conjugation of the latter immunogens with NPs are discussed in the following sections. In addition, recently reported studies where carbohydrates are used as building blocks (not immunogens) for the development of vaccines are covered as well.

#### 1. Carbohydrate Immunogens Conjugated with Polymers

**Figure 10:**
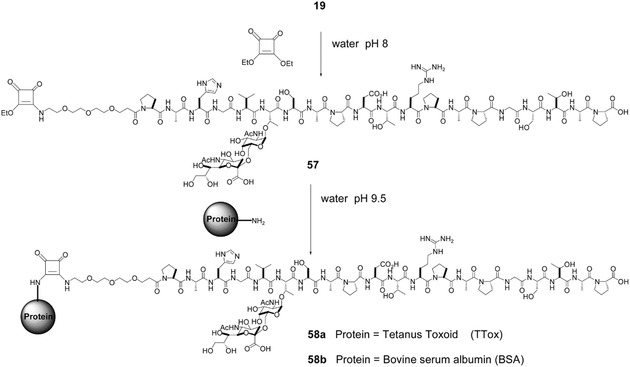
Synthesis and structure of a carrier protein‐mucin vaccine. Conjugation of a sialyl‐Tn MUC1 glycopeptide antigen (19) terminated via a coupling agent (diethyl squarate; 57) with a carrier protein (Tetanus toxoid or BSA) into a final multivalent carbohydrate‐bearing vaccination conjugate (58). Reproduced from 189 with permission of the Royal Society of Chemistry.

**Figure 11:**
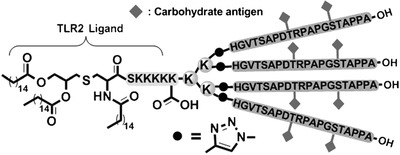
A schematic structure of a vaccine consisting of 4‐valent glycopeptide MUC1 conjugated to a bacterial lipopeptide (Pam3CSK4), a ligand of Toll‐like receptor (TLR2) which helps to elicitate immune reaction. Reproduced from 190 with permission from John Wiley & Sons.

In order to overcome the low immunogenicity of cancer cells, tumor‐specific or tumor‐associated antigens can be multiplied on the surface of NPs. The administration of such NPs helps the immune system to fight disease in a more efficient way. A multivalent display of antigens allows their better recognition by respective receptors with the subsequent induction of the immune response. It should be noted that natural antigenic carbohydrates (except for zwitterionic glycans) are believed to mostly lack the ability to be displayed on the B‐cell surface via the major histocompatibility complex, resulting in their incapability to activate T cells. This activation was, instead, achieved by displaying parts of carrier proteins co‐delivered in a vaccine. In the following sections, however, glycan‐nano vaccines without peptide adjuvants are also shown to be fabricable. Using “classical” adjuvants, the conjugation of immunogenic synthetic mucin (MUC1, a surface‐displayed glycopeptide typical for tumor epithelial cells) with different NPs was tested intensively. In a recent review paper, a study describing MUC1 conjugated with tetanus toxoid carrier protein was described.^189^ This combination provided the best performance as judged from the highest production of specific IgGs (see Fig. 10 for the structure of the glycoprotein conjugate) among other reviewed MUC1 vaccines.^189^ More recently, the same antigen was conjugated with a bacterial lipoprotein—a Toll‐like receptor ligand to boost the elicited immune reaction (Fig. 11).^190^ The same composition (i.e., MUC1 antigen conjugated to a Toll‐like receptor) amended with a T‐helper‐cell epitope was employed by Abdel‐Aal et al.^191^ Their conjugate, however, was not multivalent, but it was incorporated into small lamellar vesicles. These vesicles were used to immunize mice that were subsequently exposed to tumor induction. Animals that had been immunized with the best‐performing conjugate exhibited an approximately twofold smaller tumor after 2 weeks compared to other vaccines or a blank.^191^ Another cancer antigen is the breast cancer cell‐specific hexasaccharide “Globo H,” and its conjugation with diphtheria toxoid CRM_197_ and α‐galactosylceramide C34 as an adjuvant provided the highest immune response amongst the conjugates tested using different carrier proteins and adjuvants. This effect observed was even higher than for a similar clinical trial phase III vaccine.^192^ In order to synthesize well‐defined nanoconjugates, a method using the tyrosine‐specific binding of immunogenic glycans present on the surface of CRM_197_ was developed^193^, ^194^ with consequent application in the development of a glycan‐based vaccine eliciting an immune response against *Streptococcus* infection.^195^ Similarly, McCarthy et al. developed a chemoenzymatic synthesis of a well‐defined poly(sialic acid)‐tetanus toxoid glycoconjugate.^196^ These studies have underlined how important it is to develop novel progressive methods for the precise and well‐defined synthesis of glycoconjugates.

In addition to discovering new possibilities in the synthesis of well‐defined glycoconjugates consisting of already known components, some studies, more biochemical than nanotechnological, have focused on immunogenic efficiency and the synthesis of new poly‐/oligo‐saccharides, mainly derivatives of bacterial cell wall epitopes.^197^, ^198^, ^199^, ^200^ Similarly, Johannes et al. investigated the increased immunogenicity of fluorinated analogues of tumor‐associated carbohydrate antigens conjugated to a support applicable as a vaccine against human breast cancer.^201^

Alternatively, carbohydrates were used as building blocks or monomers for supramolecular scaffolds forming vaccine NPs. Since polysaccharides can form polyvalent ions, they can form self‐assembled nanoscaffolds upon mixing with relevant (polyvalent) counterions. Derivatives of chitin, the second most abundant polysaccharide on Earth and a very promising material in this field of research, can be used as very potent vaccine adjuvants. The mucoadhesion of chitin derivatives is helpful in vaccine administration and allows for an increased rate of chitosan‐based NP internalization via mannose‐binding receptors, inducing both the humoral and cellular immune response.^202^ The latter was found to induce the degradation of the polysaccharide NPs, and chitosan dissolved in cytosol was found to be a more efficient immunogen than whole, nondestructed chitosan NPs.^203^ The stability of prepared vaccine NPs is also an important issue. For example, a DNA‐based antitumor vaccine was effectively protected against degradation at a low pH when present in alginic acid based NPs.^204^ Similarly, the coencapsulation of an antigen to be delivered with a commercially available adjuvant (C48/80) into chitosan NPs significantly decreased the dosage needed for the induction of a strong immune response compared to vaccination with a nonencapsulated antigen and an adjuvant.^205^, ^206^ A similar effect was observed for dendritic glucan,^207^ and immunogenic properties have also been assigned to β‐glucans applied for the preparation of antifungal vaccines^208^ and to polysaccharide based on δ‐inuline, which was utilized as an adjuvant.^209^ Cholesteryl pullulan was used to entrap an additional immunogen—tumor necrosis factor α.^210^ The nasal coadministration of such a prepared adjuvant combined with commercial anti‐influenza vaccine significantly elevated the resistance of mice treated against the influenza virus compared to control groups vaccinated without the adjuvant.^210^

A better administration of tumor‐associated carbohydrate antigens was developed by Zhou et al., who investigated different derivatives of synthetic lipid A.^211^ These compounds have been previously found as promising adjuvants, but a more efficient synthesis is needed for their wider utilization.^211^ The efficacy of polysaccharide vaccines can be further improved by the decoration of NPs, with additional glycan moieties with mannose‐coated chitosan particles being a good example.^212^, ^213^ Another approach was presented by Fagan et al., who used tetra‐*O*‐acetyl‐α‐d‐glucopyranosyl bromide as a core structure for the synthesis of dendritic nanoscaffolds displaying multivalent immunogens—streptococcal B‐cell epitope.^214^

It is also possible to prepare effective conjugates based on polymers and glycans where carbohydrate moieties are not primarily acting as immunogens but rather promote selective delivery and administration. A good example is mannosylated liposomes delivering albumin as a model antigen efficiently to dendritic cells responsible for the induction of humoral and immune responses.^215^ The presence of mannose receptors on the surface of DC has also been widely used in drug delivery, imaging, and other biomedical applications that are discussed later. The overall therapeutic efficacy of mannosylated liposomes loaded with antitumor antigen and lipidic adjuvant was significantly boosted by the codelivery of small interfering RNA (siRNA) responsible for the downregulation of expression of immunosuppressive interleukins in tumor cells.^216^ In another approach, Kim et al. developed fiber‐like supramolecular assemblies coated with mannose‐tethered lectin Con A.^217^ Such protein coating was responsible for the immunogenicity of the fabricated conjugates and confirmed by observed interleukin production after the treatment of T cells with Con A‐coated NPs.^217^

Carbohydrate immunogens playing a role as adjuvants, that is, helping to elicit a less‐specific immunoresponse, could also be delivered by diverse nanopharmaceutical systems. For example, a synthetic *Mycobacterium tuberculosis* epitope derivative (a fusion protein) and a glucopyranosyl lipid moiety were tested in liposomes, nanoemulsions, and adjuvants, with a promising immune response induction observed in mice with nanoemulsions selected as the best option for further vaccine approval and trials.^218^ Controlled selective delivery was also achieved by the conjugation of a bioactive molecule (inhibitor of a transforming growth factor‐β receptor) with mannose‐6‐phosphate‐human serum albumin.^219^ This carrier reacted selectively with receptors of hepatic stellate cells, while the conjugated inhibitor prevented transforming growth factor‐β‐induced activation, which is a key factor in the development of liver fibrosis.^219^

#### 2. Metal and Metal Oxide NPs in Recent Vaccine Development

**Figure 12:**
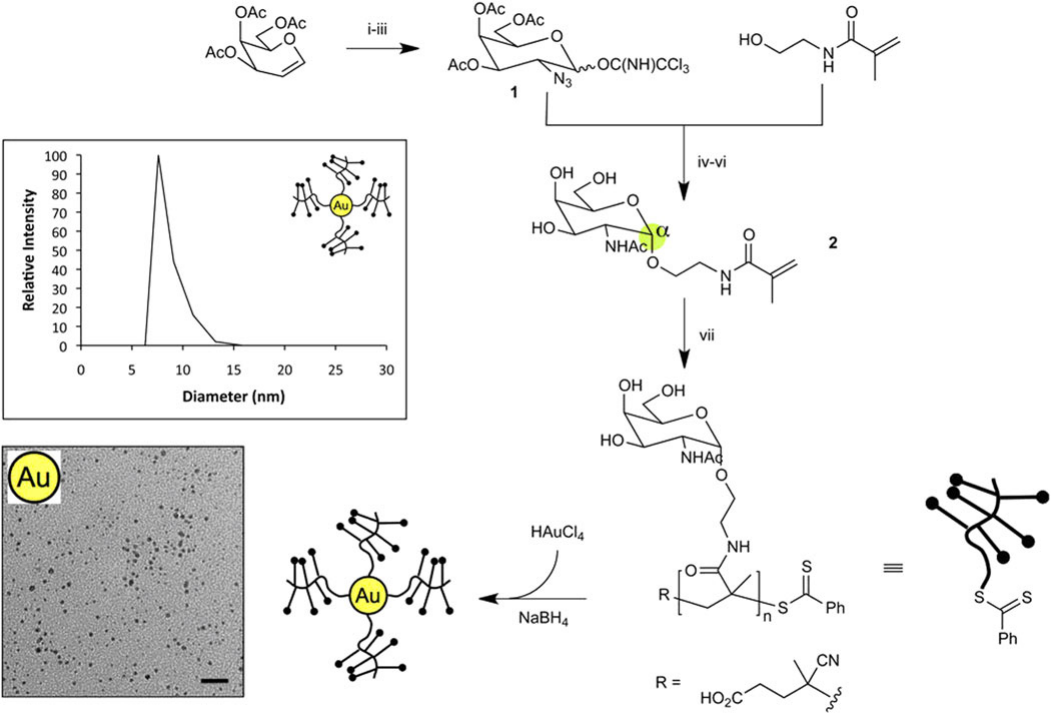
Preparation and characterization of a carbohydrate Tn‐antigen/AuNPs vaccine (Tn = *N*‐α‐acetylgalactosamine linked to serine or threonine). Inset figures show representative dynamic light scattering data (top) and TEM image (bottom) of glycoNPs (scale bar = 20 nm). Reprinted with permission from 223. Copyright 2013 American Chemical Society.

Due to their versatile and reproducible preparation and modification, AuNPs have attracted researchers’ interest as promising therapeutic agents^220^, ^221^ with potential especially in vaccine development.^186^ Although older studies have demonstrated that AuNPs decorated with analogues of viral or tumor cell polysaccharide epitopes were sufficiently immunogenic,^221^ there is only one recent study describing vaccine design based on AuNPs coated with a carrier protein and an LPS from a nonvirulent bacterial strain *Burkholderia thailandensis*.^222^ This glycoconjugate significantly increased the production of LPS‐specific antibodies in nonhuman primates exposed to the virulent bacterial strain *B. mallei*. In animals vaccinated with the AuNP‐LPS‐based conjugate no signs of bacteria were found, while in non‐vaccinated animals the pathogen cells were detected ‐ 102 (in animals that survived the test) and 104 (not survived animals) cfu per mg of tissue. Such results can be considered promising for the development of an efficient protective antiglanders (glanders = contagious and highly fatal disease, which can affect humans) vaccine for humans.^222^ Parry et al. showed that an AuNP‐glycan‐based vaccine efficiently induced the immune response even in the absence of peptide or protein adjuvants.^223^ These authors synthesized polymers displaying glycan units mimicking tumor tissue specific mucin (see Fig. 12). These immunogens were then conjugated in one step with in situ prepared AuNPs. Contrary to unmodified polymers, all glycan conjugates induced an immune response, as evidenced by IgG titres. The authors found out that the optimal number of glycan units per AuNPs‐conjugated polymer chain is 20–25, regardless of the chain length.^223^

**Figure 13:**
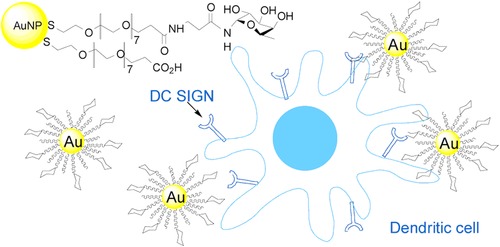
A schematic depiction of fucosylated AuNPs interacting with dendritic cell‐specific intercellular adhesion molecule‐3‐grabbing nonintegrin (DC‐SIGN) receptors. Reprinted with permission from 224. Copyright 2014 American Chemical Society.

Fallarini et al. investigated simple AuNP–glycan conjugates with the aim of understanding the essential components to design an effective vaccine.^225^ In this study, they compared the effect of NPs decorated with nonimmunogenic mono‐ and disaccharides mimicking parts of the capsular polysaccharides of *Neisseria meningitidis* bacterium. The conjugate induced an immune cell response, contrary to nonconjugated forms of carbohydrates, and due to the possible intracellular degradation of the glycoconjugate, a disaccharide‐modified conjugate was more efficient.^225^ Interesting behavior was observed for AuNPs grafted with fucose‐ended linkers. Such AuNP conjugates with a specific glycan surface density were internalized by dendritic cells via their Dendritic Cell‐Specific Intercellular adhesion molecule‐3‐Grabbing Non‐integrin (DC‐SIGN) receptors (see Fig. 13), but without an expected induction of subsequent interleukin production. Observed targeted internalization is a focus for antigen delivery and for the induction of a desired DC‐SIGN‐mediated signaling cascade.^224^

In order to induce an anticancer immune response, a tumor‐specific antigen (a phospholipid‐functionalized glycopeptide) was used for the formation of a scaffold with an iron oxide core.^226^ The highest amount of elicited IgG (mean titres ∼81,402) was confirmed after the vaccination of mice by the conjugate having only one carbohydrate antigen per phospholipid‐modified glycopeptide chain while surprisingly, mean titres of only ∼7530 were observed when two glycosyl units were bound to the chain. It should also be noted that the formation of NPs with an iron oxide core significantly increased the IgG titres for free glycosylated phospholipid‐modified peptide chains from ∼5032 to ∼36,600. Furthermore, a complex induction of the immune response leading to tumor cell degradation was observed,^226^ suggesting that iron oxide NPs can be an alternative to AuNPs for vaccine design. Carbohydrates were used also as adjuvants in AuNP‐based vaccines, with chitosan‐coated AuNPs decorated with plant saponins applied as tetanus toxoid carriers, but the authors did not describe any specific role of chitosan in the fabricated conjugates.^227^

### B. Nonimmunogenic Therapeutic GlycoNPs

In addition to the activation of the immune system with a consequent therapeutic biochemical cascade, a few other therapeutic effects of glycoconjugates were discovered. Such efforts rely on (i) the competitive binding of lectins/glycans on receptors, thus preventing the successful binding of pathogenic viruses/bacteria, and (ii) the ability of glycoconjugates to selectively agglutinate pathogenic particles, thus eliminating their adverse effect. Since the interaction between a carbohydrate‐binding protein and a carbohydrate is not very strong, multivalent recognition entities must be present on a therapeutic NP surface.

There have been numerous reports describing the fabrication of such therapeutics, with the main achievements comprehensively summarized in recent excellent reviews.^90^, ^188^, ^228^, ^229^, ^230^, ^231^ In summary, these papers referred to diverse kinds of multivalent glycoconjugates, that is, based on carbon and metallic NPs, organic supramolecular scaffolds or proteins with many of such conjugates having therapeutic potential. Moreover, novel synthetic and conjugation protocols have been described as well. Interesting conclusions are provided in a review by Jiménez Blanco et al., who suggested that heteroglycoconjugates, compared to homoglycoconjugates, provide additional regulation possibilities, in addition to the already known protein–carbohydrate binding mechanism.^232^ These findings have been considered in recent developments in the design of nonimmunogenic therapeutic glycoconjugates.

#### 1. Metallic NPs

Few recent studies have focused on the antibacterial properties of AuNPs^233^ or AgNPs^234^ coated with 6‐*O*‐chitosan sulfate. Importantly, these glycoconjugates are not only antibacterial due to the presence of Ag or Au but also anticoagulant, making them almost perfect candidates for the surface coating of, for example, medical devices that need to be kept sterile. Similar, although offering only antibacterial properties, AgNPs and AuNPs have been conjugated with other polysaccharides, for example, starch^235^ and aminocellulose.^236^ A similar antibacterial effect has been observed with AgNPs coated with 12‐C‐monosaccharide‐dodecanoic acid^237^ or with MNPs stabilized by poly(ethylene oxide) and functionalized with a sialic acid derivative.^238^ While the aforementioned glycoNPs were tested mainly against *E. coli*, magnetic glycoNPs displaying fucose‐bearing oligosaccharides efficiently block the adhesion of *Helicobacter pylori*.^239^

#### 2. Carbon NPs

**Figure 14:**
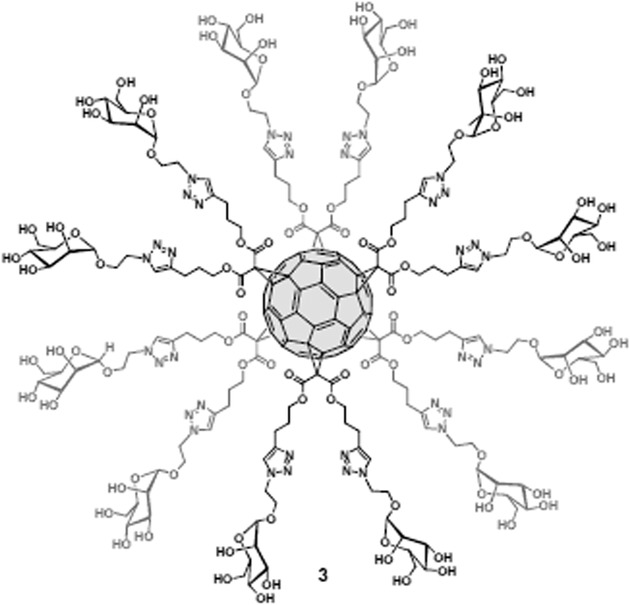
Structure of multivalent mannosylated fullerene used for inhibition of Ebola‐like virus binding. Reprinted with permission from 243. Copyright 2013 American Chemical Society.

Carbon NPs are another choice in the fabrication of glycoconjugates with antiadhesive properties. Recent studies include mannose‐bearing diamond NPs exhibiting an excellent inhibition of *E. coli* type 1 FimH‐mediated adhesion.^240^, ^241^ Ragoussi et al. have described similar mannose modification performed on CNTs and graphene sheets but provided only the results of a selective lectin binding test^242^; the inhibition of bacterial adhesion by such NPs has yet to be assessed. On the other hand, Luczkowiak et al. reported an inhibition of *Ebola* pseudotype virus binding to DC‐SIGN receptor‐displaying cells.^243^ In their study, fullerene with 12 mannoses (Fig. 14) was the most effective virus inhibitor, while increasing mannose valency led to decreased inhibition efficiency,^243^ confirming a need for the controlled synthesis of conjugates with their extensive assessment.

#### 3. Synthetic Polymers

Despite the relative ease of controlled chemical ways of glycosylation of carbon and metallic NPs, polymer scaffolds seem to have been explored the most intensively in this field. One reason may be a wider diversity of synthesized polymeric glycoconjugates available, as shown in the extensive study by Percec et al.^244^

**Figure 15:**
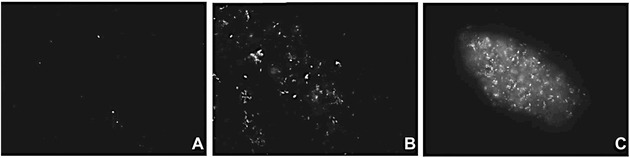
Glycopolymer‐induced agglutination of type 1 fimbriaeted *E. coli* (strain UTI89). (A) Fluorescence microscopy pictures of Katushka‐expressing type 1 fimbriaeted *E. coli* UTI89; (B) exposed to 1 μM of L188; (C) to 3 μM of L188 and resulting in a “bacterial egg” agglomerate with approximate dimensions of 98 × 51 μm. Reprinted with permission from 251. Copyright 2015 American Chemical Society.

Recently reported examples of fully synthesized scaffolds include PAMAM dendrimers displaying carbohydrate Le^x^ moieties capable of competitively blocking DC‐SIGN receptors, thus inhibiting the first phase of HIV infection.^245^ More importantly, the glycodendrimers exhibited a negligible inhibition of langerin, which, contrary to DC‐SIGN, helped to internalize and destroy HIV particles. From the testing of a mini library comprising of third‐, fourth‐, and fifth‐generation glycodendrimers, the latter was found to be approximately twice as efficient in inhibiting the binding of model virus‐like particles compared to the other ones (third and fourth generation). Finally, the conjugate was tested for the inhibition of the real virus influencing DC‐SIGN‐mediated binding and trans‐infection.^245^ Fucosylated, branched phosphodiester‐based scaffolds were reported to bind efficiently to *Burkholderia ambifaria* lectin with a promising application as inhibitors of bacterial infection.^246^ Cholera toxin has been successfully ligated with newly synthesized five‐armed molecular scaffolds, bearing a galactose‐oligomer recognition unit on the end of each arm.^247^ *Pseudomonas aeruginosa* lectin A recognition was suppressed by α‐l‐fucoside and by a β‐d‐galacto‐pyranoside dendrimer hybrid, which may be further exploited for the treatment of *Pseudomonas aeruginosa*‐based infections^248^, ^249^ (for *Pseudomonas aeruginosa* lectin A‐glycan binding AFM images, see^250^). Yan et al. investigated the influence of multivalency of *n*‐heptyl‐α‐d‐mannose on the strength of binding to FimH adhesin displayed on type 1 fimbriae of *E. coli* causing Crohn's disease.^251^ They found that the linear polymer decorated with numerous *n*‐heptyl‐α‐d‐mannose moieties was more efficient in the coagulation of bacteria (Fig. 15) compared to monovalent glycoconjugates or polyvalent star‐like particles.^251^ The potential of multivalent synthetic glycodendrimers was further demonstrated by Ghirardello et al., who reported a 109×106‐fold stronger binding potential (relative potency) to WGA of a 48‐valent GlcNAc dendrimer compared to an unconjugated GlcNAc moiety.^252^ A relative potency of only 1168 was reported for a 30‐valent *N*‐acetyl‐d‐lactosamine (LacNAc) “onion peel” like dendrimer compared to the LacNAc monomer, binding specifically to leguminous lectin from *Erythrina cristagalli*.^253^

In order to protect against eukaryotic parasites, Campo et al. synthesized cyclic triazole‐linked oligomers with pseudo‐glycosidic units with an affinity toward the trans‐sialidase enzyme from *Trypanosoma cruzi*.^254^ As a result, the inhibition of the parasite development after their invasion into macrophage cells was observed, but a quite high concentration (250 μM) of the therapeutic oligomer was needed. Nevertheless, these results are only preliminary, and further optimization of oligomer formation (i.e., a pseudo‐glycosidic unit density and its precise location) is needed to increase the inhibition activity.^254^

#### 4. Biopolymers and Proteins

**Figure 16:**
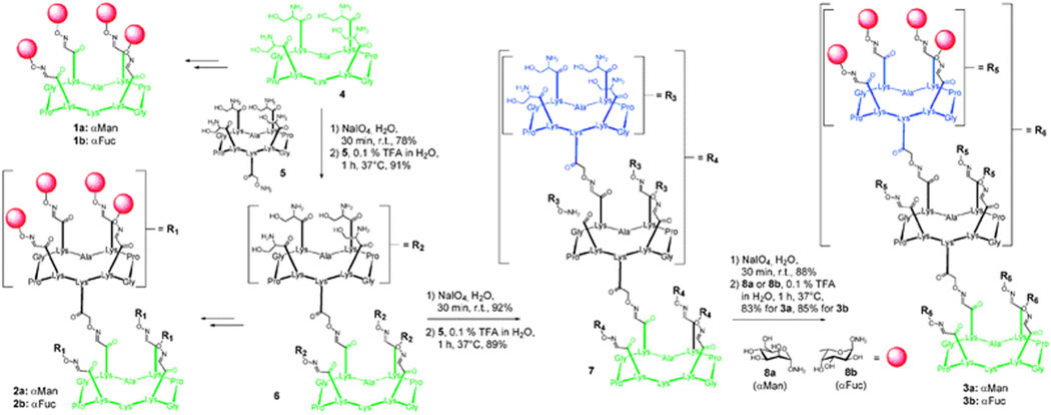
Synthesis and structure of cyclopeptide‐based glycodendrimers. Reproduced from 257 with permission of the Royal Society of Chemistry.

Galactose units displayed on a branched oligopeptide were efficiently bound to the surface lectin of *P. aeruginosa*, inhibiting film formation and disrupting existing biofilms.^255^ Bouckaert et al. explored cyclodextran NPs conjugated with *n*‐heptyl‐α‐d‐mannose using various spacers as potential FimH agonists.^256^ They did not observe improved adhesion to the FimH adhesin of *E. coli*; however, their results suggest that differences in the length of the spacer arm may account for the low undesired affinity for human mannose‐binding lectins.^256^ A further step in the development of antivirotics and antibacterial drugs that are not based on recently used synthetic antibiotics, to which an increased number of bugs are resistant, was described by the preparation of a cyclopeptide‐based dendrimer decorated by glycan moieties using an oxime ligation.^257^ The best‐performing, 64‐valent glycodendrimer from the reported library of compounds (Fig. 16) exhibited a 40,000‐fold increased lectin‐binding potency compared to monovalent methyl α‐l‐fucopyranoside.^257^ Cholera toxin inhibitor with an IC_50_ value of 100 pM based on a tetravalent neoglycoprotein prepared by a simple chemical modification of an inactive B subunit of cholera toxin was reported by Branson et al.^258^ The use of cholera toxin protein as a template secured a “… precise fit of the ligand groups with the spacing and configuration of binding sites on wild‐type CTB.”^258^

Self‐assembled polymeric particles are the third type of organic nanocarriers covered in this section. For example, vesicles self‐assembled from poly(ethylene oxide) and polycaprolactone and further modified with a sialodendrimer block the cellular recognition of influenza virus via ligation with viral hemagglutinins.^259^ Authors have reported a decrease in IC_50_ from 4 mM to 240 μM (per glycan unit) when mono‐ and eight‐valent glycodendrimers were tested, respectively, and a further decrease to approximately 2 μM when the glycodendrimers were conjugated to the polymer vesicles.^259^ Another type of conjugate was prepared in the form of nanodroplets by crosslinking polymer shells that were ready for subsequent covalent modification.^251^ Yan et al. decorated such particles with multiple *n*‐heptyl‐α‐d‐mannose moieties to achieve the efficient coagulation of pathogenic *E. coli* type 1.^251^ *E. coli*’s type 1 fimbrial adhesin (FimH) was also targeted by Wu at al. using mannose‐terminated DNA oligomers.^260^ Complementary DNA strands conjugated with a second‐generation dendrimer were then applied to assemble mannosylated fibers with the capability of agglutinating *E. coli* strain ORN178.^260^ Interestingly, the authors claimed that agglutination ability was independent of mannosyl density on the fiber surface, most likely because of flexible tube‐like NPs.^260^ Yu et al. prepared nanotubes that were self‐assembled from amphiphilic pillar[5]arene, with glucose acting as the hydrophilic part.^261^ These glyco‐nanotubes were able to agglutinate *E. coli* cells more effectively with an increased number of glucose units with the highest agglutination index of 54 (an average number of bacteria connected to each other after successful agglutination).^261^ Therapeutic potential also can be predicted for self‐assembled oligopeptide‐LacNAc conjugates capable of moderating the activity of galectin, a lectin‐type protein with signaling and other properties.^262^

#### 5. GlycoNPs for Enzyme Inhibition and Other Therapeutic Functions

Applications of iminosugars for the treatment of lysosome storage diseases (e.g., Gaucher disease or glycosphingolipid lysosomal storage disorder, characterized by a mutation‐based impairment of different glycosidases with a subsequent pathological accumulation of glycolipids) have been investigated in recent years. Iminosugars are selective and reversible inhibitors of glycosidases due to selective binding to their active sites. This binding can help to fold impaired enzymes, thus partially restoring their hydrolytic activity at the higher pH present in lysosomes. Furthermore, the misfolded enzymes “protected” by the bound iminosugar can avoid degradation otherwise induced by a cellular quality control.^263^ Using this mechanism, iminosugars could be applied for so‐called “pharmacological chaperone therapy,” an emerging way to treat lysosomal storage diseases. The nature of recognition between iminosugar and respective glycosidase differs significantly from that of lectin–glycan binding. This fact raises the question of whether multivalency can increase the inhibition potency of investigated glycomimetics. In fact, previous studies have suggested that multivalent iminosugars lost their inhibition ability, while recent studies using well‐defined and controlled synthesis have reported the opposite.

**Figure 17:**
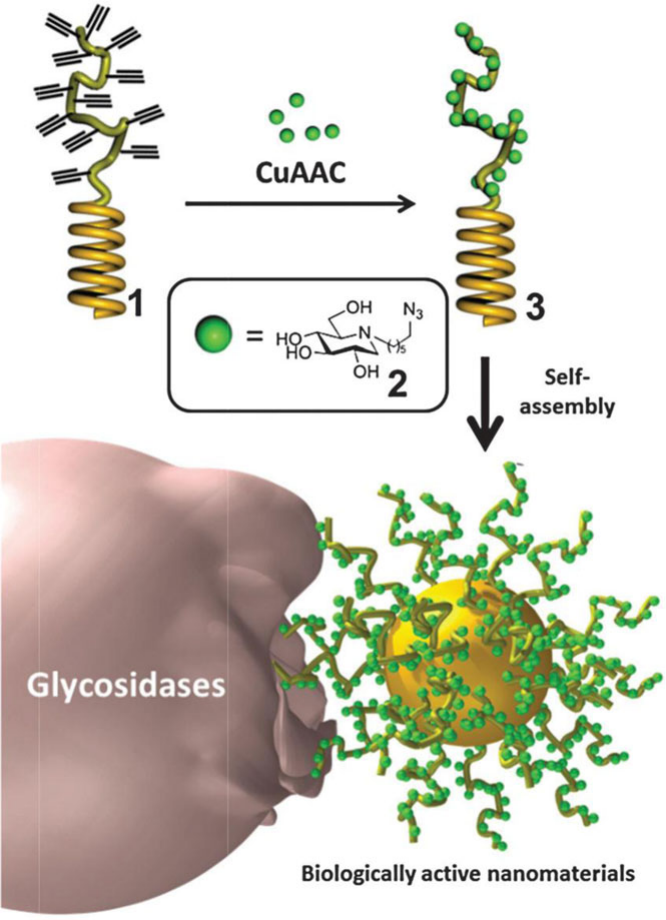
1‐Deoxynojirimycin‐based glycopolypeptides: synthesis, self‐assembly, and glycosidase inhibition model. CuAAC = Cu^I^ catalyzed Huisgen azide‐alkyne cycloaddition. Reproduced from 267 with permission of the Royal Society of Chemistry.

In the study of Garcia Fernandez et al., a mini library of iminosugar‐based glycomimetics (either monovalent or conjugated to fullerenes) was tested for its potency to inhibit a variety of glycosidases.^264^ Their results suggested that in some cases, multivalency can “switch on” the inhibition by switching the binding mode, rather than by the sheer presence of a higher amount of recognition moieties.^264^ In a more recent work, several scaffolds and conjugates displaying deoxynojirimycin, a broad glycosidase inhibitor, were tested.^265^ Interestingly, the strongest inhibitor (800‐fold stronger than a monovalent analogue) was just a tetravalent iminosugar present on a porphyrin‐based scaffold. The authors suggested that the valency itself did not cause an increased inhibition potential, but rather the spatial conformation of monosaccharides did.^265^ Moreno‐Clavio et al. tested several l‐fucosidase iminosugar‐based inhibitors with less success.^266^ Only a sevenfold‐higher inhibition effect on α‐l‐fucosidases was observed when a trivalent scaffold was used compared to a monovalent one.^266^ On the other side, a relative α‐mannosidase inhibition potency as high as 3000 was reported for the *N*‐alkyl analogue of 1‐deoxynojirimycin conjugated on a synthetic glycoprotein scaffold (Fig. 17),^267^ almost 10,000 for the 21‐valent 1‐deoxynojirimycin scaffold^268^ and higher than 500 for glycocyloclopeptide with seven copies of 1‐deoxynojirimycin.^269^ In the latter case, the large inhibition potential was obtained only on glycoconjugates with a longer (C_9_) spacer, while C_6_ spacers of the same glycoconjugate resulted only in a relative inhibition potency of 42.^269^

The real therapeutic effect of different 3‐ to 14‐valent iminosugar conjugates was investigated by Joosten et al. through the in vitro measurement of β‐glucocerebrosidase activities in N370S Gaucher fibroblasts.^270^ The authors reported a “mild, but significant” effect of multivalency. Importantly, the highest inhibition rate was not correlated with the highest chaperoning (therapeutic) effect.^270^ This report clearly suggests that similar systematic investigations will be necessary to develop efficient NP‐based drugs against lysosome storage diseases.

A similar approach applied for the treatment of lysosome storage diseases was also investigated for the treatment of cystic fibrosis. 1‐Deoxynojirimycin and its derivatives correct a misfolded cystic fibrosis transmembrane conductance regulator protein in a similar manner as the aforementioned chaperone effect of iminosugars towards glycosidases, with a 1000‐fold increased inhibition activity of 3‐valent iminosugar compared to a monovalent analogue control.^271^

The concept of pharmacological chaperones may sound very promising, but although the activation of glycosidases has been observed, it hardly reached therapeutically sufficient levels, with typically an approximately twofold increase in the activity.^272^ From this point of view, the work of Brissonnet et al. is noteworthy.^273^ These authors reported an activity increase as high as 70‐fold of a bacterial mannoside‐phosphorylase using cyclodextran‐based NPs displaying deoxymannojirimycin (more than 100‐valent). Nevertheless, further studies are needed to elucidate the nature of the enzyme activation gain.^273^

The selective interaction of a disaccharide called Thomas Friedrich antigen (TF_ag_) with specific tumor cells displaying Gal‐3 lectin had a cytotoxic effect on the targeted cells: a conjugate of small AuPs coated with TF_ag_ via an amino acid linker was synthesized, and an approximately 100‐fold higher cytotoxicity toward the Gal‐3‐positive cells was achieved compared to monomeric units.^274^ The main cytotoxic effect of the TF_ag_ relied on apoptosis induced by an inhibited Gal‐3 signaling,^274^ but it is anticipated that the TF_ag_ targeting may also work well in drug delivery.

Another glycan with a therapeutic effect is heparan sulfate, a glycosaminoglycan‐based polysaccharide capable of inhibiting protease β‐secretase responsible for the accumulation of plaques causing Alzheimer's disease. Nevertheless, carbohydrate synthesis is rather complicated and expensive. Therefore, a mini library of dendritic polymers bearing multiple copies of different heparan sulfate monomers was recently introduced.^275^ Some of these conjugates were found to possess inhibition potential equivalent to that of previously tested oligomers, which is an important result for the future development and production of anti‐Alzheimer's disease drugs.^275^

## 6. CELL/TISSUE TARGETING

While the previous section covered the therapeutic effects of NPs displaying carbohydrate moieties on their surface, there are even more studies investigating glycoconjugates where a carbohydrate moiety plays a different role—targeting specific tissues or cells based on the selectivity of protein–carbohydrate interactions. This selective targeting has been mostly used for the delivery of (noncarbohydrate) drugs, particles, or compounds suitable for thermodynamic therapy or fluorophores/particles, allowing selective tissue/cell imaging using spectrophotometric and other methods (e.g., magnetic resonance). In fact, selective cell imaging is based on efficient selective targeting; thus, in many studies, both aspects are overlapped.

### A. Drug Delivery

From a material point of view, diverse metallic NPs, carbon NPs, and SiNPs conjugated with carbohydrate moieties and drugs to be delivered have been reported in the past. A recent trend (since 2013), however, is the application of polymeric scaffolds or biopolymers, that is, either abundant polysaccharides based on glucose, chitosan, dextrans, or pullulans or protein‐based scaffolds for such purposes.^276^, ^277^, ^278^ Saccharides were applied for the selective internalization of NPs by targeted cells due to receptors displayed on their surfaces with subsequent intracellular drug release. Saccharides were also used to substantially suppress adverse side effects of the treatment by the improved biocompatibility of otherwise hydrophobic, insoluble, or instable substances.

After targeted delivery, drugs must be released from the conjugate to perform their therapeutic effect. Most nanocarriers have been prepared as pH‐sensitive conjugates, taking advantage of different pH values inside many tumor cells, where NP degradation occurs with subsequent drug release. Enzymatic nanocarrier degradation has also been described, as well as the degradation of the conjugate triggered by the presence of specific small molecules (glucose, glutathione, etc.). Irradiation‐sensitive nanocarriers have also been described where on‐demand and “remotely controlled” drug release occurred after near‐infrared (NIR) or UV irradiation.^279^ In addition to a “model” drug (doxorubicin being one of the most frequently loaded cargoes in nanocarrier studies) or therapeutic proteins, carbohydrate‐based nanocarriers have also been used as nonviral vectors.^277^

#### 1. Carbon Nanomaterials

Carbon NPs can be divided into four main groups regarding their dimensionality, that is, 0D fullerenes and spherical NPs, 1D nanotubes and nanofibers, 2D graphene sheets and 3D cryogels, aerogels, or foams. Recently, Li et al. compared of all types of carbon NPs for the simple encapsulation of an enzyme with its subsequent intracellular release.^280^ While three‐dimensional graphene aerogel was suitable for the long‐term release of a cargo (3% in 24 hr), oxidized CNTs and GO provided fast cargo release (15–20% in 4 hr).^280^ Without any doubts, a comparison of different types of NPs is needed for the development of ultraefficient drug carriers. Unfortunately, there is no recent comparative study focusing on the performance of different types of NPs modified with glycans applied in drug delivery.

**Figure 18:**
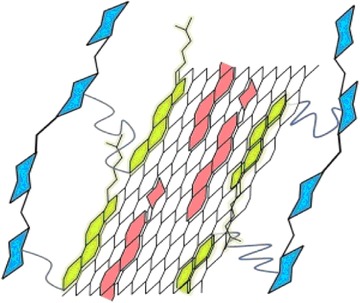
Schematic depiction of reduced graphene oxide (black support) conjugated with cholesteryl (green), doxorubicin (red), and hyaluronic acid (blue) including hydrophobic and π–π stacking interactions. Reprinted from 284. Copyright 2013, with permission from Elsevier.

Many recent studies have explored the potential of graphene‐based NPs, that is, GO^281^, ^282^, ^283^ or rGO.^284^, ^285^ Flakes of the former form of graphene derivative display many oxygen groups, which make GO hydrophilic and easily dispersible in aqueous solutions.^286^ Furthermore, surface‐displayed oxygen moieties can be used for the covalent conjugation of various ligands. Most recent studies have investigated only slightly different variations of a basic scheme relying on GO nanosheets decorated with hyaluronic acid (HA) securing the selective binding of nanocarriers by CD44 receptors present on the surface of various kinds of tumor cells. Song et al. described the improved solubility of doxorubicin adsorbed on GO sheets and the “shielding” of a GO's surface charge by increasing its hydrophobicity.^281^ In this study, a complex of GO‐doxorubicin with modified HA was reported to occur via H‐bonds between HA's amine and GO's epoxide group.^281^ In another study, HA was covalently grafted onto the GO surface via adipic acid dihydrazide.^287^ This GO–HA conjugate was treated with doxorubicin solution, and the drug was adsorbed onto a GO surface, most likely by hydrophobic and π–π stacking interactions.^287^ HA could be attached to a GO surface via introduced sulfhydryl groups, forming disulfide bonds that were cleaved by glutathione inside targeted cells.^288^ A similar approach was tested by Yang et al., who modified the GO surface with carboxymethylated chitosan.^289^ Amine groups present in chitosan were used for covalent linkage to GO's hydroxyls and helped to sequentially attach HA with the subsequent loading of doxorubicin.^289^ All of the authors declared the in vivo inhibition of tumor growth after injection of the prepared conjugates; however, the inhibition rate hardly exceeded 50% compared to the control. Thus, such relatively low efficiency of cancer growth inhibition complicates the further commercial exploration of doxorubicin‐based delivery systems. Interestingly, an almost 80% decrease in the size of epidermal tumors in mice was observed 28 days after the injection of the conjugate prepared from rGO (Fig. 18).^284^ Most probably, higher hydrophobicity of rGO compared to GO allowed the adsorption of a higher amount of doxorubicin. Xu et al. combined the GO‐HA‐doxorubicin delivery system with a photothermal therapy by wrapping AuNPs in GO sheets with the subsequent loading of HA and doxorubicin.^290^ Drug‐induced cytotoxicity based on a nanoconjugate was comparable to a treatment with the same concentration of unconjugated doxorubicin, but upon laser irradiation, the increased temperature of AuNPs decreased the cell viability of hepatoma cell line Huh7 from 44 to 18%.^290^

GO hydrogel modified with heparin‐mimicking sodium styrene sulfonate was perfectly biocompatible with blood. Such a nanoconjugate could have a broad range of therapeutic applications, including drug delivery.^291^ Nevertheless, only a nonselective antimicrobial activity of particles loaded with gentamycin sulfate was tested.^291^

CNTs offer similar surface properties as graphene‐based NPs, and their complexes with HA were also tested for doxorubicin delivery. However, when the drug was complexed with CNTs covalently grafted with PEI and HA, an in vitro study revealed a quite similar viability (approx. 50%) of model tumor cells treated with conjugated or unconjugated doxorubicin (24 hr, ∼4 μM doxorubicin).^292^ More promising results were obtained with covalently grafted polyethylene glycol derivative to CNTs, crosslinked to a gel form by adding β‐cyclodextrin and treated with camptothecin (a hydrophobic cytotoxic drug).^293^ An in vitro study showed the significant growth inhibition of a cancer cell line (∼80% inhibition after 7 days compared to cells treated with blank CNT‐polymer gel). The gelation process relayed on the natural ability of cyclodextrins to include certain molecules (PEG moieties, in this case) into their cavity via host–guest interaction. Such an absorption capacity also has great potential for hydrophobic drug loading. Furthermore, the feasibility of precise chemical modification makes cyclodextrins a great tool not only in smart drug development but generally in the molecular fabrication of diverse mechanisms.^293^ These possibilities have been reviewed, for example, by Martínez et al.^294^

In addition to carbon, boron nitride nanotubes have also been exploited for drug delivery. Such nanotubes with covalently grafted glycosylated chitosan exhibited low cytotoxicity; therefore, such NPs could be used as nonviral vectors.^295^ A plasmid gene was more efficiently expressed in human carcinoma epithelial cells (A549) than in control cell lines even though these NPs did not exhibit any specific receptor ligand selectivity.^295^ Enhanced plasmid expression could be assigned to more efficient endocytosis rather than to the presence of specific receptors in A549 cells.^295^

An intriguing approach was reported by Zhou et al. by loading doxorubicin into pores of mesoporous carbon NPs.^296^ These NPs were subsequently grafted by HA, which guided selective drug targeting. Furthermore, HA acted as a “gatekeeper” because it was specifically recognized and degraded by hyaluronidase‐1 after internalization and allowed drug release. In addition to enzymatic degradation, HA coating could also be removed by glutathione due to the nature of a covalent bond between HA and NPs, making drug release more efficient and controllable. The authors reported an IC_50_ of 8.9 μM for doxorubicin carried by the nanocarrier.^296^

#### 2. Metal and Metal Oxide NP‐Glycan Conjugates for Drug Delivery

AgNPs able to generate oxidative stress have strong antimicrobial properties. Their modification with glycans represents very simple carbohydrate‐guided therapy applicable in bactericidal surface fabrication (vide supra) and possibly also in cancer treatment. Kennedy et al. revealed that AgNPs modified with a monosaccharide galactose were internalized by model neural and cancer cells more efficiently than mannose and glucose conjugates, but interestingly, the former exhibited lower cell toxicity (approx. fourfold lower EC_50_ against model cancer cells) compared to less‐internalized glycoconjugates, suggesting an important role of terminal saccharide in glycan moieties.^297^ Similarly, Shahbazi et al. observed a significant level of cytotoxicity (approx. 80%) when the HT1080 sarcoma cell line was treated with iron oxide NPs (100 or 200 nm in diameter) coated with glucose, but a similar effect was observed with NPs coated by polyethylene glycol, suggesting the low targeting capability of such conjugates.^298^

Apparently, AuNPs have not been considered preferential drug carriers, as discussed in a previous section, despite the fact that there are studies reporting the simple decoration of AuNPs with carbohydrate‐based targeting moieties (mannose modified^108^) and even loaded with therapeutic agents. The latter was achieved by the conjugation of moieties capable of enhanced drug ligation to AuNPs, with cyclodextrins being very sound examples of such approach. Cyclodextrins (and their derivatives^299^) can host hydrophobic, insoluble, drugs inside their structure^294^ and can be easily conjugated to AuNPs.^300^ Alternatively, AuNPs can be conjugated directly with cytotoxic ligands, for example, gold(I) triphenylphosphine.^301^ On the other side, similar to the above‐mentioned carbon NP‐based nanocarriers, HA derivative coated on gold nanocages was used as a vehicle for the targeted delivery of doxorubicin into CD44 receptor‐expressing cells.^302^ The intracellular hyaluronase‐1‐induced degradation of the conjugate resulted in drug release and selective cytotoxicity. In contrast to carbon NPs, gold nanocages could also be applied for thermodynamic therapy. This synergy of cancer cell treatment led to the complete elimination of a solid tumor in mice in 9 days by combined photo‐ and chemotherapy.^302^

Terpen (borneol) stabilized selenium NPs decorated with glucose were also reported to deliver an anticancer drug selectively into Hep2 cells (a hepatocarcinomic cell line).^303^ In addition to an efficient pH‐sensitive system of drug release from the conjugate, an intracellular, selenium NP‐directed production of reactive oxygen species (ROS) was also observed, making the fabricated glycoconjugate cytotoxic for drug resistant hepatocarcinomic cells.^303^ Similarly, TiO_2_ NPs coated with HA and loaded with cisplatin containing ligand were found to carry their payload selectively into an ovarian cell line (A2780) with pH‐induced drug release from these glycoconjugates.^304^ Nevertheless, only an approximately twofold increased drug‐induced cytotoxicity against model cancer cells was observed upon treatment with conjugated versus unconjugated drug (a decrease to approx. 40 vs. 70% of the original viability, respectively, using 10 μM drug).^304^

Polysaccharide carboxymethyl dextran was also used as a building block for the synthesis of magnetoliposomes loaded with doxorubicin.^305^ Drug release was enhanced upon the exposure of the conjugates to a low‐frequency magnetic field in synergy with low pH induced structural changes leading to drug release. In vitro experiments confirmed the low cytotoxicity of the loaded magnetoliposomes to brain cells, while after incubation with 2.5 μg mL^−1^ of conjugated and free doxorubicin equivalents, the viability decreased to approx. 40 and 70% of original viability, respectively.^305^

#### 3. Silica‐Based Glycan‐Targeted Drug Carrier Systems

In a rather “traditional” approach, small SiNPs were covalently modified with spacers; each one ended with trivalent glucose as a targeting unit.^306^ The spacers provided a space for the inclusion of molecules of the anticancer drug paclitaxel. The drug delivery system was the same as described before, that is, whole conjugates were selectively internalized by cancer cell line HepG2, and the drug was released either by intracellular esterases or by low pH. Due to the good pharmacokinetics of the fabricated conjugate, a much higher toxicity (IC_50_ = 0.7 μM paclitaxel equivalent) was induced than with a treatment by the unconjugated drug upon prolonged incubation (no viability change using 0.7 μM paclitaxel).^306^

**Figure 19:**
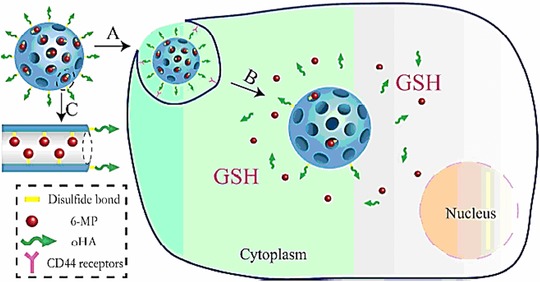
Schematic depiction of glutathione (GSH) induced drug release from hyaluronic acid (HA) capped pores of silica NPs. These pores are filled with disulfide‐bond tethered 6‐mercaptopurine (6‐MP) and, due to HA‐decoration, nanoparticles are captured and internalized via so called CD‐44 receptors—a common surface glycoprotein with significantly higher concentrations on the surface of certain cancer cells. Reprinted with permission from 309. Copyright 2014 American Chemical Society.

Polysaccharide moieties were also used to increase the biocompatibility of a mesoporous silica doxorubicin carrier.^307^ Yu et al. adapted an already developed protocol to prepare HA‐coated mesoporous SiNPs transporting doxorubicin selectively into human colon cancer cells.^308^ A decrease of targeted cell viability by approximately 50 and 20%, respectively, was observed upon incubation with 0.25 μM drug equivalent carried by HA‐coated NPs and uncoated NPs or free drug.^308^ HA‐targeting mesoporous SiNPs with both a therapeutic agent and HA attached to NP's surface by disulfide bonds were investigated by Zhao et al.^309^ Disulfide bonds were cleaved by glutathione, causing efficient drug release (Fig. 19). Cytotoxicity assessment showed that after 72 hr of treatment with the drug‐loaded conjugate, a cell line with CD44 receptors retained a viability of 39%, while the viability of a control cell line without CD44 was 49%, suggesting rather strong nonspecific internalization by the NIH 3T3 cell line.^309^ The same group also prepared mesoporous SiNPs with pores loaded with doxorubicin and capped by HA attached to the surface by disulfide bonds.^310^ Excess glutathione and hyaluronidases helped to release capping molecules from the conjugate.^310^ An elegant approach using nonspecific internalization based on differences between the metabolic activity of normal and cancer cells was reported.^311^ Cancer cells exhibit increased glucose uptake with the enhanced uptake of synthesized glucose‐modified SiNPs. Celastrol used as an anticancer drug negatively affects the mitotic cycle, which is faster in cancer than in normal cells, thus making cancer cells more susceptible to the effect of the drug. Mesoporous SiNPs decorated with glucose via dendritic polymer linkers were found to induce the apoptosis of HeLa cells (50% apoptotic cells after 24 hr of incubation with 5.3 μM drug equivalent). On the other hand, a lower exposure time induced the production of heat shock proteins, suggesting that a subcytotoxic dosage (or exposure time) may even help to protect normal cells.^311^

Carbohydrate modification can also be used for the selective and triggered drug release from mesoporous magnetic SiNPs.^312^ Lectin‐like phenylboronic acid (PBA) displayed on their surface anchored dextran, which efficiently gated drug‐loaded pores in the body of magnetic SiNPs. Once glucose is present in sufficiently high concentration, it competitively binds to PBA, and the dextran “gatekeeper” layer is consequently released from the surface. Due to the much smaller size of glucose compared to dextran chains, drug molecules can easily migrate from the pores. This glucose‐dependent release has great potential, for example, in diabetes treatment, even though a selective cytotoxicity against HeLa cells in the presence of glucose was demonstrated in this study (60% decrease of viability compared to a 25% decrease upon treatment of the cells with unloaded and drug‐loaded NPs without dextran, respectively).^312^ Employing the same gate‐keeping approach, Zhou et al. bound mannose units on the surface of mesoporous SiNPs via disulfide bonds.^313^ These moieties tethered Con A lectin, a molecule large enough to prevent the migration of doxorubicin from the pores of mesoporous SiNPs. Selective release was triggered by the elevated concentration of glutathione in the cytoplasm of cancer cells, and the conjugate exhibited an IC_50_ of 25 μg mL^−1^ against cancer cells, while a negligible viability change was observed when the conjugate was incubated with normal cells having a lower cytoplasmic glutathione concentration.^313^

Chondroitin sulfate is another natural polysaccharide with the ability to promote the uptake of conjugates by selective interaction with CD44 receptors. Moreover, such a biopolymer has the ability to improve the biocompatibility and stability of NPs. All of these features of chondroitin sulfate were used to fabricate a mesoporous silica‐based vector loaded with a plasmid carrying the gene for the p53 protein.^314^ A more than twofold increase in translated messenger RNA for p53 was observed in treated cancer cell lines, when the silica‐based vector was coated with chondroitin sulfate compared to bare mesoporous silica–plasmid conjugate.^314^ Gene therapy induced expression of healthy p53 protein in human cancer cells inhibits tumor growth; thus, the above‐described vector represents another possible way for cancer treatment.

#### 4. Polymer Scaffold Based Drug Carriers

Liposomes prepared from a synthetic building block containing a hydrophobic tail conjugated with galactose as targeting units were capable of transporting doxorubicin easily into HepG2 cells displaying asialoglycoprotein^315^, ^316^, ^317^ and other receptors.^44^ A very similar system, only with incorporated disulfide bonds in the structure of micelles, was reported.^318^, ^319^, ^320^, ^321^ Such glycoconjugates were selectively internalized, and a drug was released after the glutathione‐mediated cleavage of S–S bonds.^318^, ^319^, ^320^, ^321^ Although these studies demonstrated the selective cytotoxicity toward targeted cancer cells in vitro, only Zhao et al. have reported the in vivo inhibition of tumor growth after the localized injection of galactosylated, doxorubicin‐loaded liposomes (tumor weight of approx. 50% compared to animals treated with an unconjugated drug).^316^ The unfolding of synthetic^322^ or natural^323^ micelles triggered by UV irradiation for drug release was also reported, but such an approach was not tested for in vitro or in vivo cytotoxicity. In order to better control the form and chemical properties of nanocarriers, a tri‐block copolymer folded into different types of mannosylated micelles was synthesized.^324^ Preliminary results suggested that there was some correlation between mannose receptor‐induced internalization and the shape of a micelle, but in vivo or in vitro studies investigating the inhibition of virus binding have yet to be conducted.^324^

To discuss drug delivery from an application point of view, it is worth mentioning the use of a synthetic copolymer folded into micelles tested for the oral administration of sorafenib—the drug applied mainly for the treatment of kidney and liver cancers.^325^ In vivo studies have demonstrated that galactosylated, drug‐loaded micelles efficiently and selectively administered sorafenib into the liver and secured its continuous release for several hours.^325^ To improve the oral administration of darunavir, self‐assembled micelles made from synthetic copolymer poly(ethylene oxide)‐poly(propylene oxide) were glycosylated by microwave‐assisted ring opening reaction and loaded with the drug by a simple incubation of the two components in solution.^326^ An approximately 500‐fold higher solubility of darunavir was observed upon its integration into micelles. This is a significant step in the development of less expensive anti‐HIV drugs, considering the fact that the low administration efficiency of present darunavir‐based delivery increases its cost.^326^ Berberine hydrochloride is another drug with a reported anticancer effect with chemical properties not allowing oral administration.^327^ Drug encapsulation into chitosan‐coated phospholipid micelles significantly improved pharmacokinetics and distribution parameters due to the increased biocompatibility of the conjugate. It should be noted, however, that only the biodistribution and drug release profile were assessed in the study, with results suggesting that chitosan‐coated liposomes are good vehicles for the oral administration of berberin.^327^ Micelles prepared by the conjugation of α‐tocopherol (recognized as a potential anticancer drug) with pullulan were reported to release the drug 10‐hydroxycamptothecin at the low pH typically present in the cytoplasm of tumor cells.^328^ In vitro cell assays revealed an approximately 60% decreased viability of targeted cells compared to a control experiment with another cell line.^328^ A conjugate based on α‐tocopherol derivatives together with HA and cytostatic docetaxel in the micellar form was found to induce cytotoxicity and to decrease multidrug resistance via the α‐tocopherol‐mediated inhibition of the active drug transport from the cell.^329^ The suppression of drug resistance on a molecular level was also achieved by the delivery of two recently synthesized inhibitors of efflux proteins (P‐glycoprotein and breast cancer resistance protein) loaded in poly‐lactic‐co‐glycolic‐acid.^330^ A higher inhibition level of cancer cells incubated with conjugated compared to unconjugated inhibitors was demonstrated.^330^ Interestingly, the same efflux mechanism was also suppressed by hydroxypropyl‐β‐cyclodextrin moieties grafted onto synthetic copolymer NPs, which enhance the effect of low‐soluble immunosuppressor tacrolimus by oral administration.^299^

**Figure 20:**
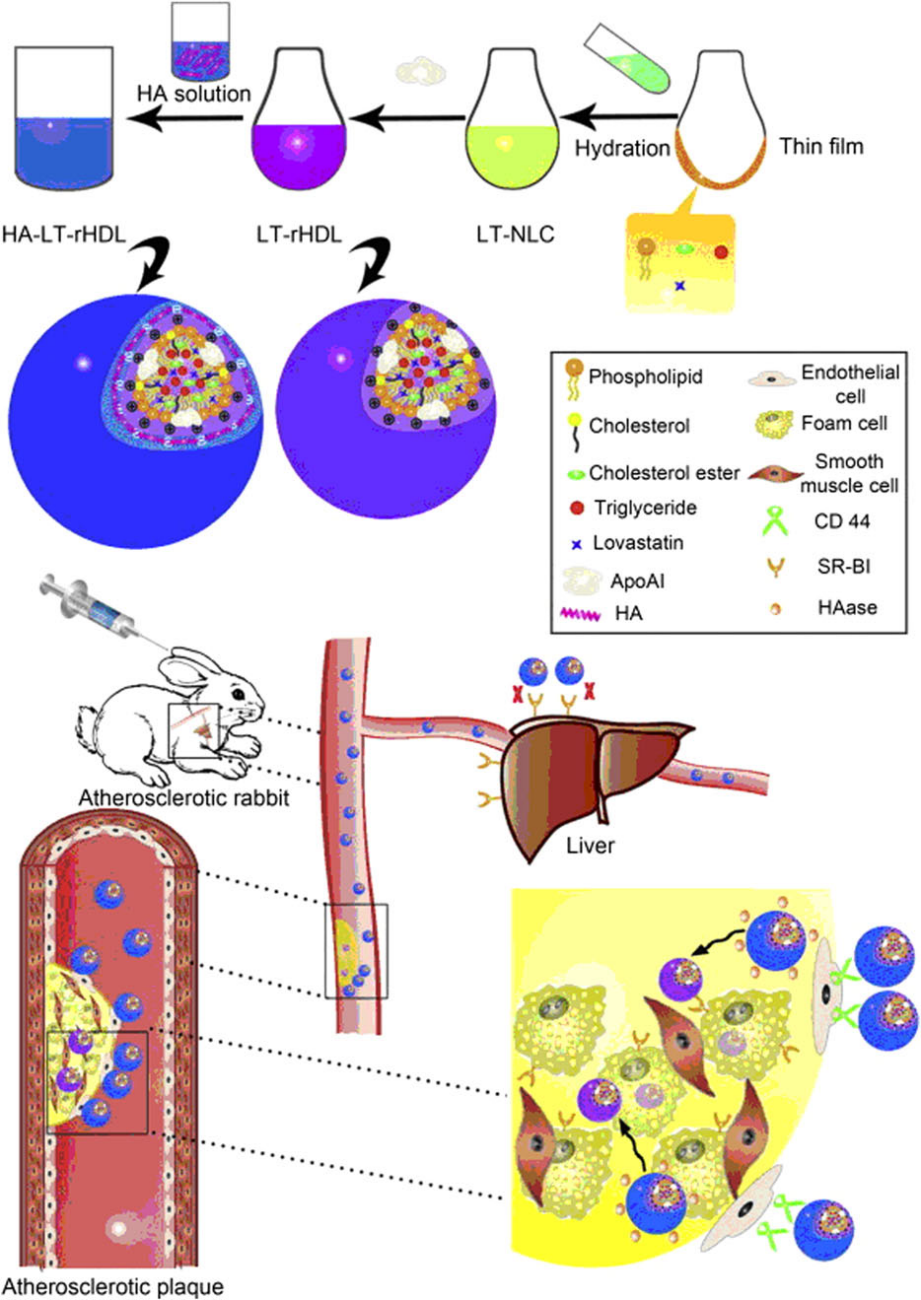
Scheme of fabrication and function of dual‐targeted reconstituted liposomes. Upper scheme represents preparation of micelles containing levostatin and coated with HA (HA‐LT‐rHDL). After injection in an atherosclerotic rabbit, HA coating allowed preferential distribution of HA‐LT‐rHDL into veins and their retention in an organism with decreased rate of phagocytosis in liver. Once HA‐LT‐rHDL reached a lesion site, it was recognized by CD44 receptors on the surface of endothelial cells which helped their transportation inside an atherosclerotic plague (the scheme in the lower part of the figure). In the plague, hyaluronidases removed HA from HA‐LT‐rHDL and the resulting LT‐rHDLs were preferentially recognized by SR‐BI receptors on surface of “foam cells” where lovastatin was intended to be released. Reprinted from 331. Copyright 2014, with permission from Elsevier.

Atherosclerosis can be efficiently treated by the internalization of micelles prepared from a reconstituted high‐density lipoprotein (rHDL) loaded with levostatin mediated via Scavenger Receptor class B member 1 (SR‐BI) receptors accessible on the surface of cells present in a macrophage‐infiltrated atherosclerosis lesion.^331^ Upon coating of the fabricated micelles with HA (Fig. 20), their administration became more efficient and precisely targeted in two steps: (i) because of their lower accumulation in the liver, this is achieved because HA‐decorated particles are not recognized by SR‐BI receptors and “scavenged” (recognition by SR‐BI receptors in liver cells leads to an increased level of phagocytosis). Thus, more particles are delivered into atherosclerotic veins. In the second stage, (ii) HA was readily recognized by CD44 receptors present in atherosclerotic plagues, where HA‐LT‐rHDL was accumulated. Next, due to the presence of hyaluronidases, HA was removed. The formed LT‐rHDL NPs were recognized by SR‐BI receptors of atherosclerosis lesion cells, which delivered them into desired “foam cells” where levostatin was released.^331^

The antiseptic drug NK007 was delivered successfully by oral application to regress murine colitis.^332^ Polysaccharide components of micelles are responsible for efficient target delivery as could be concluded from the in vivo study. Nevertheless, a significant difference between the parameters recorded to evaluate treatment efficacy (i.e., colon length, stool consistency, etc.) was only observed between the control and treated groups but not between groups treated with unconjugated or NP‐conjugated NK007.^332^

Amphotericin B, an antiparasitic drug effective, for example, against *Leishmania donovani*, induces several harmful side effects, with nephrotoxicity and hemolytic toxicity being the most severe.^333^ Moreover, the drug is relatively expensive. Khan et al. conjugated this drug with mannosylated synthetic dendrimer to gain biocompatibility. As a result, no kidney damage or hemolysis was observed with the antiparasitic activity of the drug preserved. In fact, due to the mannose‐driven targeting of conjugates into macrophages, IC_50_ (39 nM) decreased approximately fivefold compared to the unconjugated drug or its commercial analogue.^333^

A supramolecular scaffold prepared from a polymer bearing PBA and a “complementary” glycopolymer was degraded under hyperglycaemic conditions.^334^ The conjugate could be applied for the safe, comfortable, and glucose concentration‐sensitive distribution of insulin.^335^ Zheng et al. also employed a PBA‐based conjugate with both PBA and lactobionic acid moieties displayed randomly on each copolymer chain.^334^ Such molecules folded into a scaffold able to encapsulate insulin. PBA enhanced the mucoadhesion of the scaffolds, which could be then used for the nasal administration of insulin. Therapeutic effect was confirmed by in vivo experiments in diabetic mice with glucose levels decreased to approximately 40% 8 hr after the addition of therapeutic NPs, while such a decrease was observed within 0.5 hr when bare insulin was injected.^334^

The single‐step ligation of HA with doxorubicin seems to be much simpler, forming conjugates that are selectively cytotoxic to cancer cells displaying CD44 receptors^336^ similar to pullulan‐doxorubicin micelles selectively targeting cells with asialoglycoprotein receptors.^337^, ^338^ A similar weight reduction of induced tumor was observed in vivo after treatment with unconjugated and conjugated doxorubicin (approximately 0.4 g of tumor weight compared to 1.8 g developed in a control group), but the life span was longer and the animal weight was larger for NP‐treated mice compared to mice treated with unconjugated doxorubicin, suggesting the significant suppression of a negative side effect of a chemotherapy.^338^ Plant glucomannan covalently conjugated with an inhibitor (bisphosphonate alendronate) of tumor‐associated macrophage was also prepared as a conjugate inducing selective, mannose receptor‐mediated macrophage apoptosis (a tumor weight of approx. 0.3 g was observed 14 days after the application of therapeutic NPs compared to 1.5 g developed in an untreated group).^339^ Mannose receptors of macrophages were also targeted with ionic polymer‐based micelles containing the model protein to be delivered (BSA).^340^ Upon coating of the micelles with β‐glucan from yeast cell walls, NPs were selectively internalized by macrophages.^340^ So‐called “ivy NPs” are nature‐derived nanocarriers consisting of a heavily glycosylated protein core.^341^ When loaded with doxorubicin, a targeted delivery and release of a drug into different cancer cell lines was observed.^341^ The treatment of a solid tumor was equally efficient for the application of both the conjugate and unconjugated doxorubicin, but with a significantly lower weight loss of the animal observed after the application of the conjugate.^341^

Quite a “minimalistic” approach was reported, relying on short polyethylene glycol chains decorated with several saccharidic units along their length and with a bioactive molecule on the chain end.^342^ Such conjugates bearing two separated mannosyl units (the optimum distance between these units was found to be 5.6 nm) were internalized by macrophage cells via their mannose‐binding receptors. The authors declared a good biocompatibility of the carriers as demonstrated by the absence of lysosome storage disease‐like effect after their application.^342^ On the other side, in vivo tests of applicability of such conjugates have yet to be performed. Similarly, Thomas et al. reported micelles prepared from bis‐l‐galactose lysine.^343^ Galactose displayed on the surface of these NPs was enzymatically oxidized, and the aldehydes formed were used for the covalent crosslinking of more NPs together by di‐ or trivalent amine. The authors tested their delivery system only for the complexation of AuNPs, but selective delivery of drugs is anticipated.^343^

A selective drug delivery into the central nervous system, passing the blood–brain barrier, is always very difficult. Recently, certain lectins displayed on NPs helped to enhance such crossing.^344^ For example, polyethylene glycol based micelles decorated with lectin *Solanum tuberosum* were able to successfully deliver a basic fibroblast growth factor via the nasal route into the brain in order to improve the cognition of those affected by Alzheimer's disease.^344^ Lactoferrin, an iron‐binding glycoprotein, was found to be helpful in delivering drug‐loaded micelles through the blood–brain barrier^345^, and the same protein was used to target asialoglycoprotein receptors on HepG2 cancer cells, as well.^346^

Another way to pass the blood–brain barrier is using activated membrane transporters. Glucose transporter 1 was activated by p‐aminophenyl‐β‐d‐manno‐pyranoside with a subsequent transportation of anticancer drugs into the brain cells.^347^ Kuo et al. prepared NPs based on solid lipid decorated with p‐aminophenyl‐β‐d‐manno‐pyranoside and folic acid and loaded with etoposide (a drug inhibiting the proliferation of malignant glioblastoma) that were efficiently distributed through the blood–brain barrier with efficient secondary targeting to glioblastoma cells secured by folic acid in in vitro experiments.^347^

Nonviral gene transport is just another field where the aforementioned techniques of controlled targeted drug delivery have been employed. One of the materials used quite often for the preparation of nonviral vectors is PEI, which can be effectively complexed with the DNA or RNA of interest, but it possesses a certain level of toxicity and its targeting is not selective and thus suitable only for in vitro experiments. In order to apply PEI‐based conjugates for in vivo applications, several ways to improve the performance of such carriers were sought. For example, PEI was complexed with a biocompatible anionic glucose‐based glycopolymer,^348^ GlcNAc,^349^ chondroitin sulfate,^350^ HA,^351^ or depolymerized guar gum with available mannose units^352^ with subsequent use in selective targeting. Alternatively, copolymers of the methacrylamide backbone decorated with glucose,^353^ α‐d‐mannopyranosyl,^354^ or trehalose^355^ and cationic moieties could electrostatically complex plasmids with the subsequent selective transport of plasmid DNA into cancer cells^353^, ^354^ or siRNA into glioblastoma cells via glucose transporter‐1.^355^ Furthermore, complexed siRNA was reported to retain its biological activity after freeze drying.^355^ An electrostatic complexation of DNA with copolymer chains bearing a targeting GlcNAc moiety was also reported.^356^

Targeted gene delivery was also achieved by DNA incorporation into PEGylated micelles decomposing at low pH and decorated with galactose^357^ or mannose^358^ to target asialoglycoprotein or mannose receptors, respectively. Noteworthy, a secondary target ligand was co‐entrapped with DNA to help penetrate through the nuclear membrane.^357^ Mannose‐decorated cholesteryl‐based synthetic liposomes with virus‐like characteristics were reported to effectively transfect nonactivated dendritic cells by a plasmid‐containing gene for luciferase.^359^ Mannosylated chitosan was also used for nonviral vector preparation^360^ as well as mannosylated “bubble lipoplexes,” that is, NPs with ultrasound‐triggered DNA release causing the transfection of M2 macrophages and their switch from tumor growth‐promoting into tumoricidal M1‐like macrophages.^361^, ^362^ In vivo experiments confirmed that this treatment could inhibit the growth of various tumors without adverse side effects.^361^, ^362^ A triblock copolymer consisting of hydrophobic and pH‐sensitive parts to assure the formation of micelles, polycation complexing oligonucleotides, and a mannose‐modified part was also applied to target M2 macrophages.^363^ Synthetic vectors based on siRNA‐PEGylated cyclodextrin scaffolds with a peptide ligand as a targeting unit were also investigated.^364^

**Figure 21:**
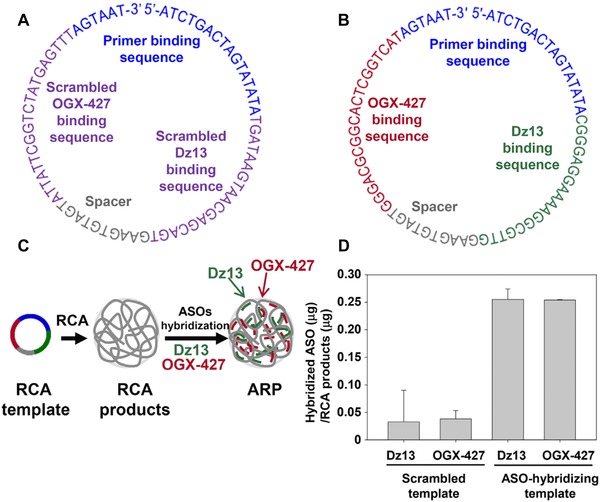
RCA template and hybridization efficiency. (A) Secondary structure of scrambled rolling circle amplification (RCA) template. (B) Secondary structure of a dual antisense oligonucleotide (ASO) hybridizing RCA template for Dz‐13 and OGX‐427. (C) RCA products with poly ASO‐binding sequences were hybridized with two therapeutic ASOs, Dz‐13 and OGX‐427, to produce dual ASO‐hybridizing RCA products. (D) The hybridization efficiencies of the ASOs were tested for products of scrambled RCA templates and dual ASO‐hybridizing RCA templates using fluorescently labeled ASOs. Reprinted from 365. Copyright 2015, with permission from Elsevier.

In addition to the broadly used electrostatic interactions used for loading nucleotides into delivery NPs, Kim et al. employed hybridization with a specific DNA template with a condensation of the resulted DNA complex by a viral Mu peptide (Fig. 21).^365^ The formed “DNA nanoballs” coated with HA were effectively internalized by cancer cells. In vivo experiments showed that antisense oligonucleotides were hybridized with their complementary mRNA in the cytoplasm, leading to a significant sensitization of the cells to doxorubicin treatment and to a significant decrease of tumor growth in mice.^365^

A very simple approach relying only on the application of bare therapeutic oligonucleotides conjugated with a targeting ligand (e.g., a triantennary GalNAc^366^, ^367^, ^368^ or glucose^369^) was also tested. These agents were capable of blocking the adverse immunostimulation of an anti‐HIV oligonucleotide. This may be helpful in the fabrication of anti‐HIV drugs without adverse side effects. The conjugate can be easily prepared, but there is a concern regarding the stability of the carrier when exposed to endo‐ and exonucleases.

### B. Photothermal and Photodynamic Therapy

Photothermal and photodynamic therapeutic methods rely on the targeted delivery of entities capable of strong energy absorption in the NIR spectrum. As a result, the NIR‐triggered increase of local temperature damages targeted (cancer) cells. Traditionally, metallic NPs have been used as photothermal agents, with AuNPs being the first choice. For example, HA‐covered gold nanostars very efficiently inhibit HeLa tumor growth in mice with almost complete tumor disappearance within 7 days after injection of the conjugate and laser irradiation.^370^ The recent trend, however, is the use of a combination of simple photothermal therapy with other treatments, for example, chemotherapy. AuNRs wrapped in GO sheets conjugated with doxorubicin were reported to be fourfold‐stronger inducers of HeLa cell death compared to the same conjugates without doxorubicin used for sole photothermal treatment.^290^

In addition to AuNPs, carbon mesoporous NPs were also employed simultaneously as carriers and photodynamic agents.^296^ A synergic chemotherapeutic and photothermal effect was observed after loading their pores with doxorubicin and final coating with HA. The observed IC_50_ against the MDA‐MB‐231 cell line decreased from 8.7 to 2.3 μM doxorubicin after simultaneous treatment with the drug‐loaded NPs and NIR laser.^296^

A very simple but effective system was reported based on the electrostatic integration of HA and polyaniline applied as a targeting and photosensitizing component, respectively.^371^ The use of these NPs with subsequent NIR laser treatment led to the complete degradation of induced tumors in mice approximately 8 days after treatment without any observable body weight loss.^371^ However, the ternary design of multimodal therapeutic NPs was presented, containing poly(lactic acid) NPs coated with derivatized chitosan to increase the biocompatibility of the conjugate and IR 820 (a photosensitive dye).^372^ Interestingly, two absorption peaks were found for IR 820 encapsulated in the polymer; thus, irradiation with two different wavelengths increased the photothermal effect with the decreased viability of the targeted breast cancer cell line by 70%.^372^

In another treatment design called photodynamic therapy, photosensitizers were used to generate cytotoxic ROS upon the absorption of irradiation with a specific wavelength. Recently, AuNPs coated with HA (a targeting component) and a porphyrin‐based photosensitizer were reported to decrease the viability of cancer cells with CD44 receptors down to approximately 15% upon irradiation with UV light.^373^

AuNPs can also exhibit a photosensitizing effect, but for the generation of cytotoxic radicals, X‐ray irradiation is required. In this sense, glucose‐decorated AuNPs were tested in vitro as selective sensitizers for radiotherapy, leading to increased cancer cell death by 20% compared to radiotherapy with unconjugated AuNPs.^374^

Organic carriers for ROS‐generating photosensitizers were also reported. For example, porphyrin‐derived photosensitizer conjugated to glucose‐decorated poly(methacrylamide) polymer chains^375^ or encapsulated into poly(lactide‐co‐glycolide)‐based NPs coated with HA.^376^ In addition to porphyrin‐based photosensitizer, docetaxel was also co‐entrapped to induce a synergistic photochemotherapeutic effect with IC_50_ of 8 ng mL^−1^, while an IC_50_ of 160 ng mL^−1^ was observed upon treatment with an unconjugated drug under the same conditions.^376^

Fu et al. used chitosan‐coated SiNPs loaded with benzoyl peroxide as photosensitizers.^377^ A cytotoxic test against human breast carcinoma cell line ZR75‐30 revealed that chitosan coating did not substantially affect cell viability (70 and 74% of retained viability after incubation with uncoated and coated NPs, respectively), even though the conjugation of benzoyl peroxide increased cytotoxicity (90 and 74% of viability retained after treatment with unconjugated vs. conjugated benzoyl peroxide, respectively).^377^

GO was successfully applied as a carrier for the loading of photosensitizers, as reviewed recently.^378^ Recently, HA was conjugated to GO nanosheets with the subsequent physisorption of a photosensitizer.^379^ The efficient uptake of such modified GO NPs, with a size of 100 nm, was observed. The photosensitizer desorption led to enhanced NIR absorption and the generation of ROS in the cells. The authors reported IC_50_ values of 1 and 0.1 μg mL^−1^ of photosensitizer equivalent, respectively, when targeted cells were treated with either free or NP‐conjugated photosensitizer.^379^

**Figure 22:**
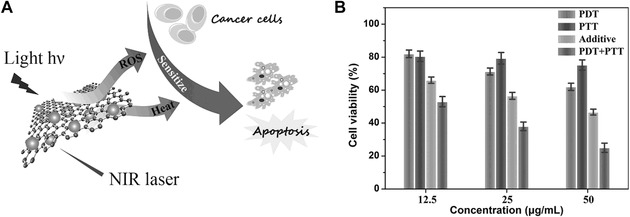
(A) Schematic illustration of sequential irradiation‐activated high‐performance apoptosis. (B) In vitro cell viability of MDA‐MB‐231 cells treated with rGO‐ZnO‐HA following PDT, PTT and combined PDT/PTT treatments. Reproduced from 322, with permission by John Wiley & Sons.

A system combining both photodynamic and photothermal effects has also been developed. While an rGO/ZnO hybrid is responsible for ROS generation upon light irradiation, NIR laser irradiation induced local hyperthermia^322^ because rGO effectively transferred heat to cells, with a local temperature increase upon exposure to NIR irradiation.^380^ To secure targeted treatment, HA was conjugated with the rGO/ZnO complex. The function and structure of rGO/ZnO‐HA NPs are depicted in Figure 22, where the viability of targeted cells is also depicted. From these plots, it is obvious that combined photodynamic and photothermal therapy decreased cell viability down to approximately 20%, much more than the application of only one type of treatment.^322^

Targeted irradiation by neutron beam is yet another way to induce the cytotoxic effect of certain agents, such as ^10^B ions using “boron neutron capture therapy.” Radionuclide ^10^B, after accumulating in the cells, captured supplied protons and underwent fission, generating heavily cytotoxic ^7^Li and ^4^He particles.^381^ To achieve a sufficient amount of boron in cells (on average 10^11 10^B ions per cell), mesoporous SiNPs were used to carry ^10^B coordinated to an organic ligand with high selectivity and an accumulation rate achieved by the decoration of the NPs with multivalent galactose units. The delivery efficiency was illustrated by having approximately 3 × 10^9^ atoms in cells when treated with unconjugated boron ligand compared to 10^11^ atoms when delivered by NPs. Preliminary results suggest that the NP‐delivered amount of ^10^B atoms is high enough to efficiently perform boron neutron capture therapy.^381^

### C. Cell Imaging

The principles applied to selective delivery can also be employed in cell imaging with appropriate carbohydrate‐binding receptors. Many studies have been published regarding the conjugation of glycan‐displaying NPs with dye molecules to facilitate detection in the UV‐VIS range, contrast enhancers for NMRI or computed tomography imaging of tissues or single cells accumulating nanocarriers.^382^

#### 1. UV‐VIS Probes

The recent trend is to combine the therapeutic and imaging effects in so‐called theranostic (i.e., therapeutic + diagnostic) NPs loaded with both an imaging probe and a drug as discussed above.^44^, ^381^, ^383^ The imaging function of the conjugate can also be combined with antiadhesive properties.^239^ Noteworthy, NIR‐absorbing GO/doxorubicin/HA NPs for combined photochemotherapy could be applied to imaging even without any additional dye or label incorporated due to the fluorescent signal obtained from a carrier drug (e.g., doxorubicin). Such an approach was applied for the visualization of cells that internalized nanocarriers.^281^, ^384^

**Figure 23:**
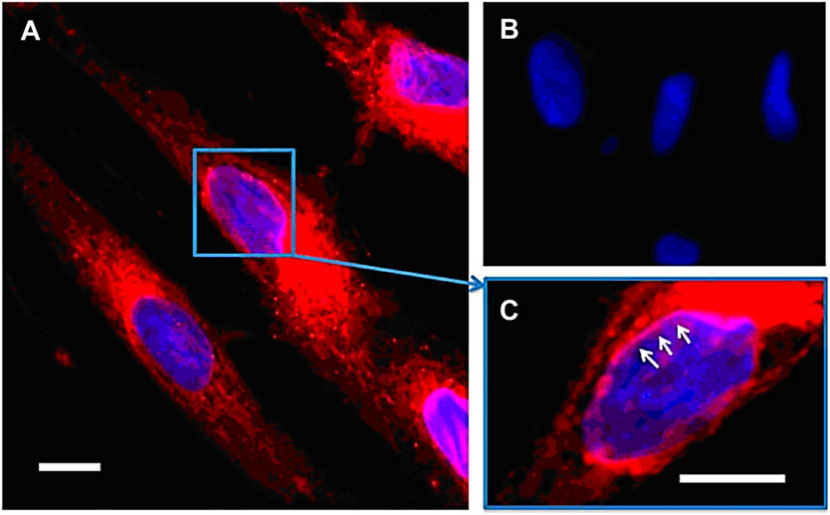
Effective intracellular delivery of rhodamine B octadecyl ester in HDF cells mediated by (1:9 PGalSMA34 + PGMA51) PHPMA270 vesicles (prepared via thin film rehydration to ensure sterility and enable loading with a fluorescent dye). Cells were incubated with 1.0 mg/mL rhodamine B octadecyl ester loaded vesicles for 16 hr. (A) Confocal microscopy image of live HDF cells: note the intracellular staining of membranes (red) after exposure to the rhodamine‐loaded vesicles, cell nuclei are counter‐stained blue using Hoechst 33342. (B) HDF cells treated with the same vesicles containing no rhodamine dye (negative control). (C) Higher magnification image obtained for (A): effective intracellular delivery of rhodamine dye allows selective staining of the nuclear membrane (white arrows). Scale bar: 50 μm. Reprinted with permission from 385. Copyright 2013 American Chemical Society.

Dye‐loaded NPs releasing their cargo after internalization promoted by surface‐displayed carbohydrate moieties were reported.^385^ The encapsulation of dyes into polymer NPs significantly increased the biocompatibility of imaging probes with the selective visualization of intracellular membranes of targeted cells (Fig. 23).^385^ Self‐assembled NPs prepared from a natural polysaccharide levan were able to encapsulate indocyanine green.^386^ The selective accumulation of the conjugate in tumor cells was mediated via overexpressed glucose receptors.^386^ NPs formed from a copolymer containing fluorescent rhodamine and glucose were successfully internalized by cells with asialoglycoprotein receptors enabling their fluorescent imaging.^387^ The chemical and enzymatic stability of the imaging NPs were very good, with a maximal release of only 2.5% of the loaded dye within 4 days in the presence of lipase and esterase.^387^

**Figure 24:**
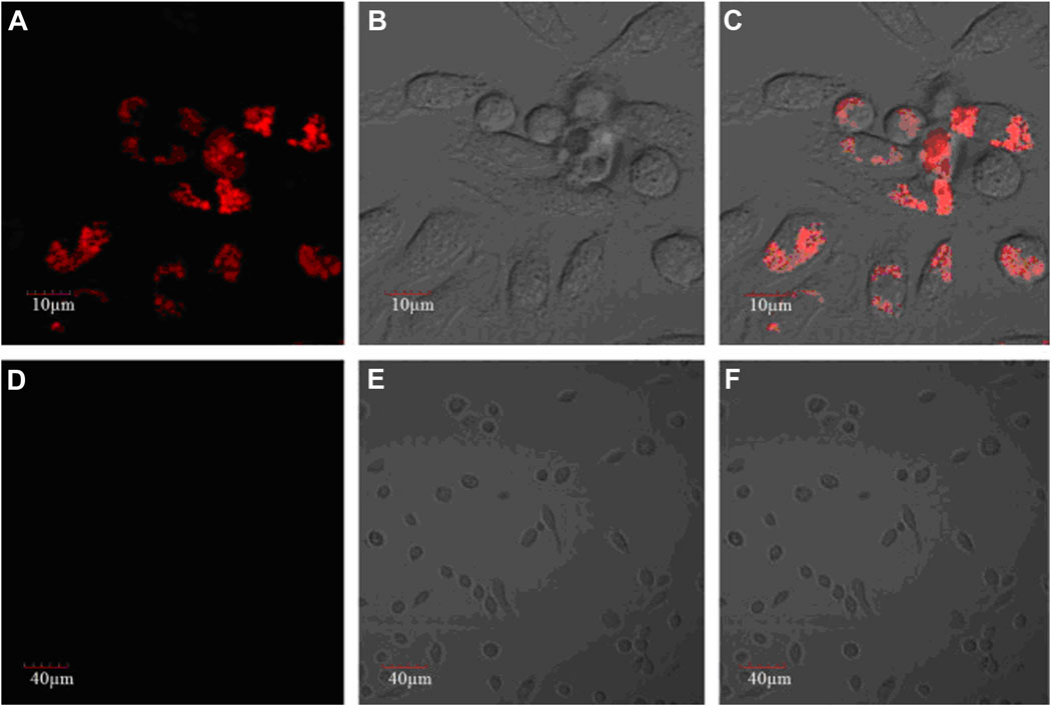
Confocal microscopic images of murine macrophage cells after incubation with water‐soluble glycocluster based on perylene bisimides PBI‐12‐Man (10 μg/mL, Man = mannose) without (a, excited at 559 nm; b, bright field; c, merging of photos a and b) or with mannose (18 mg/mL) inhibition (d, excited at 559 nm; e, bright field; f, merging of photos a and b) in PBS buffer at 37°C for 1 hr. As shown in a–c, the red fluorescence of PBI‐12‐Man was predominantly intracellular with punctate appearance, suggesting cell surface binding and endocytosis being the cell entry mechanism that results in vesicular (endosomal) localization. For the inhibition experiment, in the presence of *α*‐d‐mannopyranoside (d–f), the remarkable decrease of the red fluorescence of PBI‐12‐Man indicated the selectively binding interactions of PBI‐12‐Man with the surface mannose receptor of the macrophage cells. Reprinted from 389. Copyright 2014, with permission from Elsevier.

In addition to diagnosis, the dye labeling of NPs has also been used to evaluate the cell uptake rate. For example, the preparation of mannosylated micelles was optimized by the visualization of their uptake in the cells.^388^ Further in vivo experiments confirmed their selective imaging capability.^388^ Perylene bis(imide) derivative decorated with mannose was found to be fluorescent only after the degradation of the formed scaffold triggered by its ligation to a mannose‐specific lectin.^389^ The fluorescent imaging of macrophage cells overexpressing such receptors has been reported (Fig. 24).^389^ A novel NIR fluorophore activated upon isomerization at a low pH in lysosomes was used by Wu et al.^390^ Biocompatible poly(styrene‐co‐maleic acid) NPs loaded with this probe specifically targeting surface‐displayed sialic acid were able to selectively tag and mark tumors in vivo.^390^ Cell imaging using illumination outside the range of UV‐VIS and NIR has also been tested, for example, by Mäkilä et al., who radiolabeled (^68^Ga) therapeutic siRNA oligonucleotides conjugated with multivalent galactose units to evaluate their in vivo distribution.^391^ They observed that the valency of the displayed galactose was crucial to selective and efficient accumulation in liver, and the conjugate with seven glycan units exhibited the best performance, making them suitable for the further development of targeted genotherapy.^391^

**Figure 25:**
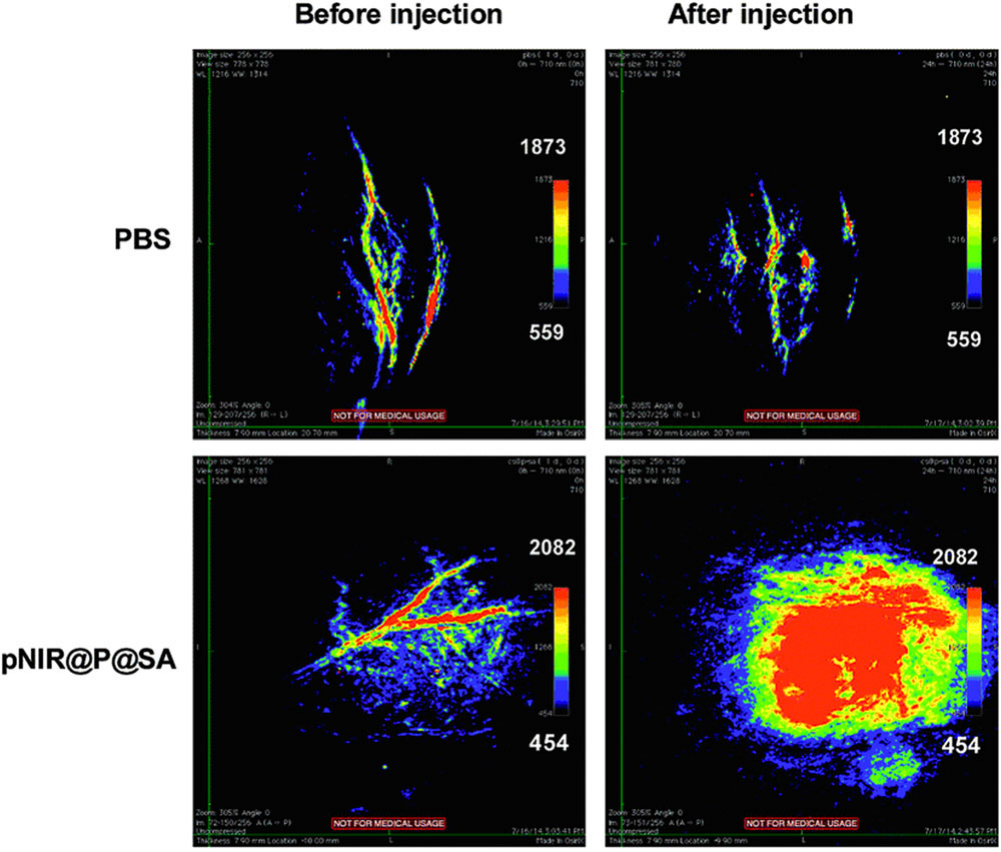
Photoacoustic tumor imaging in mice with sialic acid‐decorated polymer nanoparticles (pNIR@P@SA). Nude mice bearing H22 subcutaneous tumors were intravenously injected with phosphate buffer saline (PBS; 100 mL) or pNIR@P@SA (40 mg kg^−1^). The mice were imaged 24 hr after vesicle injection. Control images were obtained from mice before intravenous injection of pNIR@P@SA or PBS. A significantly higher photoacoustic signal obviously resulted from tumor issue after incubation with pNIR@P@SA (lower right panel). Reproduced from 390 with permission of the Royal Society of Chemistry.

**Figure 26:**
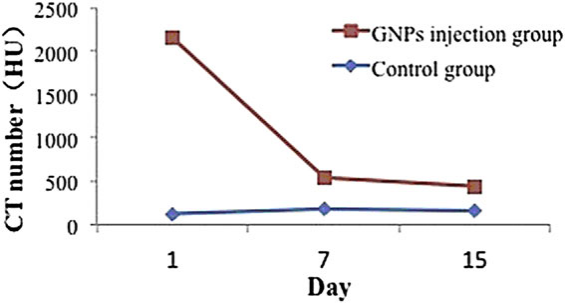
CT number for tumor versus time. The blue line is the CT number of the control group, whereas the red line represents for the PEG Glc‐GNP injection group. Reprinted from 401, with permission from Elsevier.

Other recent studies reported the preparation of simple nanocarriers based on synthetic^392^, ^393^ or natural^394^, ^395^ polymer NPs conjugated with glycans and imaging probes with selective adhesion to specific cell surface receptors, enabling the visualization of the surfaces of these cells. A complex study was carried out to visualize the binding of radiolabeled mannose‐containing polymer (γ‐Tilmanocept, approved by the FDA as a lymphatic node imaging probe) to targeted cells.^396^ The authors used a cyanine dye conjugated with Tilmanocept to specifically bind mannose receptors of macrophages, facilitating the delivery of a radiopharmaceutic drug.^396^ Multivalent clusters of iminosugars conjugated with pyrene or boron‐dipyrromethene cores exhibited fluorescence spectra applicable in cell imaging.^397^ As discussed earlier, multivalent iminosugars have been intensively tested as promising pharmacological chaperones.

The conjugation of nanocarriers with photosensitizers applied in photodynamic therapy has also often been used for simultaneous therapy and imaging since the majority of molecules applied as photosensitizers exhibit fluorescence. For example, a porphyrin derivative conjugated with glycopolymer exhibited an emission peak at 633 nm, allowing the real‐time visualization of the NP uptake with confirmed targeted cytotoxic effect.^375^ A similar effect was also observed with functionalized GO integrated into a pH‐sensitive HA nanogel serving as selective carrier for doxorubicin.^288^ The conjugate based on GO allowed both photodynamic therapy and fluorescence imaging,^288^ underlining the amazing versatility of graphene‐based nanomaterials.

Deeper tissue penetration and better resolution are the main advantages of photoacoustic imaging. In this method, a probe with an absorption maximum at longer wavelengths is used. The absorption resulted in changes in the probe's properties (thermoelastic expansion); hence, after the activation by a deep‐penetrating laser pulse, the probe was transformed into a detectable ultrasound signal.^398^ This imaging method was employed using sialic acid‐decorated polymer NPs loaded with a profluorophore, turning it into a photoacoustic probe by isomerization at a low lysosomal pH.^390^ In the end, it became evident that a significantly higher photoacoustic signal was achieved in tumor vein imaging using this method—see Figure 25.^390^

#### 2. Metallic NPs in Selective Cell Imaging

An inherent NIR‐induced fluorescence of small Au nanoclusters with the size of 2 nm was used in the preparation of selective imaging probes. Selectivity, stability, and biocompatibility were achieved by the conjugation of the nanoclusters with zwitterions and trivalent mannose ligands.^399^ The integration of the latter component into the conjugate increased the internalization rate by 62 and 256% when targeted dendritic cells were incubated with 1 and 25 μg mL^−1^ of the NPs, respectively.^399^

The absorption of energy by AuNPs from high‐frequency radiation has been employed to enhance the effectivity of radiotherapy (see Section 6.A.5) and as a contrast enhancer during computational tomography (CT) scans, as well. For example, in situ generated small AuNPs modified by dendrimers containing polyvalent lactobionic acid were reported to enter selectively hepatocarcinomic cells via their asialoglycoprotein receptors, allowing the high‐resolution computational tomography imaging of tumors.^400^ The same method for selective tumor visualization was also enabled by glucose‐modified AuNPs mediated via glucose transporters overexpressed on the surface of cancer cells.^401^ Tumors could be monitored for a week, as shown in Figure 26.^401^ Glucose‐conjugated AuNPs bearing a ligand with ^68^Ga as a tracer were biocompatible and excellent for in vivo imaging using positron emission spectroscopy.^402^ Moreover, neuropeptides loaded on glucose‐^68^Ga‐AuNPs enabled penetration through the blood–brain barrier.^402^

**Figure 27:**
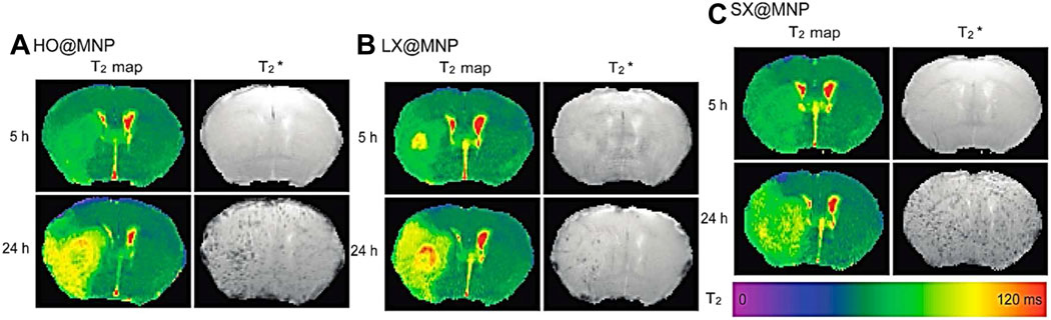
T2 maps and T2‐weighted images at 5 and 24 hr after MCAO in a representative animal tissue that received iron oxid‐silia nanoparticles decorated with (A) hydroxyl groups (HO@MNPs), (B) Lewis X without sialic acid (LX@MNPs), or (C) sialyl Lewis^X^ (SX@MNPs). Note: the color scale bar corresponds to the T2 value (msec). Reprinted with permission from 409. Copyright 2014 American Chemical Society.

Magnetic resonance is a broadly used imaging method in which the application of contrast agents significantly improves image quality, especially when these are in the form of NPs. In most experiments, iron oxide NPs decorated with stabilizing, targeting, or biocompatible materials have been tested.^403^ As an example, versatile glycopeptide grafting to magnetic iron oxide NPs was reported but without in vivo testing.^404^ A step further using sialic acid modified Fe_3_O_4_ NPs for the selective in vivo MR imaging of β‐amyloids applicable for the early diagnosis of Alzheimer's disease was reported.^405^ Mannose‐containing diblock copolymer grafted onto Fe_3_O_4_ NPs enhanced the carbohydrate receptor‐driven uptake into lung cancer cells with potential application in the diagnosis and localization of this type of cancer.^406^ HA‐grafted Fe_3_O_4_ NPs employed in the selective MR imaging of tissues with CD44 receptor‐overexpressing cells, that is, tumors, or local inflammations were also described.^407^, ^408^ A similar system with core Fe_3_O_4_ NPs having a thin golden shell covered with an HA layer enabling simultaneous MR imaging and photothermal therapy targeted toward cells with CD44 receptors has been reported.^370^ In order to increase the in vivo stability of the Fe_3_O_4_ core, its coating with a silica shell was described in several studies.^384^, ^409^ An enhanced imaging resolution of brain lesions in mice after simulated stroke can be observed in Figure 27, showing that NPs with different glycosylation exhibited different biodistribution.^409^ Sialic Le^x^ present on NPs allowed their accumulation in ischemic, that is, damaged, brain tissue due to selectin overexpression; NPs decorated with Le^x^ (without sialic acid present) could visualize such tissue less effectively, which was also confirmed by the software evaluation of dark and bright voxels in the images.^409^ Nevertheless, good results were also obtained with NPs bearing hydroxyls groups on the NP surface instead of glycans.^409^ In addition to the protective function of a silica shell, such a layer could provide a better environment for the covalent grafting of targeting ligands. The application of silica‐based nanocarriers for MR imaging has recently been reviewed by Caltagirone et al.^410^

In addition to Fe_3_O_4_ NPs, AuNPs were also applied in cell imaging. Small AuNPs of 1.8 nm in size grafted with lactobionic acid via PEG spacers were found to penetrate human hepatocellular carcinoma cells selectively via their overexpressed asialoglycoprotein receptors.^400^ While T2‐weighted MRI was used for contrast enhancement applying iron oxide NPs, T1‐weighing was used for the same purpose using AuNPs loaded with a Gd^3+^ complex.^411^ The modification of AuNPs by a combination of different carbohydrates helped to achieve a high imaging selectivity of tumor cells in mice models.^412^ AgNPs prepared by the reduction of Ag ions using saccharides present in sugar cane juice have ferromagnetic properties and could be applied for MRI.^411^

#### 3. Quantum Dots as Imaging Probes

**Figure 28:**
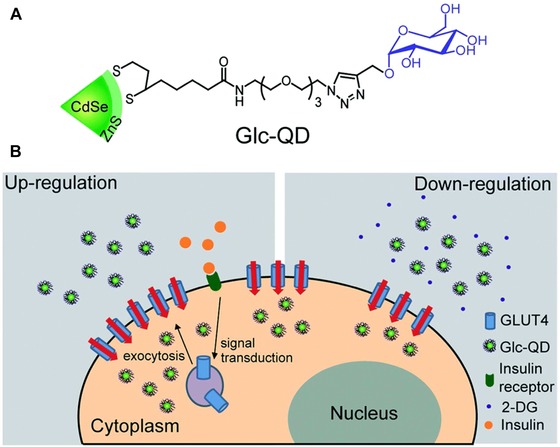
(A) A molecular structure of glucose‐functionalized quantum dots (Glc‐QDs). (B) A schematic illustration of the cellular uptake of Glc‐QDs regulated by insulin and 2‐deoxyglucose (2‐DG) in C2C12 muscle cells. Reproduced from 416 with permission of the Royal Society of Chemistry.

**Figure 29:**
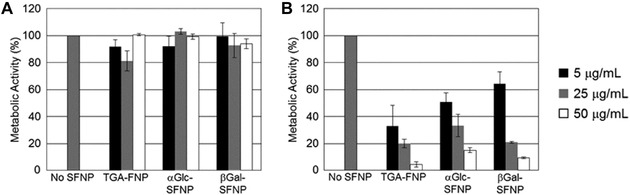
MTT assay for SFNPs. HepG2 cells were incubated with ZAIS/ZnS NPs (A) or CdTe/CdS QDs (B). The NP concentration was in the range of 5 to 50 μg mL^−1^ (left to right). Reprinted with permission from 417. Copyright 2014 American Chemical Society.

QDs as NPs composed of single nanocrystals of metal sulfides or other semiconductors have distinct optical properties resulting from their nano‐size. One of the most important features is their stable and adjustable fluorescence together with the overall stability inherent to all inorganic materials. Recent studies have reported that, after the encapsulation of QDs into a polymer shell with a final modification by carbohydrate moieties (for specific cell targeting), the resulted NPs still retain their fluorescence. The surface of QD‐modified NPs could be modified by a diverse range of functional groups with adjusted properties of NPs as reported by Schmidtke et al.^413^ This study suggested applicability of such NPs in in vivo imaging, even though this has not been tested.^413^ On the other side, the uptake and intracellular distribution of core‐shell CdSe‐ZnS QDs covalently grafted with different carbohydrates were assessed.^414^ In addition to lactose, which enhanced the uptake of the QD‐glycan NPs into cancer cells, the composition of a mixed carbohydrate shell (i.e., lactose and mannose vs. lactose and maltotriose) significantly altered the internalization of NPs.^414^ In another study, a secondary radiotracer (^125^I) was used as the probe conjugated with CdSe/CdS QDs decorated with a sialic acid‐binding lectin.^415^ These NPs were able to image breast cancer cells, as revealed by in vitro experiments.^415^ Enhanced selectivity in the targeted imaging of muscle cells was observed for glucose‐decorated QDs with insulin‐induced uptake mediated via glucose receptors, as illustrated in Figure 28, and such an approach could be applied for selective drug delivery as well.^416^ Since the vast majority of inorganic QD particles exhibit cytotoxicity, Shinchi et al. focused on the development of cadmium‐free ZnS‐AgInS_2_ NPs with lower cytotoxicity but still exhibiting good fluorescence properties and applicability in selective cell imaging after decoration with short targeting carbohydrates.^417^ From the graphs shown in Figure 29, it is obvious that all carbohydrate‐coated ZnS‐AgInS_2_ QDs exhibited minimum cytotoxicity against HepG2 cells (panel A), unlike Cd‐based QDs (panel B).^417^

QD‐like fluorescence was assigned also to GO‐based NPs,^378^ which were used, for example, for combined imaging and chemotherapy with an HA grafted as a targeting agent.^288^ It is worth mentioning that the replacement of semiconductor‐based mostly cytotoxic QDs with less harmful graphene‐based NPs would be another step to develop novel, more efficient theranostic particles with low cytotoxicity. Silica QDs possessing inner fluorescence exhibited low cytotoxicity, having a surface that could be easily modified with glycans.^418^, ^419^ Furthermore, fluoride nanocrystals doped with rare earth element ions were also suggested as possible substitutes for “classical” Cd‐containing QDs—see, for example, 420 and references therein.

#### 4. Toxicity of Nanomaterials

It is beyond any doubt that the massive application and employment of nanomaterials have brought and should bring in the near future amazing achievements and technical possibilities—in fact, many authors are talking about the nanoage or nano‐revolution. Nevertheless, the other side of this “nano‐enthusiasm” is the persistent uncertainty about the possible toxicity of NPs. This issue is particularly delicate in medical applications, where NPs are supposed to be delivered directly into the human body and interact with cells at a molecular level. It should be noted that these concerns have been raised simultaneously with nanomaterial applications—see, for example, a 1982 study on the toxicity of a potential drug carrier.^421^ In this study, one example of possible cytotoxicity mechanism of NPs was outlined, that is, the toxicity of monomers released from the intracellular degradation of NPs. Other known mechanisms, including unwanted aggregation of proteins, delivery of toxic molecules conjugated with NPs (including NPs from transportation and industrial emissions) and generation of highly cytotoxic ROS (the most toxic effect), were comprehensively reviewed.^422^, ^423^, ^424^, ^425^ The NPs’ behavior in living organisms is prevalently determined by their physical and chemical properties. For example, decreased NP size increases their active surface and, consequently, the rate constant of any catalytic reaction taking place on the NP surface. Moreover, the size also determines the biodistribution mode in an organism—see, for example, different modes of interactions of small and large graphene sheets with cells (while the former tend to disrupt cell wall,^426^ the latter more likely wrap it without any damage^427^). Another crucial aspect is the nature of NP surface modification, including (i) surface charge, the adjustment of which can turn otherwise harmless NPs, for example, into cell disruptors (see references in 423) or other biological functions,^428^ and (ii) the modification of NPs by biologically active moieties with examples, which can be found in sections of this review dealing with drug delivery, vaccine and other therapeutic effects, and cell imaging.

Some toxic impacts can be estimated from the physical or chemical properties of the NPs acquired from a precise and accurate characterization of the NPs. This is usually accomplished since physical and chemical characterization and at least in vitro cytotoxicity tests are included in practically any study concerning the administration of NPs into the human body (drug carriers, imaging NPs, etc.). Nevertheless, the correlation between in vitro and model animal in vivo studies may significantly differ from the real impact on humans, nota bene when the subacute effect is considered.^429^ Therefore, unless any negative health impacts are ruled out, appropriate precautions should be taken, and precise toxicity assessment must be performed in parallel with nanomedicine progress.

## 7. GLYCAN ENRICHMENT AND SEPARATION

NPs by definition are any objects with at least one dimension below 100 nm exhibiting unique physicochemical properties, which can be effectively applied to enhance the performance of glycan analysis by MS or to increase the efficiency of glycan enrichment. This is especially true due to the high surface‐to‐volume ratio, which can help to address problems with mass transfer; high surface area also means that the high density of active functional groups could be attached to NPs to increase separation efficiency or aid in MS analysis.^430^ NPs can be synthesized with controlled size and morphology, and it is also possible to prepare hybrid NPs with unique characteristics (e.g., MNPs covered by a gold film suitable for the formation of a SAM on their surface, etc.).^63^

SiNPs are chemically stable over a wide pH, a feature that is important for column packing material. Nanoporous silica materials, with a pore size spanning range from 2 to 150 nm with various modifications, are quite often applied as packing material for the separation of glycans. Other NPs, including metal oxides, CNTs, fullerene, and graphene, or GO can be used as packing/enrichment material either in their pristine or modified state. Recently, imprinted polymers have become popular because they are regarded as affinity matrices with tailored properties for the separation of a particular analyte with lectin‐like properties.^430^

### A. Glycan Release

**Figure 30:**
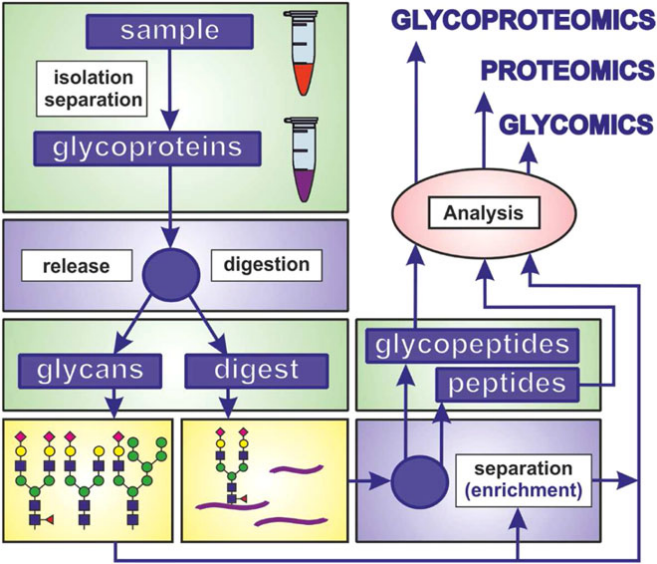
A graphical summary of different approaches, which can be applied for glycoprotein analysis including glycomics (analysis of released glycans), proteomics (identification of peptides after removal of glycans) and glycoproteomics (characterization of intact glycoproteins in order to indentify a site‐specific connectivity between glycans and proteins).

Glycans attached to the protein backbone can be analyzed either in their intact form (i.e., glycoproteins), after protein digestion by endopeptidases or endoproteinases (i.e., glycopeptides) or as pure glycans released from the protein or peptide backbone using the enzyme PNGase F or PNGase A (Fig. 30). The release of glycans from *O*‐glycans can be done not using enzymes but rather chemical reaction, that is, β‐elimination or the use of hydrazine is needed. When glycopeptides are produced from glycoproteins by a tryptic digest, glycopeptides are only a minor component of the tryptic digest, and their enrichment is important for high‐performance MS analysis. Peptides isolated from glycopeptides can be further applied for protein fingerprinting.^431^, ^432^

### B. Mass Spectrometry

MALDI‐time‐of‐flight‐MS (MALDI‐TOF‐MS) is a gentle technique allowing the analysis of large biomolecules in both positive and negative ionization modes. This is why this technique is the most commonly applied in glycan, glycopeptide, or glycoprotein analysis, even though the MALDI‐TOF process itself is not yet fully understood.^433^ On the other hand MALDI‐TOF and electrospray ionization (ESI) might not distinguish glycan isomers when operated in a conventional mode. Various on‐line separation techniques (i.e., using porous graphitized carbon packed columns), which can be coupled to ESI MS, can effectively resolve this problem.^434^ Alternatively, tandem MS, that is, MS/MS or MS^n^, can resolve this issue as well.^435^ MALDI‐TOF compared to ESI exhibits higher sensitivity for the determination of glycans and efficiently ionizes molecules with a higher molecular weight. At the same time, MALDI‐TOF is more tolerant to contaminants.^435^ MALDI‐TOF when compared to ESI introduces higher energy to the sample, resulting in the fragmentation of labile functional groups, including sulfates, phosphates, and sialic acids.^435^ Since sialic acids are quite labile molecules with significant involvement in many biological processes, an analysis of sialic acids requires the development of special analytical strategies.^436^, ^437^, ^438^, ^439^ Moreover, a proper matrix must be chosen to effectively generate ions of large molecules for MALDI‐TOF analysis, and as for ESI, the sensitivity of detection decreases with the increasing molecular weight of glycans.^435^

### C. Glycan Enrichment

**Figure 31:**
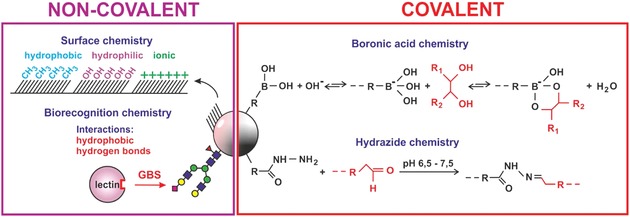
Various ways glycans can be enriched using hydrophobic, hydrophilic, or ionic interactions (upper left part) or biorecognition interactions involving glycan binding proteins (lectins, lower left part). Alternatively for glycan capture/release particles modified by boronate functional groups or hydrazine can be applied, as well.

There are few main reasons why glycans have to be enriched prior to MS analysis: (i) the glycan level in the studies sample can be well below detection limit of an MS instrument; (ii) glycopeptides or glycans must be removed from peptides due to their lower abundance compared to peptides; and (iii) the ionization of glycans or glycopeptides in the presence of peptides is frequently less efficient.^63^ Glycans, glycopeptides, or glycoproteins can be enriched in numerous ways, including boronate affinity, hydrazide chemistry, lectin affinity, hydrophilic interactions, and other approaches (Fig. 31).^440^

#### 1. Boronate‐Based Enrichment

Boronate‐modified materials are applied for glycan enrichment due to the strong interactions between boronate functional groups and *cis*‐diols present in glycans under basic conditions, that is, at pH 8–9.^441^ The main prerequisite for glycan binding to boronate‐modified material is the choice of a proper pH, which should be above the p*K*_a_ of the boronate moiety of a boronate‐terminated molecule (e.g., p*K*_a_ = 8.9 for frequently applied PBA). Sialic acids are exceptions to this rule since the effective binding of SA to boronate‐modified material can occur at a pH lower than the p*K*_a_ of boronate functional group. Recent studies however suggest that besides proper choice of pH, other factors come into play during the interaction of glycans with boronate‐modified material, such as steric factors, and the nature of buffer components.^442^ Decreased pH can be effectively applied to release captured glycans for further analysis. Relatively high pH needed for effective glycan enrichment based on boronate affinity can be a problem for quite complex biomolecules. Such glycopeptides or glycoproteins can become unstable at a high pH. To counter this, various strategies have been applied to lower the pH needed for such binding, including the proper choice of substituents (electron withdrawing groups) in proximity to boronate functional groups.^443^ Alternatively, benzoboroxozole derivatives can be applied to lower the pH required for glycan binding via boronate affinity.^444^ Such approaches are also useful due to the widening of the applicable pH window for glycan enrichment.

#### 2. Hydrazide‐Based Enrichment

Hydrazide‐modified materials can be efficiently applied for glycan enrichment, but glycans must be oxidized first to form aldehydes from *cis‐diol* groups. Such aldehydes react with hydrazide‐forming hydrazone bonds, which are stable under basic conditions, and glycan can be released from enrichment material by the application of acidic pH, similar to glycan release from boronate affinity materials. Alternatively, PNGase F could be applied for the release of glycopeptides.^445^, ^446^

#### 3. Oxime‐Based Enrichment

Oxime‐modified surfaces can be a good alternative to the hydrazide‐based enrichment of glycans since the process of enrichment can be considerably shorter (1 hr) compared to hydrazide‐based enrichment (12–16 hr), with high enrichment sensitivity with LOD in the low femtomole range and with the selectivity of glycopeptide enrichment even in the presence of a 100‐fold excess of nonglycopeptides. Glycopeptides must be oxidized in order to interact with oxime functionalities, and overnight incubation with PNGase F was required for glycan release for subsequent MALDI‐TOF‐MS analysis.^447^

#### 4. Hydrophilic Interactions

A hydrophilic interaction‐based method for glycan enrichment involves the application of a polar stationary phase and a nonpolar mobile phase for separation. Analytes partition between a water‐rich layer on the surface of the stationary phase and the mobile phase. Glycans as hydrophilic molecules will be attached to the column/material, while other more hydrophobic molecules (including peptides) will be dissolved in the mobile phase and washed away. Hydrophilic interaction based on zwitterionic functional groups has become popular in recent years due to the ability of zwitterions to form a stable water layer on the surface of the separation phase, which is effective for glycan partitioning.^430^

#### 5. Lectin‐Based Enrichment

A lectin‐based approach for glycan enrichment is based on the natural bioaffinity interaction between glycans and glycan‐binding proteins (lectins) triggered by the interaction of glycans, especially with aromatic stacking amino acids and amino acids providing hydrogen bonds.^448^ The application of lectins for glycan enrichment is advantageous compared to boronate, hydrophilic interaction or hydrazide‐modified materials due to the higher degree of specificity for some glycan structures, but selectivity and binding affinity are weaker compared to antibody‐based biorecognition. Moreover, lectins can distinguish minor changes in glycan structures. A good example is SNA, recognizing sialic acid bound to galactose via an α‐2,6 bond, while *Maackia amurensis* agglutinin binds to α‐2,3‐linked sialic acid. Another example is the arm‐specific recognition of bisecting glycans (i.e., glycans with two arms).^449^ Thus, not all glycans present in the sample will be enriched using lectins, and a lectin‐affinity enrichment strategy can be applied to fractionate glycans into several groups. Finally, glycans can be released from lectins by the application of free carbohydrates or by exposure to an acidic eluting buffer.

#### 6. Other Ways for Enrichment

There are other options for glycan enrichment, such as reverse‐phase mode, electrostatic mode (for glycans containing negatively charged sialic acids), electrostatic repulsion hydrophilic interactions, and affinity capture based on protein A, which is applicable for the selective enrichment of antibodies with the subsequent glycoprofiling of glycans present especially in the Fc fragment of IgGs.^21^, ^430^

### D. Nanoporous Materials

Porous materials are, according to IUPAC definition, considered nanoporous only if the pore size is below 1 μm, but earlier terminology defining materials as macroporous (above 50 nm), mesoporous (2–50 nm), and microporous (below 2 nm) is frequently used in the literature.^450^ In the forthcoming paragraphs we will stick to the latter division of nanoporous materials into three categories. The other important parameters of porous materials are distribution of pore size, pore shape (cylindrical, slit shaped, funnel shaped, etc.), and degree of pore interconnections. The porosity of the given material can be characterized by the total pore volume and a specific surface area. Such porous materials are quite frequently applied as packing material for the separation columns for the enrichment/separation of glycoproteins, glycopeptides, or glycans and have been reviewed in the past.^430^, ^446^, ^451^, ^452^ The size and modification of pores play a prominent role in interactions with ligands due to the confinement effect.^453^ For example, when glycoprotein (ribonuclease B) interacted with a mesoporous material, the enhancement factor (i.e., *K*_D_ of the interaction with mesoporous silica compared to non‐porous silica) increased from 45 to 900, when, in addition boronate affinity, electrostatic interactions were also involved. Upon the decreased pore size of mesoporous silica from 2.6 to 2.1 nm, a further increase in the enhancement factor to 2100 was observed.^453^ This indicates that for effective ligand capture, the careful design of surface chemistry and the morphology of porous materials are important.^454^ An interesting approach for how to increase the surface area of composites (i.e., from 69 to 93 m^2^ g^−1^) while decreasing the pore size (from 7.6 to 5.0 nm) by mixing GO sheets into composites was demonstrated by He et al.^455^

#### 1. Silica‐Based Nanoporous Materials

**Figure 32:**
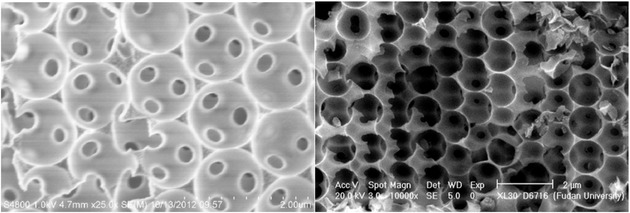
SEM image of hierarchically ordered macro/mesoporous silica material before (left) and after (right) boronate modification. Reproduced from 456 with permission by John Wiley & Sons.

Porous silica material with an ordered/controlled size and shape of large pores (1 μm) was prepared by Yan et al.^456^ using polystyrene colloidal crystals as a hard template, with mesopores of 4.6 nm using a surfactant as a soft template and applying tetraethylorthosilicate (TEOS) as a silica source. Moreover, macropores were interconnected via pores of 50 nm in diameter (Fig. 32). The specific surface area of the material was 255 m^2^ g^−1^ with a pore volume of 0.46 cm^3^ g^−1^. Such hierarchically ordered material was then modified with 4‐vinylphenylboronic acid to form polyboronic acid‐modified material, which was successfully applied for the effective enrichment of glycopeptides prepared by the tryptic digest of a model glycoprotein ‐ HRP. The method using glycan enrichment (10 min) revealed the presence of 12 glycans by MALDI‐TOF MS, while only four glycans were obtained without any glycan enrichment. The glycan of HRP could be detected with an LOD of 1 ng μL^−1^ (1 fmol) even in the presence of BSA, which was in 50:1 excess compared to HRP.^456^

Mechanically stable silica microparticles of 1.6 μm in diameter with macropores ranging in size from 50 to 150 nm were applied to immobilize two lectins, Con A and *Aleuria aurantia* lectin, by Mann et al.^457^ Such particles with interconnected pores and with a high surface area of 200 m^2^ g^−1^ accommodated a high density of immobilized lectins (16 mg g^−1^). When lectin‐modified particles were applied as a support for loading a chromatography column, up to 75 μg of glycoproteins could be bound to the column (50 × 1 mm) with immobilized Con A. Finally, the columns were used to enrich glycoproteins from just 1 μL of blood serum after removing IgG and albumin. Subsequently, the glycomic and glycoproteomic profiling of bound glycoproteins was performed using MALDI TOF/TOF MS.^457^

A capillary with boronate‐modified mesoporous walls was used for the selective enrichment of neutral or acidic glycans via pH manipulation by Lu et al.^458^ When the binding solution had a pH lower than the p*K*_a_ of boronate by one pH unit or more, the boronate‐modified capillary column captured sialylated glycoproteins. In contrast, when the pH of the binding buffer was higher than the p*K*_a_ value of boronate by one pH unit or more, glycoproteins containing SA were electrostatically repelled from interaction with the monolith, and glycoproteins with neutral glycans were preferentially captured. Furthermore, the ionic strength of the binding buffer played an important role in the capture of sialylated glycoproteins.^458^

A boronate‐modified affinity column was also helpful in the identification of DNA aptamers binding to glycoproteins using the systematic evolution of ligands by exponential enrichment as proposed by Nie et al.^459^ The boronate column served as a matrix for binding glycoprotein (HRP), with the complete process performed on the column; the authors identified seven DNA aptamers binding HRP with an affinity constant down to 10 nM.^459^ DNA aptamers selected specifically against glycoproteins can be applied for the selective capture of some glycoproteins in the future since their *K*_D_ (10^−8^ M) can be lower than that of lectins.^459^

#### 2. Carbon‐Based Nanoporous Materials

TEOS with a surfactant as a structure‐directing agent was applied by Qin et al.^460^ to prepare SiNPs with a uniform size of 70 nm. The particles with a surface area of 901 m^2^ g^−1^ with a pore size of 2.8 nm were treated with sulfuric acid and subsequently carbonized with the appearance of sp^2^‐hybridized aromatic carbon structures, which were effective in glycan enrichment. Thirteen *N*‐glycans released from just 5 ng of ovalbumin (i.e., LOD of 100 fmol) by PNGase F could be detected. Finally, the particles were applied for the enrichment of *N*‐glycans from 50 nL of human serum and analyzed by MALDI‐TOF‐MS. Eight new *N*‐glycans that were not observed in the MS spectrum when standard carbon adsorbent was applied were observed, including low‐abundant fucose and high‐mannose glycans.^460^

Mesoporous silica composites were prepared by Sun et al.^461^ using a graphene layer as a support. In this case, graphene was oxidized by nitric acid to deposit oxygen‐rich functionalities, which were used in the subsequent step for the formation of a mesoporous silica layer by the deposition of TEOS in the presence of a surfactant as a structure‐directing agent. Finally, the whole nanocomposite was carbonized to transform the surfactant into a carbon layer. The resulting material exhibited a uniform pore size of 2.8 nm with protein‐excluding properties with a surface area of 372 m^2^ g^−1^. MALDI‐TOF‐MS showed the presence of 25 *N*‐linked glycans released from ovalbumin when glycan enrichment with graphene‐based nanocomposite was applied, while enrichment with active carbon material was less effective in terms of the number of glycans detected and the signal intensity obtained. MALDI‐TOF‐MS analysis without any enrichment did not show any sign of glycans.^461^

Commercially available mesoporous graphitic carbon with a pore size of 25 or 30 nm was applied by Zhao et al. as a packing material for fully automatable two‐dimensional liquid chromatography for high‐throughput proteomic and glycomic analysis of various lysates/samples.^462^ With this fully automated approach, it was possible to identify up to 2678 proteins, 11,984 unique peptides in neurons or up to 130 *N*‐glycoproteins, 705 *N*‐glycans, and 254 glycosylation sites in macaques plasma (*Macaca fascicularis*) with a total analysis time of 19 hr using ESI‐MS/MS. According to authors, the technology offered unattended, robust, and scalable analyses of samples on a submicrogram scale when sample injection was performed once a day, and the system was able to operate continuously for up to 14 days without experiencing any major problems.^462^

A porous silica column was applied for the covalent immobilization of PNGase F by Jmeian et al., and such a column was used for the continuous (online) release of neutral and acidic glycans from glycoproteins with subsequent continuous glycan enrichment in a column loaded with porous graphitic carbon and with a subsequent analysis of glycans.^463^ The capillary was coupled to a C_8_ trap and a porous graphitic carbon HPLC‐chip and finally interfaced to perform liquid chromatography‐MS and liquid chromatography‐MS/MS analyses. The main advantage of the system is the short time (6 min) needed to release glycans compared to the usual overnight incubation at 37°C. Moreover, glycans could be effectively analyzed from just 100 fmol of protein or 0.1 μL of human blood serum.^463^ The time needed for online glycan removal is shorter compared to that needed for the microwave‐assisted release of glycans in the presence of PNGase F (30 min at 37°C).^464^

GO was due to the large surface area (theoretical relative surface area of 2630 m^2^ g^−1^) effectively applied by Ren et al. as a support for the covalent immobilization of a high amount of PNGase F (688 μg g^−1^ of GO, which is threefold higher compared to SiNPs). Highly efficient *N*‐glycan release from a protein backbone of two model glycoproteins (ribonuclease B and asialofetuin) can be completed within 2 min, and the enzyme can be reused. The stability of the enzyme complex is remarkable, that is, up to 8 weeks, when stored at 4°C, and as low as 2 μg μL^−1^ of the enzyme complex was needed to analyze the plasma extracts.^465^

#### 3. Other Nanoporous Materials

Hydrophilic mesoporous phenol‐based material with added PEI, prepared by Jin et al. with a template‐free method, exhibited a large surface area (548 m^2^ g^−1^) with 13 nm^466^ mesopores. When the amount of PEI changed, it was possible to change both the porosities and surface area of the material. Glycopeptides could be detected with an enrichment factor (MS intensity ratio after and before enrichment) of 62 with a recovery index of 80.4%. A tryptic digest of IgG could be detected down to 5 fmol with only a moderate improvement compared to commercial hydrophilic interaction chromatography beads (40 fmol).^466^

A capillary modified with a polymeric material with an introduced –SH group having a surface area of 30.3 m^2^ g^−1^ was applied for the further selective modification of the surface with AuNPs (13 nm) by Wu et al.^467^ The gold surface was further modified by two thiols terminated in boronate and amine functional groups making an intramolecular B‐N coordination for the enhanced specificity of glycoprotein capture with a binding capacity of 0.39 mg g^−1^. The capillary was applied for the selective enrichment of a glycoprotein over a protein (1:1000 ratio) with a recovery of 85% and finally applied for the glycoproteomic profiling of just 9 μg of human plasma with 160 glycoproteins identified by MS.^467^

**Figure 33:**
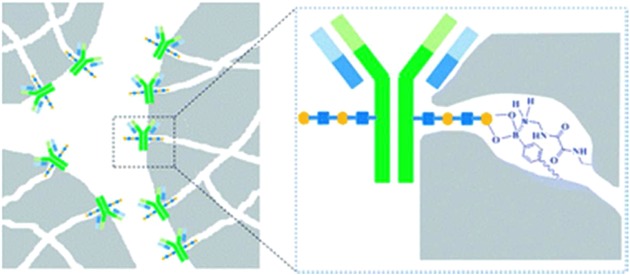
A scheme showing a specific recognition of IgG by the monolith exhibiting protein A‐like binding with presence of bimodal pores (macropores and mesopores) modified by boronate functional groups. Reproduced from 468 with permission of the Royal Society of Chemistry.

A boronate‐modified affinity monolithic column with a surface area of 13.7 m^2^ g^−1^ and with bimodal pores containing mesopores (3.9 nm) and macropores (1.4 μm) was prepared by Liu et al. using ring‐opening polymerization to mimic the function of protein A to bind antibodies.^468^ In this case, mesopores could be accommodated by the glycan of IgG via the interaction of glycans with boronate, while the protein backbone of IgG could not enter the mesopores (Fig. 33). The binding capacity of such material for IgG binding was comparable (23.7 mg g^−1^) to a number of protein A mimics. The monolith was a quite stable and cost‐effective alternative to using protein A with the price of 40 USD per 1 g. Moreover, the IgG attached to the monolith still exhibited its binding affinity toward antigens.^468^

### E. Imprinted Polymers

MIP can be effectively applied for the selective capture and release of analytes because the analyte molecule is imprinted, and after the formation of a thin layer of polymer around the template (analyte), the analyte is removed from the MIP. Thus, such MIP is then able to selectively recognize the analyte over other molecules.

**Figure 34:**
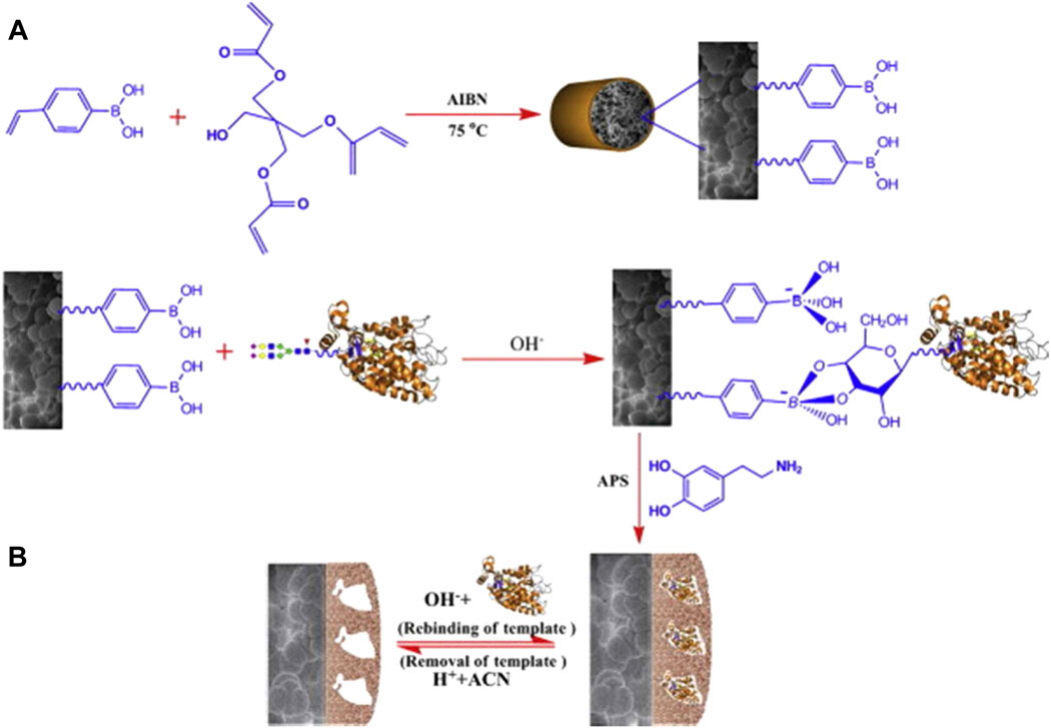
(A) Preparation of vinylphenylboronic acid‐based molecularly imprinted monolith with polydopamine coating and (B) its recognition mechanism toward glycoproteins. In the figure the following four major steps are shown: (1) preparation of the VPBA‐based polymeric skeletons; (2) reversible immobilization of glycoprotein via boronate affinity interaction; (3) self‐polymerization of DA on the surface of the glycoprotein‐immobilized boronate affinity monolithic skeletons; (4) the formation of glycoprotein‐imprinted monolith after removal of template that is complementary in shape, size, and functionality with respect to the template. Reprinted from 469. Copyright (2013), with permission from Elsevier.

Such an approach was applied to imprint HRP as a model glycoprotein on the inner wall of the capillary tube modified by boronate functionality as described by Lin et al. (Fig. 34).^469^ Then, a polydopamine film was formed around HRP with the final removal of HRP. Such an MIP was able to recognize HRP with a bound amount of 4.6 mg g^−1^ over other glycoproteins, while the boronate‐modified capillary also effectively captured other glycoproteins.^469^

**Figure 35:**
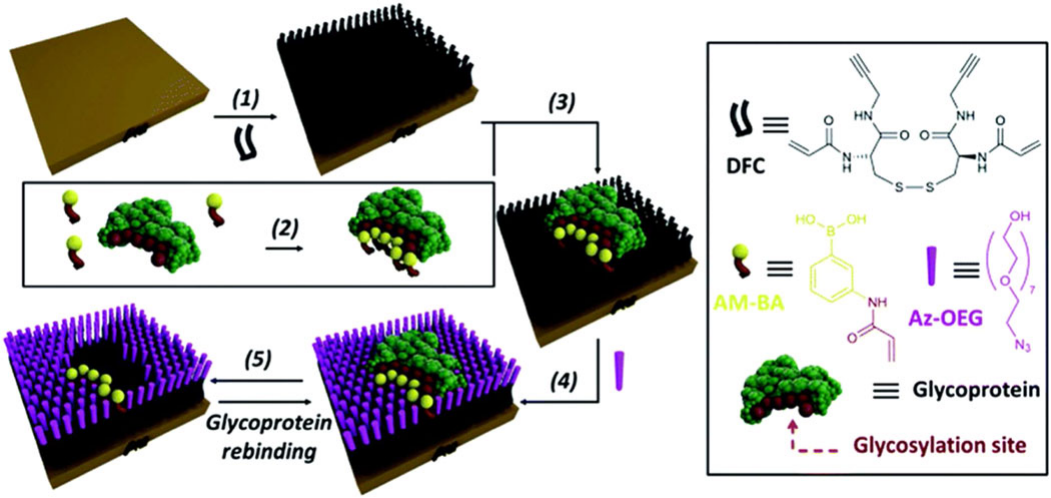
Experimental design for the formation of surface‐restricted click‐imprinted binding sites for glycoproteins. Disulfide dimer (DFC) SAMs were prepared by immersing clean gold substrates in 0.1 mM methanolic solutions of DFC for 24 h (step 1). In step 2, BA receptor units are introduced via (3‐acrylamidophenyl)boronic acid (AM‐BA) that is incubated for 30 min at an optimized pH (8.5) with a template target glycoprotein. Multiple boronate esters are formed reversibly between the AM‐BAs and the carbohydrate structures of the glycoprotein template. The pre‐assembled glycoprotein–AM‐BA complex is then grafted on the DFC SAM *via* acrylamide co‐polymerization, affording the creation of spatially arranged sets of BAs on the surface that are specific for the target glycoprotein (step 3). In order to provide complimentary allosteric specificity, a mould or imprint is created around the glycoprotein template at the surface by so‐called click chemistry functionalization of the alkynes of the DFC on the SAM by reacting azide‐terminated heptaethylene glycol (Az‐OEG) moieties with the terminal alkynes on the DFC SAM via a copper‐catalysed alkyne–azide cycloaddition (CuCAAC) reaction (step 4). The glycoprotein targets are removed by washing under acidic conditions (step 5). Reproduced from 470 with permission of the Royal Society of Chemistry.

An interesting imprinting approach for the selective binding of a glycoprotein was recently applied by Mendes et al. (Fig. 35).^470^ Gold surface was modified by an acrylamide‐alkyne cysteine derivative to make a thin film terminated in alkyne and acrylamide functionalities. Then, a glycoprotein was incubated with (3‐acryl‐amidophenyl)boronic acid when boronate functional groups interacted with a glycan part of the glycoprotein (PSA), and the other functional groups were applied for the attachment of PSA on the surface via acrylamide copolymerization. Finally, the surface was blocked by the addition of OEG‐N_3_ molecules via click chemistry. Because the boronate–glycan interaction can be disrupted by low pH, a simple pH lowering was applied to remove the PSA template from the surface. Such an approach exhibited a 30‐fold higher affinity for its target compared to that of other (glyco)proteins.^470^ Even though this imprinting strategy was applied for SPR sensing, there is a possibility of using this approach for selective PSA enrichment from a complex sample.

In their study, Liu et al. focused on finding the relationship between the thickness of the imprinted polymer and the size of the imprinted glycoprotein regarding the binding efficiency.^471^ Three glycoproteins (ribonuclease B, HRP, and GOx) were imprinted within a polydopamine film deposited over magnetic particles modified by boronate functional groups. The thickness of the polydopamine‐deposited film (5.7, 10.2, 16.3, and 25.3 nm) was controlled by polymerization time (1, 3, 6, and 15 hr). The higher the size of the glycoprotein (15–80 kDa), the thicker the film (5.7–16.3 nm) needed to be to achieve maximal binding capacity (16.7–19.5 mg g^−1^).^471^

Polymers with imprinted glycans isolated from two model glycoproteins—ribonuclease B and transferrin—were prepared by Bie et al. on 100 nm boronate‐modified MNPs.^472^ Glycans released from a glycoprotein were attached to boronate‐modified MNPs, and glycans were imprinted by forming a thin (2.5 nm) silane layer. Finally, the glycan template was removed, and such NPs were applied for the enrichment of glycoproteins. The imprinting ratio (i.e., the ratio of glycoprotein attached to imprinted vs. non‐imprinted NPs) was 8.4 for ribonuclease B and 21.8 for transferrin, with a maximal sorption capacity of 1.4 mg g^−1^ and a *K*_D_ of 25 μM, suggesting only moderate glycan enrichment. The transferrin glycan‐imprinted polymer was applied for the enrichment of transferrin from a human serum sample.^472^

### F. Magnetic NPs (MNPs)

**Figure 36:**
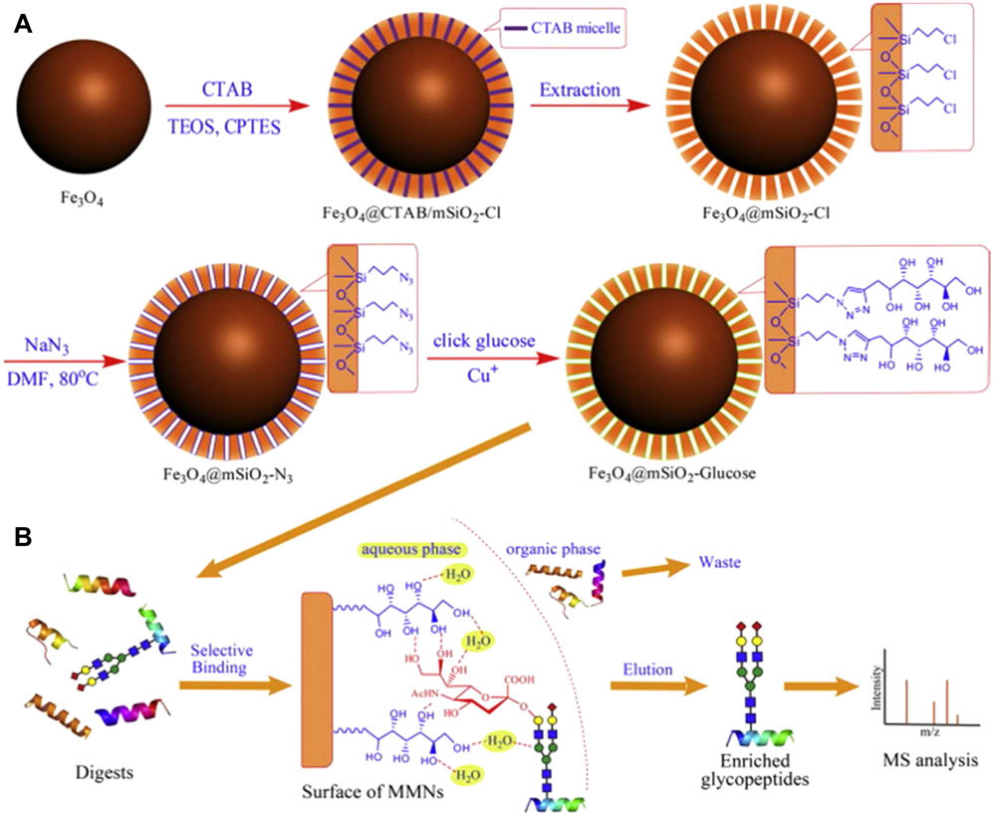
(A) Schematic representation of the synthesis of hydrophilic MMNs and (B) the selective enrichment process for glycopeptides using hydrophilic MMNs. Reprinted from 473. Copyright 2014, with permission from Elsevier.

Glycan enrichment can be effectively performed using MNPs with different glycan‐recognizing functional groups present on the surface of such NPs. Core‐shell NPs with an MNP (Fe_3_O_4_‐based) core and silica shell having uniform 2.2 nm mesopores (formed by the removal of a surfactant from the shell layer) were prepared by Zheng et al.^473^ Such NPs were further activated, and glucose was covalently linked to it by click chemistry (Fig. 36) to provide hydrophilic interactions. Such particles exhibited a large surface area (324 m^2^ g^−1^), high ability to enrich glycopeptides (250 mg g^−1^), high sensitivity (50 fmol), short incubation time (5 min), and high recovery (94.6%) using ESI MS analysis. When 0.25 μL of human serum without enrichment was analyzed, only 12 *N*‐glycans were found, while 42 *N*‐glycans were identified after the application of the MNP‐based enrichment step in the ESI MS spectra.^473^

MNPs were modified by Cao et al. using a polymer to which hydrazide was attached, forming a 3D matrix for glycan enrichment with a threefold higher loading of hydrazide compared to single‐layer NPs.^474^ The enrichment of glycopeptides was effective even in the presence of a 100‐fold excess of peptides with a recovery index of 78% and with a binding capacity for glycopeptides of 25 μg mg^−1^. Glycopeptides enriched from mouse liver tissue were analyzed by MALDI‐TOF‐MS.^474^

In order to enhance the hydrophilicity of the MNPs, Chen et al. covered such particles by a thin layer of SiO_2_ to which zwitterionic molecules were attached by precipitative polymerization.^475^ Such particles possessing a highly hydrophilic surface were effective in glycan capture, when by MALDI‐TOF‐MS, glycans from just 0.1 fmol of IgG could be detected with a high binding capacity of 100 mg g^−1^ and high enrichment recovery of 74% with a rapid magnetic separation. Finally, the particles were applied for the glycoprofiling of 65 μg of proteins extracted from mouse liver.^475^

MNPs with co‐precipitated ethylenediaminetetraacetic acid having a size of 15 nm were modified by Dong et al. using Cu(II) with the subsequent high loading of Con A lectin (up to 28 wt%).^476^ Such particles were applied for the enrichment of a model glycoprotein, namely ovalbumin, with a binding capacity of 72 mg g^−1^, while a high affinity with *K*_D_ of 38 nM was observed. Glycoproteins were successfully enriched, even in the presence of a non‐glycoprotein in molar excess of 600:1 with a fast magnetic separation within 15 sec.^476^

MNPs with a size of 15–20 nm during synthesis formed larger aggregates with a size of 140 nm and finally were modified by chitosan using the one‐pot method as described by Fang et al.^477^ Such particles were able to detect glycans from as low as 8 fmol of a tryptic digest of IgG using MALDI‐TOF‐MS with a binding capacity of 17.5 mg g^−1^. Finally, 45 μL of tryptic digest from HeLa cells was successfully glycoprofiled.^477^

Magnetic mesoporous (pores with 3.8 nm) particles with a surface area of 211 m^2^ g^−1^ and with a total pore volume of 0.38 cm^3^ g^−1^ were applied by Deng et al. for the enrichment of as low as 10 fmol of a model glycoprotein in 300‐fold excess of protein (BSA), and finally, glycans enriched from human serum were determined by MALDI‐TOF‐MS.^478^

Liu et al. applied MNPs (*d* = 100 nm) patterned by a PAMAM dendrimer further modified by boronate functional groups for model glycoprotein (HRP) enrichment.^479^ Boronate present on a dendrimer modified MNPs had a 3–4 orders of magnitude higher affinity constant for glycoproteins compared to single boronate, while glycoprotein enrichment was possible even in a 1 million‐fold excess of competing monosaccharide with an efficiency of 42–88%. HRP could be adsorbed with density of 21 mg g^−1^ of support with LOD of 180 amol with extraction equilibria reached within 1 min (with desorption equilibria of 5 min). The reusability of glycoprotein enrichment was highly reproducible, with an RSD of 7.5% for five consecutive enrichment steps. Finally, the approach was also applied for the analysis of glycoproteins in human saliva.^479^

Deng et al. applied MNPs (100 nm) modified by poly(styrene‐co‐vinylbenzene‐boronic acid) for glycopeptide enrichment.^480^ Such particles could detect glycopeptides down to 125 fmol from a tryptic digest of HRP, even in 120‐fold excess of peptides. Glycopeptides enriched from human serum were analyzed using MALDI‐TOF‐MS.^480^

Lu et al. developed an interesting strategy to increase the selectivity of glycan enrichment using two types of nanomaterials.^481^ The first nanomaterials were MNPs (70 nm) modified by boronate functionalities for glycopeptide capture, and the second were poly(methyl methacrylate) beads (200 nm) for selective peptide capture. Model glycoprotein (HRP) was detected down to 220 fmol, and enrichment could be performed in 100‐fold excess of a nonglycoprotein with a maximal binding capacity for glycopeptides of 150 mg g^−1^. MALDI‐TOF‐MS revealed 90% recovery using this synergetic enrichment, and as little as 1 μL of human serum was sufficient for analysis.^481^

MNPs could also be successfully used for the prefractionation of glycoconjugates (mainly glycopeptides and glycoproteins). Although the traditional method is hydrazide chemistry‐based solid‐phase extraction, solid‐phase extraction through reductive amination by amine‐functionalized MNPs had also been developed,^482^ shortening the extraction time to 4 hr and improving the LOD by 2 orders of magnitude. Magnetic Fe_3_O_4_ NPs were functionalized by 3‐aminopropyltriethoxysilane and subsequently incubated with glycoconjugate sample, which was converted into aldehydes by sodium periodate oxidation prior to incubation. While nonspecifically adsorbed proteins could be easily washed away, glycopeptides/glycoproteins remained immobilized on the surface. Using a specific PNGase F enzyme, an excellent isolation performance and identification of glycosylation sites using nano‐LC‐MS/MS analysis were achieved.^482^

The highest binding capacity toward glycoproteins was achieved by boronate‐modified magnetic particles (265 nm) using ovalbumin as a model glycoprotein of 778^483^ or 882 mg g^−1^ using magnetic particles with size of 500 nm.^484^

**Figure 37:**
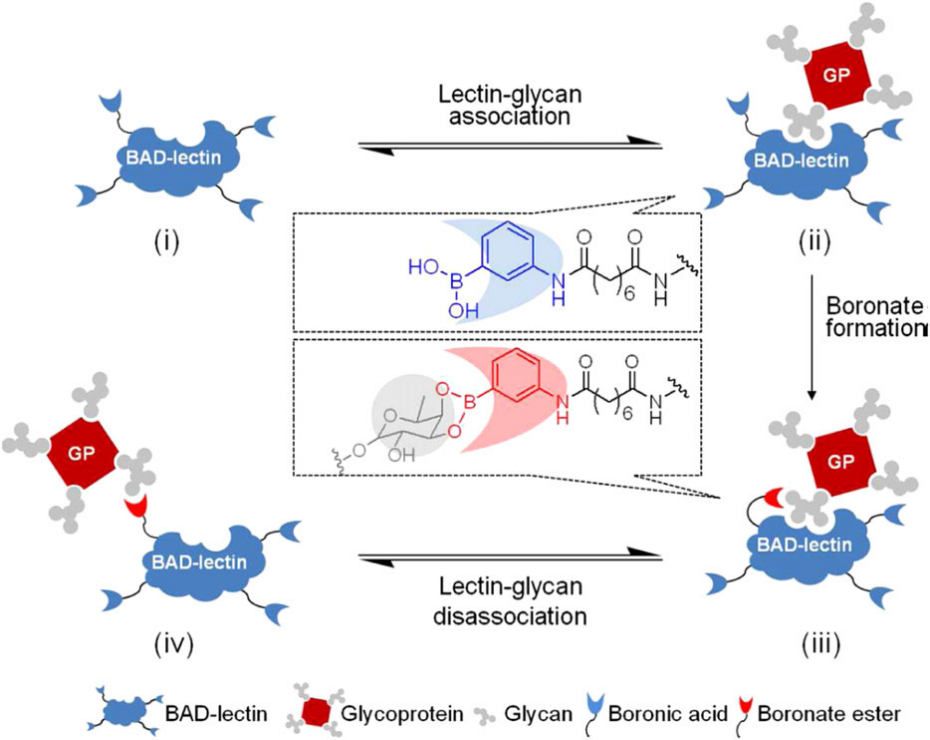
A schematic illustration of dual binding of a BAD‐lectin (BAD = boronic acid decorated) to a glycoprotein. (i) A BAD‐lectin. (ii) A glycoprotein captured by the lectin *via* noncovalent glycan‐specific recognition. (iii) A glycoprotein captured by both lectin and BA; the latter mediates the formation of a boronate ester generating a stable covalent lectin‐glycoprotein complex. (iv) A glycoprotein captured by a BA ligand alone. Reprinted with permission from 485. Copyright 2013 American Chemical Society.

An elegant method to increase the low affinity of glycan binding by three different lectins was addressed by the modification of lectins with boronate linkers—called boronic acid decorated lectins (Fig. 37)—was suggested by Lu et al.^485^ When such hybrid biomaterial was immobilized on MNPs, a 2‐ to 60‐fold increase in the detection sensitivity for glycoproteins was observed due to the increased affinity from 2.2‐fold (*Aleuria aurantia* lectin) to 5.6‐fold (Con A) for particular glycans. Glycoproteins could be detected with an LOD of 33 fmol using MALDI‐TOF‐MS. Finally, the enrichment step was utilized for the glycoproteomic analysis of a tryptic digest of HeLa cells.^485^

### G. Gold NPs (AuNPs)

**Figure 38:**
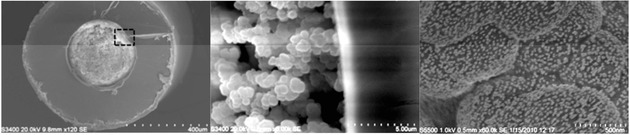
SEM images of a porous polymer monolith formed within a polypropylene pipette tip with different magnification (on left and in the middle). Field emission scanning electron microscopy images of a porous polymer monolith agglomerated with covalently attached 20 nm AuNPs with 60,000× magnification. Reproduced from 486 with permission of the Royal Society of Chemistry.

An interesting approach to simplify glycan enrichment based on the formation of a polymeric monolith within a pipette tip was presented by Alwael et al.^486^ In order to enhance the overall surface area of the monolith, 20 nm AuNPs were attached to the polymer, and the gold surface was further modified by thiols for the covalent immobilization of *Erythrina cristagalli* lectin (Fig. 38). Such a lectin‐modified monolith could selectively enrich galactosylated glycans based on the lectin´s preferential affinity with a high recovery of 95%. Finally, the device was applied in combination with reversed‐phase capillary HPLC for the analysis of *E. coli* lysate.^486^

A quite interesting approach to increase the sensitivity of glycopeptide/glycoprotein detection using LDI‐TOF (MALDI‐TOF‐MS, but without a need to use a matrix) was proposed by Liu et al.^487^ Boronate‐modified magnetic particles with a size of 412 nm were applied to capture glycopeptides from a tryptic digestion of a model protein (HRP) or intact HRP. In the subsequent step, magnetic particles with captured glycopeptides/glycoprotein were incubated with activated AuNPs (13 nm) to covalently capture glycopeptides/glycoprotein. Unbound AuNPs were removed from the system, and glycopeptides/glycoproteins were released from boronate magnetic particles by exposure to acidic pH. Finally, AuNPs with captured glycopeptides/glycoproteins were measured using MS. AuNPs containing 64,000 Au atoms each could enhance the MS analysis of glycopeptides when applied as MS tags even without the need to use a matrix since MS could detect AuNPs with an LOD of 0.03 amol and HRP with an LOD of 45 fM. Since detection is based on the analysis of the Au_2_^+^ ion rather than the analysis of glycopeptides/glycoproteins, the method can be applied not for the identification of glycoproteins but rather for obtaining information with high sensitivity when glycoproteins are present in a particular sample.^487^ Another interesting approach for the matrix‐free analysis of various low‐molecular‐weight compounds (but not glycans) was proposed by Razunguzwa et al. using an array of silicon pillars with a diameter of 150 nm, a spacing of 337 nm, and a length of 1.2 μm, which can detect analytes down to femtomole without any enrichment.^488^

Hydrazide‐functionalized ultrasmall AuNPs with a size of 1.2 nm were applied by Tran et al. for the very selective capture of periodate‐oxidized glycopeptides, when as much as 97% of all of the peptides captured from rat kidney tissue were glycopeptides.^489^ This highly selective capture of glycopeptides was possible due to the extremely high density of hydrazide on AuNPs, that is, 630 nmol mg^−1^, which is a 79‐fold higher density compared to hydrazide density on magnetic particles of 200–500 nm.^489^

### H. Silica NPs (SiNPs)

Three different strategies to deposit zwitterionic brushes with a thickness of 5 nm on the surface of SiNPs (90 nm) were compared by Huang et al. using the tryptic digest of IgG as a model glycoprotein.^490^ The results showed that the most efficient strategy for glycan enrichment was the application of NPs with the polymer grafted using reversible addition–fragmentation chain transfer with a low LOD of 10 fmol and a high recovery index of 88%. The approach was also successfully applied to analyze the tryptic digest of mouse liver with 303 unique glycosylation sites and with an enrichment efficiency of 70% using MALDI‐TOF‐MS, which was much higher compared to the other two approaches tested in the paper and other commercially available approaches.^490^

### I. Carbon NPs

#### 1. Graphene and Graphene Oxide (GO)

**Figure 39:**
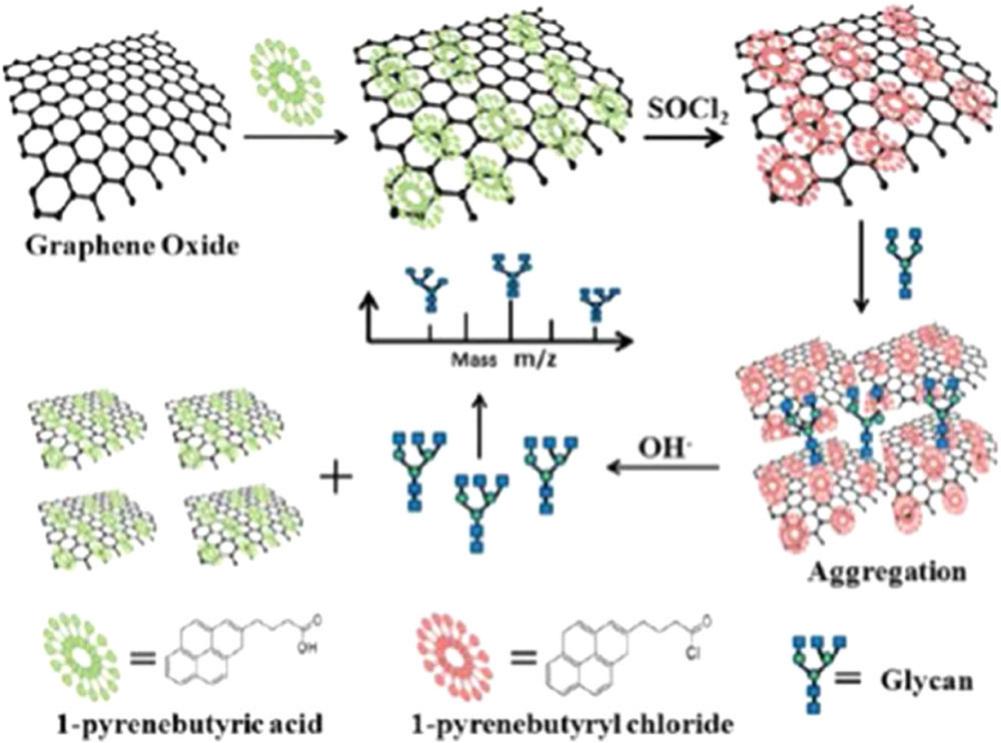
Graphene oxide modified by 1‐pyrenebutyric acid with subsequent activation of –COOH group by SOCl_2_ for effective glycan enrichment. Reprinted with permission from 491. Copyright 2013 American Chemical Society.

Graphene as a 2D crystalline material consisting of a single layer of carbon atoms with unique properties has attracted considerable attraction since its discovery in 2004.^6^ Graphene, having a high surface area, could be quite effectively applied for the enrichment of glycans. Zhang et al. recently published an approach with the application of GO noncovalently modified by pyrenebutyric acid via π–π stacking interaction between graphene and a pyrene moiety.^491^ In the subsequent step, the –COOH group was activated by SOCl_2_ to prepare pyrenebutyryl chloride‐derivatized GO, which was applied for selective glycan binding (Fig. 39). A simple visual monitoring of glycan enrichment could be conducted due to GO crosslinking by attached glycans, leading to aggregation. Finally, glycans were released from the GO surface in an acidic environment by sonication at 60°C, supporting the hydrolysis of an ester bond. The enrichment protocol allowed the detection of the main glycan from just 10 ng (i.e., 0.2 pmol) of fetuin using MALDI‐TOF‐MS, and the method was applied to analyze glycans in cancer cells. Interestingly, glycoprofiling using graphene‐based enrichment method was successfully applied even when glycoprotein was highly diluted in a protein sample not having glycans at ratio of 1:100.^491^

GO chemically modified with pyrenebutyric acid hydrazide was applied by Bai et al. for glycan enrichment.^492^ In addition to the model protein fetuin, human plasma was applied in the study with a final MALDI‐TOF‐MS analysis of the captured glycans. Glycan (maltoheptaose) recovery in a model sample spiked with BSA was 100%, but human serum spiked with maltoheptaose showed a recovery index of 73%.^492^

#### 2. Nanodiamonds

Boronate‐modified nanodiamonds could also be applied for glycan enrichment, as shown by Xu et al.^493^ In this particular case a tryptic digest of HRP as a model glycoprotein was performed, and 50‐fold enhanced MALDI‐MS spectra were obtained after glycan enrichment compared to a tryptic HRP digest without any enrichment, with HRP being detectable at an LOD of 0.5 nM in 100 μL (i.e., 0.5 fmol). The recovery index for glycopeptide analysis was 72%, and the purification efficiency for an analysis of glycopeptides in the mouse liver fraction was 69% with 24 newly identified glycosylation sites compared to databases.^493^ Loh et al. clearly showed that the modification of nanodiamonds by boronate functionalities must be performed to have a linker between the surface of nanodiamonds and boronate functional groups in order to prevent the nonspecific binding of proteins to the hydrophobic nanodiamond surface, which would negatively influence enrichment specificity.^494^

**Figure 40:**
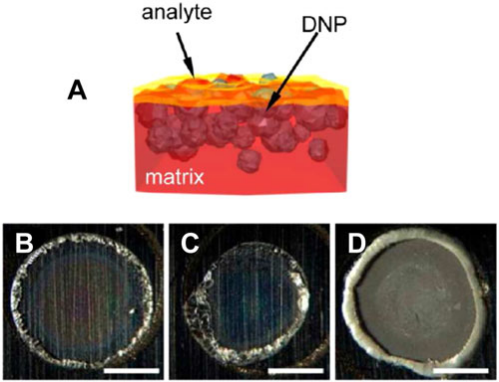
Morphology of trilayer and other samples. (A) Schematic of the configuration of matrix, DNP, and analyte in trilayer samples, (B) image of a dried‐droplet sample, (C) image of a thin‐layer sample, and (D) image of a trilayer sample containing 3 μg DNPs. The scale bars represent 1 mm. Reprinted with permission from 495. Copyright 2013 American Chemical Society.

Nanodiamond particles (DNPs) were effectively applied by Wu et al. to enhance the sensitivity of glycan analysis by MALDI‐TOF‐MS.^495^ In this case DNPs were used to transfer energy from matrix to glycans within a trilayer (i.e., by making a sandwich: matrix‐DNPs‐sample). The most important functions of DNPs were to mediate heat transfer between matrix‐ and glycan‐containing samples, avoiding direct heat exchange between these two components, and to prepare the highly homogeneous morphology of the spot on the MALDI target (Fig. 40) compared to two other methods not involving DNPs. Such particles have a low extinction coefficient not absorbing laser energy in the near‐UV region, and since DNPs are inert, they do not compete for charges with the analyte. When a model analyte dextran was analyzed by MALDI‐TOF, a 79‐ or 7‐fold increase in the detection sensitivity was achieved compared to dried‐droplet (a mixture of a matrix and a sample) or thin‐layer (layer of a sample deposited over a matrix layer) methods, respectively. The size of such particles (50–500 nm) did not influence sensitivity enhancement.^495^

#### 3. Single‐Walled CNTs

Strano´s group modified SWCNTs with derivatives of PBAs containing –COOH, ‐NO_2_, and –NH_2_ functional groups with *ortho‐*, *meta‐*, and *para‐* substitutions generating 144 distinct corona phases on the surface of SWCNTs and some of them exhibited remarkable binding affinity to certain monosaccharides, while others were not bound.^496^, ^497^ Even though the method was applied for sensing purposes (i.e., monitoring of quenching of intrinsic fluorescence of SWCNTs^496^, ^497^), this can be applied for the enrichment of glycans.^498^

### J. Hybrid NPs

A polydopamine film was deposited by Bi et al. using self‐polymerization on an rGO‐Fe_3_O_4_‐modified surface and applied for the subsequent deposition of AuNPs.^499^ Thiolated mannose finally formed SAM on AuNPs, and such a nanocomposite was employed for glycan enrichment. HRP as a model glycoprotein could be detected by MALDI‐TOF from a tryptic digest of the protein down to a concentration of 0.1 ng uL^−1^ (i.e., 40 ng or 1 pmol).^499^

Hu *et al*. modified silica bubbles (30 um) with AuNPs (20 nm), which were further functionalized with boronate‐terminated thiol.^500^ Glycopeptides prepared by a tryptic digest from two glycoproteins (HRP and IgG) could be enriched with a binding capacity of 60 mg g^−1^, and as much as 10 ng (∼200 fmol) of protein was needed for glycoprofiling with a rather low enhancement (approximately tenfold) of MALDI‐TOF signal compared to the signal obtained without enrichment.^500^

Ju et al. applied hybrid NPs (magnetic CNTs) prepared on CNTs with *d* = 40–60 nm as a scaffold by the in situ formation of MNPs (*d* = 10–15 nm) from Fe^3+^ ions.^501^ Finally, hybrid NPs were patterned by the boronate functional group. Model glycoprotein HRP could be enriched with a sorption capacity of 346 mg g^−1^, while the sorption of nonglycoprotein HSA was quite low (52 mg g^−1^). The LOD for the detection of HRP by MALDI‐TOF‐MS using an enrichment step was 1 pmol, while with commercially available boronate‐modified agarose gel, it was not possible to detect 10 pmol of the glycoprotein, and hybrid NPs could enrich glycopeptides in the presence of nonglycosylated peptides, while the latter being in 50‐fold excess.^501^

Zou et al. applied a quite sophisticated strategy for glycan enrichment.^502^ GO with deposited MNPs were covered by a silica shell of a final thickness of 50–60 nm. In the next step, the PAMAM dendrimer was grafted to the surface with subsequent modification by AuNPs on which thiolated maltose was anchored. Such a maltose‐modified hydrophilic composite with a surface area of 57.8 m^2^ g^−1^ and with a maltose density of 2.4 μmol m^−2^ could enrich glycopeptides with an LOD of 0.5 fmol. This approach was finally applied for the analysis of as low as 50 μg of mouse liver tryptic digest.^502^ For comparison, a commercially available meta‐aminophenylboronic acid modified agarose could enrich glycopeptides with an LOD of 30 nmol.^503^

Yang et al. prepared a nanocomposite with graphene as a support to accommodate MNPs (100 nm) and a phenolic‐formaldehyde resin (condensation of formaldehyde and hydroquinone) with a final modification by aminophenylboronic acid for glycopeptide enrichment.^504^ The graphene‐based composite with a thickness of 10 nm and a surface area of 76.3 m^2^ g^−1^ could detect glycopeptides down to 1 fmol using MALDI‐TOF‐MS, even when the concentration of peptides was in excess of 100:1 compared to glycopeptides. The composite was applied to analyze human serum, when as little as 1 μL of a sample was sufficient.^504^

### K. Other Interesting Approaches

Herein are described interesting strategies of effective glycan enrichment not necessarily based on the application of NPs. The approach developed by Jiao et al. is based on the use of hydrazinonicotinic acid as a matrix for the MALDI‐TOF‐MS analysis of glycans, where the matrix, besides adsorbing laser energy, also contains a hydrazine moiety for selective interaction with glycans.^505^ Thus, no glycan enrichment is needed since laser energy is mainly adsorbed by glycans even in the presence of proteins/peptides. The detection limit for a glycan is down to 1 amol, which is 5 orders of magnitude lower amount compared to 2,5‐dihydroxybenzoic acid used as a matrix. The other advantages of using this novel matrix are the higher homogeneity of glycan spots and better salt tolerance compared to the traditional matrix. The approach was finally applied for the analysis of a human serum.^505^

The online enzyme digestion of glycoproteins within a microbore hollow fiber reactor (ID = 200 um) was suggested by Kim et al. using trypsin to digest glycoproteins into peptides and glycopeptides within 30 min, which were then isolated from peptides using lectins.^506^ Finally, glycans were released from glycopeptides by the application of PNGase F, and the method was applied for the analysis of human urinary samples (PC patients).^506^ An improved assay protocol from the same group was applied for the analysis of sera from patients with liver cancer.^507^ Time needed for tryptic digestion was comparable to that needed for the microwave‐assisted release of glycans in the presence of trypsin (10 min at 50°C).^464^

Direct selective glycan enrichment was performed by Li et al. using a hydrophobic fluorinated carbon tag with an –NH_2_ terminal group for specific coupling to the reducing end of glycans.^508^ Such modified glycans could be ionized more efficiently by one order of magnitude compared to unmodified glycans using MALDI‐TOF‐MS. Alternatively, hydrophobized glycans could be selectively isolated from a mixture with other biomolecules using fluorous solid extraction.^508^

Liu et al. developed a matrix‐free strategy for MS using a lithium‐rich metal oxide composite with a particle size of 200–300 nm applied to analyze low‐molecular‐weight analytes, including several oligosaccharides (not glycans).^509^ Ruman et al. applied AgNPs with a size of 100 nm for the matrix‐free MS of various low‐molecular‐weight analytes, but not glycans, with a remarkable sensitivity of detection down to 33 amol for ribose.^510^ Moreover, the application of AgNPs as a matrix in the MS analysis of low‐molecular‐weight molecules was recently reviewed by Sekula et al.^511^

Yang et al. developed an on‐plate glycopeptide enrichment procedure using gold‐coated silicon wafer modified by SAM with a final modification by boronate functional groups taking 24 hr to complete.^512^ This approach could effectively pre‐concentrate glycopeptides released from three model glycoproteins with an LOD of 1 fmol, which increased 93‐ to 248‐fold compared to a procedure without enrichment. The plate could, however, be reused only three times because its capability to enrich glycopeptides then decreased sharply.^512^ Lu et al. deposited 900 μm gold spots on a hydrophobic silica wafer and the gold layer was patterned by 4‐mercaptophenylboronic acid for specific glycoproteins/glycopeptides enrichment.^513^ The LOD for glycopeptides released from a model glycoprotein HRP was 230 fmol, which is 1 order of magnitude lower compared to MALDI‐TOF‐MS using a standard stainless steel plate.^513^

Magnetic particles with *d* = 220 nm were used in the study performed by Xiong et al.^514^ The particles were covered by silane terminated in the –NH_2_ group, thus forming a positively charged surface. A layer‐by‐layer method was then applied to cover modified particles by the alternate deposition of negatively charged HA and positively charged chitosan. The best glycan enrichment strategy was obtained with particles having ten layers of both polysaccharides. Glycan enrichment with a binding capacity of 200 mg g^−1^ was achieved by hydrophilic interaction with glycans and polysaccharide‐modified MNPs. Three glycopeptides from a tryptic digest of IgG could be detected from just 0.2 fmol of IgG with a recovery as high as 69%. The most abundant peak in MALDI‐TOF‐MS was enhanced 111‐fold compared to the procedure without any glycan enrichment. Finally, the approach was applied for analysis of the glycoproteome from just 20 μg of a mouse liver protein sample with the identification of 605 unique *N*‐glycosylation sites in 616 distinct glycopeptides.^514^

Another approach using particles larger than 100 nm described by Ma et al. is worth mentioning.^515^ This ligand‐free approach is based on the application of hybrid particles having a magnetic core with 22 nm AgNPs deposited on the surface. Selective glycan enrichment by such hybrid particles was achieved by the reversible interaction between glycans and the Ag surface, and glycopeptides could be effectively separated from a mixture having a molar ratio of glycopeptides to nonglycopeptides of 1:100 within 1 min. Finally, the method was applied for MALDI‐TOF‐MS glycan analysis from just 1 μL of rat serum.^515^

A homogeneous system for glycan enrichment based on the pH‐responsive polymer poly‐(acrylic acid‐co‐methyl acrylate) was proven by Bai et al. to be much more effective (96.2% of glycoproteins captured within 1 hr) compared to a solid‐phase glycan enrichment (90% captured in 8 hr).^516^ The polymer with a hydrodynamic size of 30 nm was soluble at pH 6.0 but became insoluble at pH 2.0. When the hydrazide‐modified polymer was incubated with oxidized glycopeptides/glycoproteins, their separation could be performed by switching the pH to 2.0 with subsequent centrifugation. From a mixture of glycoproteins with proteins at a ratio of 1:100, low levels of glycoproteins (1 fmol) can be effectively captured. Using polymer‐based glycan enrichment MALDI‐TOF‐MS, the signal intensity increased 29‐fold and the S/N ratio increased 325‐fold. The glycan enrichment strategy was finally applied for the analysis of mouse brain samples.^516^

Zhang et al. applied a hydrophilic support‐based amino‐functionalized metal‐organic framework containing 25 nm NPs with a specific surface are of 2187 m^2^ g^−1^ for glycopeptide enrichment.^517^ A tryptic digest of IgG as a model glycoprotein could be detected after enrichment using MALDI‐TOF‐MS with an LOD of 20 fmol, and an analysis of human serum was performed from a volume as small as 10 μL.^517^

## 8. ON‐PLATE ANALYSIS OF GLYCOSYLTRANSFERASE ACTIVITIES

The identification of novel glycosyltransferases is important for the effective production of glycans since enzymatic production does not require tedious protection/deprotection steps, and even complex glycans can be effectively produced either enzymatically or by using a chemoenzymatic approach.^151^ Thus, new ways for effective and label‐free screening approaches are vital in this field. One of the most promising approaches besides offering the label‐free characterization of enzymatic activities also providing a high‐throughput analysis is to perform enzymatic reactions directly on the MALDI targets as pioneered by Mrksich´s group^518^, ^519^ and later extended also by Flitsch´s^520^, ^521^ and Reichardt´s^522^ groups. The most promising material for such targets is gold because SAMs can form on gold through a modification of thiols. When two thiols are mixed during the formation of SAM, a mixed SAM can be produced with an adjusted density of functional groups on the surface.^151^, ^523^ Mrksich´s group applied this strategy to screen 57,120 reactions (14,280 combinations of enzymes, immobilized acceptor substrates, and donor substrates in four different buffer systems) on a plate having 384 spots with 24 oligosaccharide acceptors.^524^ In the assays, 85 glycosyltransferases (including 76 bacterial enzymes that had not been previously characterized) in their crude form produced using an in vitro expression system were tested. The results using SAMs for MALDI‐MS showed 44 new glycosylation products, the enzymatic activities of four previously uncharacterized enzymes and the unknown donor preference for two known galactosyltransferases. These assays are quick, with the screening of 24 disaccharides taking 4 hr. Although various reaction conditions were tested (pH, type of a buffer, concentration of donors, time of analysis, presence of various divalent metal ions), the density of functional thiol (thiolated glycan, maleimide terminated thiols or azide terminated thiols) within a mixed SAM did not vary, with a ratio between functional and diluting thiols (OEG thiol) fixed at 1:4. The only drawback of this approach was a need to use NMR to identify the type of glycosidic linkage formed.^524^

**Figure 41:**
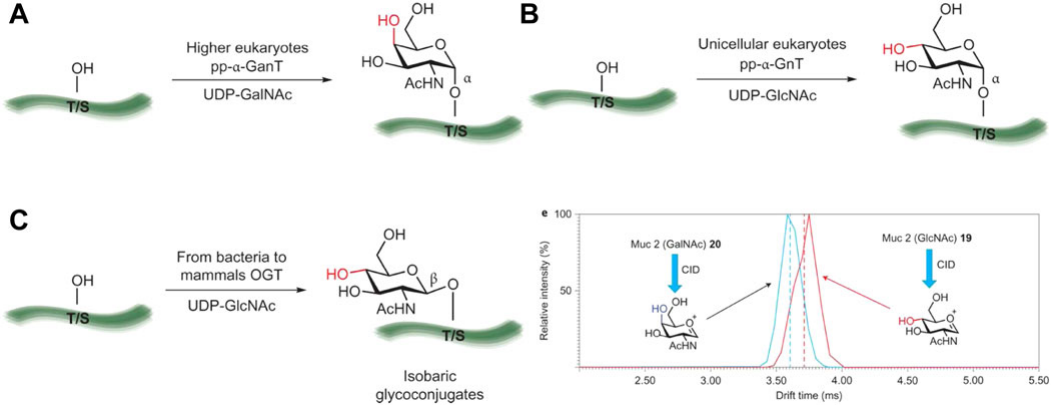
Examples of common epimeric glycoconjugates and families of enzymes involved in their biosynthesis. (a) The pp‐α‐GnT family of enzymes mediate the transfer of an α ‐linked GalNAc residue to the hydroxyl groups of serine and threonine residues in proteins. This modification is common in higher eukaryotes (including humans) and represents the first step in the biosynthesis of mucin‐type *O*‐glycans. (b) In unicellular eukaryotes, mucin‐type *O*‐glycans have been found initiated with an α‐linked GlcNAc residue generated by a pp‐a‐GnT family of enzymes. (c) β‐linked GlcNAc is a common post‐translational modification (PTM) observed in many organisms and is especially prevalent among multicellular eukaryotes. The attachment of this residue is mediated by *O*‐GlcNAc transferase (OGT) β‐linked GlcNAc is also transferred to the notch epidermal growth factor repeats by extracellular OGT. The differentiation of epimeric glycopeptides by mass spectrometry has not been reported previously, (d) Travelling wave ion mobility spectrometry mass spectrometry (TWIMS) arrival time distribution showing the discrimination of epimeric glycopeptides 19 and 20 and the distinction of HexNAc oxonium ions generated following collision induced dissociation (CID). CID of glycopeptides 19 and 20 before ion mobility separation results in the formation of epimeric oxonium ions that are distinguishable by TWIMS‐MS. Vertical dashed lines represent drift time as identified by full width half height. Reprinted by permission from Macmillan Publishers Ltd: Nature Chemistry,^525^ copyright 2014.

This drawback was recently addressed by Both et al. using travelling wave ion mobility spectrometry MS to identify diastereomeric glycoconjugates (Fig. 41) with several asymmetric centers differing only in their configuration at a single position.^525^ Moreover, GlcNAc and GalNAc oxonium ions generated by collision‐induced dissociation could be identified by ion mobility with an application for screening native glycopeptides built from different epimeric carbohydrates.^525^ The most recent study from Seeberger´s group showed that IM‐MS can unambiguously identify carbohydrate stereoisomers and linkage‐isomers even in a mixture and that the minor isomer could be detected in 1000‐fold excess of the other isomer.^526^ Kolarich´s group recently focused on the optimization of collision‐induced dissociation energy parameters for improved fragmentation of both glycans and peptides.^527^

A very interesting approach for the measurement of enzyme activities using a nanostructured MALDI surface when the matrix is not needed for laser desorption/ionization was proposed by Northen et al.^528^ The nanostructured surface was prepared as described earlier by the same group with 10 nm nanostructured pores.^529^ The enzyme substrate for glycan processing enzymes (i.e., glycosyltransferase or glycosidase) with a fluorous tag was attached to a nanohole covered by fluorous‐containing silane molecules via a soft immobilization process allowing the efficient desorption/ionization by a laser.^528^ Moreover, the substrate contained easily ionizable arginine and a five‐carbon linker to avoid steric hindrance for the binding. Such an interface offered an extremely high S/N ratio of 20 for 200 amol of a substrate, and the method could detect 500 fg of the enzyme (∼5 amol). The method was applied to detect β‐1,4‐galactosidase activity in the lysate of *E. coli* and thermophilic microbial community and to analyze enzymatic inhibitors, as well.^528^ Recently, such an approach was extended by the same group using oxime derivatization for the rapid kinetic characterization of glycosyl hydrolases from *Clostridium thermocellum*, with a possibility to identify novel hydrolases applicable for the effective production of biofuels from plant biomass.^530^

**Figure 42:**
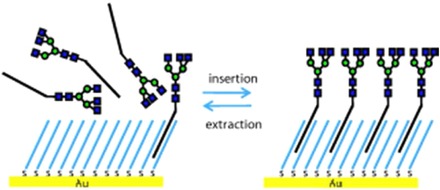
Formation of glycan‐functionalized surfaces for MALDI‐TOF‐MS analysis. Reproduced with permission from 522 by John Wiley & Sons.

Reichert´s group then built upon Wong´s approach using a hydrophobic SAM deposited on a gold surface, which was then applied for the reversible insertion/extraction of glycans with a lipid tag (Fig. 42).^522^ Such an approach was successfully applied for the analysis of activities of glycosyltransferases and glycosidases using MALDI‐TOF‐MS detection. Although the action of glycosyltransferases could be detected after 10 min of incubation, 60 hr was needed for the full conversion. For the analysis of the activity of glycosidases, 1.5 hr of incubation resulted in complete hydrolysis. In addition, the binding of lectins to glycan‐modified surfaces was performed.^522^ Later, the same group applied a slightly modified immobilization strategy (i.e., modification of the indium tin oxide [ITO] surface by hydrophobic silane, incubation with hydrophobic hydrocarbon terminated in active ester) for the covalent immobilization of either amine‐terminated glycans or lectins.^531^ Such a surface with arrayed ligands was then utilized to (i) study the enzyme activity of eight recombinant glycosyltransferases, (ii) determine the specificity of a fucosyltransferase, (iii) profile glycoproteins bound to immobilized lectins, and (iv) identify lectins bound to immobilized glycans with an on‐chip tryptic digestion and in situ peptide sequencing. A total of 54 glycan spots could be produced in 3 min, and 30 fmol of glycan could be detected by MS.^531^ Lipid and fluorous tags, if long enough, could resist their removal upon extensive washing.^55^ This approach for glycan immobilization resembles the insertion of glycans/glycoproteins within a cell membrane, offering free movement within a hydrophobic SAM.^104^, ^532^ Reichardt´s group also proved that ITO with controlled grain size (50–100 nm), roughness (1 nm), and film thickness (20–40 nm) affected by a sputtering could be applied for surface‐assisted laser desorption/ionization MS without the need to use a matrix,^533^ and such an approach has the potential to be applied in glycan enrichment due to the hydrophilic nature of the unmodified ITO surface. Another matrix‐free approach for laser desorption/ionization MS from the same group involved the use of nanostructured weathered steel (i.e., nanostructured rust layer formed on a polished steel plate with a roughness of 0.8 μm by its incubation at 37°C with 90% humidity for 2 months) hydrophobized by octadecyl trichlorosilane for sensitive glycan detection down to 5 fmol.^534^ Six different nanomaterials (AuNPs, Pt nanosponges, MNPs, TiO_2_ NPs, Se NPs, CdTe QDs) as a matrix were recently applied by Wang et al. in surface‐assisted laser desorption/ionization MS of peptides/proteins, with some of them being detected with an LOD down to 6 fmol, but glycans (glycopeptides) were not investigated in the study.^535^

## 9. MICROENGINE/MICROROCKET‐BASED ACTIVE GLYCOPROFILING

**Figure 43:**
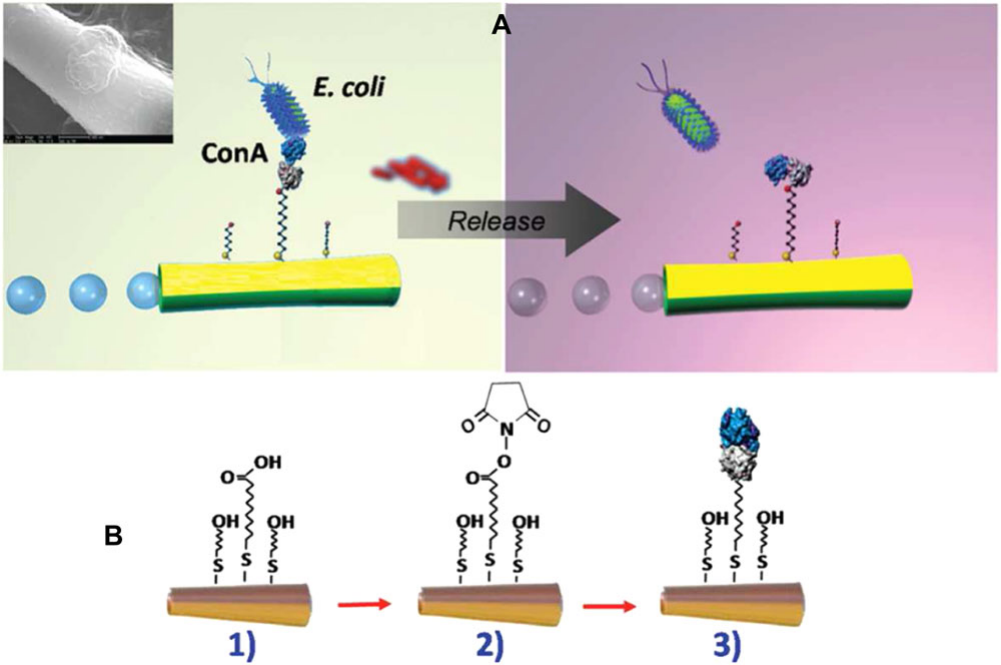
Lectin‐modified microengines for bacteria isolation. Schemes depicting (A) the selective pick‐up, transport, and release of the target bacteria by a Con A‐modified microengine, and (B) surface chemistry involved on the microengines functionalization with the lectin receptor. Upon encountering the cells, the Con A‐functionalized microengines recognize the *E. coli* cell walls by *O*‐antigen structure binding‐allowing for selective pick‐up and transport. Inset (in Scheme A, top left side): a SEM image of a portion of a Con A‐modified microengine loaded with an *E. coli* cell. Scheme A, right side: Release of the capture bacteria by navigation in a 10 mM glycine solution, pH 2.5. Scheme B: Steps involved in the microengines gold surface functionalization. (1) Self‐assembling of 11‐mercaptoundecanoic acid/6‐mercaptohexanol binary monolayer; (2) activation of the carboxylic terminal groups of the MUA to amine‐reactive esters by the EDC and NHS coupling agents; and (3) reaction of NHS ester groups with the primary amines of the Con A to yield stable amide bonds. Reprinted with permission from 536. Copyright 2012 American Chemical Society.

Nanotechnology has helped to develop novel devices that can be applied for the active glycoprofiling of various samples. Such devices are propelled by different means and, when modified by glycan binding agents (boronate functional groups or lectins), can selectively pick, transport, and release a glycan cargo (or cells) on demand. A polycarbonate membrane with 2 μm pores was applied by Joseph Wang et al.^536^ as a template for the preparation of microengines of tubular shape with a length of 6 μm and a diameter of 2 μm. The microengine was constructed using a sequential deposition of polyaniline and platinum within the pores of the membrane. Finally, the device was patterned by a thin adhesive layer of Ti (10 nm), 26 nm of Ni (a magnetic layer), and 12 nm of Au film (for the further patterning of the device). The Au surface was patterned by a mixed SAM composed of 11‐mercaptoundecanoic acid and 6‐mercaptohexanol for the covalent attachment of lectins (Con A and *Ulex Europaeus* agglutinin). The device could then be controlled magnetically and was propelled by the formation of oxygen bubbles generated by the catalytic decomposition of H_2_O_2_ (a fuel) by platinum (Fig. 43). The device selectively picked up *E. coli* cells even in the presence of yeast cells and transported them, and cargo release was triggered by a change of pH. The device could travel at a speed of 33 μm s^−1^, which is sufficient to remove bacterial cells fixed on a glass slide.^536^ In the next report from the same group, the overall concept of active glycoprofiling by a microengine/microrocket 10 μm long with ID = 800 nm and OD = 1000 nm was simplified.^537^ The device was in this case constructed from polyaminophenylboronic acid applied instead of polyaniline, which was used to selectively capture glycans. The body of the device consisted of Pt and Pt‐Ni layers within microtubules, which simplified the overall design. The device was applied for the selective capture of yeast cells via a glycan‐boronate interaction, and the cargo was released by the addition of a carbohydrate and could travel at a speed of 20 μm s^−1^ using H_2_O_2_ as a fuel.^537^

Since the application of H_2_O_2_ as a fuel can negatively impact biomolecules/cells, other options for propulsion have been sought. Ultrasound was employed by the same group to propel a device 250 nm in diameter and 1.8 μm in length (a three‐segment Au/Ni/Au device).^538^ Most likely, the combination of the small size of the device with an optimized design and ultrasound propulsion resulted in the high speed of 255 μm s^−1^ at which the device could travel while it was guided magnetically. A Con A‐modified device was applied for the selective capture of *E. coli* cells, and when antibodies were immobilized on the device, other bacterial cells could be selectively captured.^538^ The highest speed of 6 m s^−1^ for such micromotors was achieved by Joseph Wang´s group using acoustic droplet vaporization, and such microengines have strength to penetrate tissues.^539^ Furthermore, the surface roughness of the devices propelled by ultrasound can be tuned by the deposition of an Au‐Ag alloy during device fabrication with a subsequent dealloying of the silver component forming pores of 4–8 nm for the enhanced sorption or loading of biorecognition elements.^540^ The application of activated carbon for the production of highly sorptive spherical micromotors was described.^340^ In addition to bubble‐based and ultrasound propulsion, other ways to propel microengines are available, including electrical, magnetic, and self‐electrophoretic propulsion,^541^ but other propulsion principles, including light‐driven, enzyme‐catalysed reaction‐driven, and via intact motile cells, are also possible.^542^ Microengines could be made smaller for the construction of tubular devices for membrane templated synthesis with pores as small as 5 nm,^543^ and spherical nanomotors less than 100 nm in size have already been prepared.^544^ Such small micromotors have been successful in blood/serum^545^, ^546^ and even inside living cells,^542^, ^547^, ^548^ with the potential to work as miniature mixers,^549^, ^550^ and such devices could also be used for active glycoprofiling/enrichment in blood/serum or other clinically relevant samples. Moreover, in order to avoid an immune response toward artificial micromotors, red blood cells were recently turned into functional micromotors with the ability to operate in blood/serum.^551^, ^552^ The most recent study suggests that microcannons acoustically firing cargoes with an average speed of 1.05 ± 0.26 m s^−1^ could penetrate tissues with higher effectivity than microengines.^553^

## 10. CONCLUSIONS

Since quite a few different fields are covered in this review, conclusions and perspectives will be addressed for every field covered separately in the following sections.

### A. Nanoglycosensing

The section dedicated to nanomaterial‐based bioanalytical methods and biosensors described in detail some of the most commonly used nanomaterials in such applications, including metal NPs, carbon nanomaterials, polymer nanostructures, and QDs. Great attention was paid to the combination of two or more (nano)materials, called nanohybrids and nanocomposites. Although some properties of the nanomaterials are of great importance in the field of nanobiotechnology and nanobiosensing (high conductivity, large surface area, and unique shape allowing multivalent binding, optical properties, etc.),^554^, ^555^ few real applications (i.e., analysis of real samples) have been described in the current literature.^556^ Most of the presented papers dealt only with commercially available substances or model cell lines, although there is a growing number of papers trying to dynamically evaluate the number of membrane‐bound glycans on living cells for clinical diagnostics.^557^ When used in a real clinical laboratory, such cells have to be isolated from patient tissues (taken during biopsy); therefore, less invasive methods to isolate the samples are welcome. The use of human sera or even whole blood is limited, mostly because of a small level of a particular biomarker present in body fluids.^558^, ^559^, ^560^, ^561^ Using a combination of immobilized antibodies (able to specifically bind a biomarker from the real sample) together with a lectin able to “decipher” the biomarker´s glycocode might be a solution in some cases.^562^

### B. Carbohydrate‐Based Vaccines and Therapeutics

To summarize the therapeutic applications of carbohydrates integrated with nanomaterials or carbohydrates synthetized with a controlled density on various supports at the nanoscale, three main application directions can be distinguished: (i) employment of carbohydrate‐containing immunogens, especially viral and bacterial glycans responsible for cell recognition and consequent immune response, and tumor‐associated antigens^188^; (ii) development of therapeutics based on the selective blocking of cell receptors *via* competitive lectin‐carbohydrate binding^563^; and (iii) employment of carbohydrates as building blocks to develop NPs with therapeutic effects of molecules other than carbohydrate‐based moieties.

The most recent progress in the development of carbohydrate nano‐vaccines is represented by the development of a method of chemoenzymatic synthesis allowing the tailored conjugation of glycans to carrier proteins leading to the increased immunoefficiency of the prepared NPs.^188^ Another promising achievement was to conjugate tumor‐associated antigens to NPs even though non‐carbohydrate antigens seem to provide more significant results in this field.^564^, ^565^

The therapeutic effect outlined in (ii) has recently led to the development of NP‐glycan conjugates with higher affinity to specific receptors than bacterial or viral glycans. In the majority of studies, such NPs were employed as antibacterial and antiviral drugs because of their ability to selectively inhibit the first phase of infection. Recent studies have suggested that a combination of multivalent glycans and the selectivity of lectin‐glycan binding could be a step further in the development of completely new class of antibiotics.^566^ It should be noted that, regardless of studies employing metallic and carbon NPs, (bio)polymers and synthetic micelles are the most preferable carriers for the same reasons as in the case of glycan nano‐vaccines.

### C. Cell Targeting

While the nanomaterials used for delivery of carbohydrate‐based therapeutic agents (antigens or non‐immunogens) were mostly AuNPs, (bio)polymers, and dendrimers, there was much higher diversity of nanomaterials tested or developed as nanocarriers for drug delivery of drugs other than glycans, targeted by conjugated glycan moieties. Very intensive research is currently underway to explore targeting abilities, biocompatibility, and other advantages of HA, which has been used in many studies as an NP‐coating, securing selective internalization by tumor cells via their surface displayed CD44 receptors.^567^ In a typical approach, HA has been used for the decoration of gold,^568^, ^569^ carbon,^288^ or silica^310^ NPs or synthetic NPs^570^ conjugated with doxorubicin (a model cytostatic drug). Many studies have reported the inhibition of tumor growth after treatment by such NPs, but not at a significant rate. Nevertheless, efficient targeting is a crucial step in avoiding the severe adverse side effects of conventional cancer chemotherapy. In addition to glycan‐driven targeting, recent studies have also focused on the selective release of the delivered drug after internalization. Here, again, HA is an intriguing material because it could be degraded by intracellular hyaluronidases, liberating the drug from HA‐based NPs.^571^ For many nanocarriers, the final drug release was secured by a significantly lower pH in lysosomes, which the NP could enter only after surface receptor‐driven internalization.^310^, ^568^ Another recently studied way to keep the nanocarriers with their cargo intact until they reach the target is the introduction of disulfide bonds into the NP structure. This bond could be readily reduced by glutathione, which is typically present at an elevated concentration in the cytoplasm of cancer cells.^310^ Interesting results have also been achieved by the development of nanocarriers capable of passing through the blood brain barrier, for example, by the activation of a glucose transporter or by the conjugation of NPs with glycoproteins or lectins. This is important for the selective chemotherapy of brain tumors. In addition to drug delivery, carbohydrate‐targeted NPs were also promising as nonviral vectors, which has opened new possibilities in gene therapy for cancer.

In addition to chemo‐ and geno‐therapies, targeting delivery has also been recently used for so‐called photodynamic and photothermal therapies. While in the former case, photosensitizers are delivered into cells that generate cytotoxic ROS,^375^, ^571^, ^572^ the latter method relies on the delivery of NPs causing local overheating by irradiation after their internalization into selected cells.^573^ In both cases, the desired action is typically triggered by NIR irradiation, which is harmless to healthy tissues without internalized photosensitizers. Another advantage of these methods is that it is not necessary to release the cargo from the carriers; the only aim is to get into the desired cells as many nanocarrier particles as possible, while a minimal amount should be in healthy tissue, as indicated by recent progress in carbohydrate‐based targeting.

### D. Cell Imaging

Upon the replacement of the therapeutic agents discussed in the previous section with imaging probes, efficient diagnostic tools can be obtained not only for the visualization of tissue with an unusual glycopattern (most often tumors^390^ or inflammation sites^574^) but also for the very precise detection and assessment of, for example, surface cell receptors, lectin‐binding properties, and other features necessary for the further development of a glycan‐based targeted therapy. While such assessments are typically performed in vitro, NIR or UV‐VIS imaging probes can be used. Organic dyes coupled to glycan‐based nanocarriers are typically used for imaging in this range, and recently, so‐called theranostic NPs have been developed where imaging probes together with therapeutic agents are incorporated (see for example 575); thus, drug delivery can be monitored and confirmed. It should be noted that some organic therapeutic agents could also simultaneously function as imaging probes (e.g., doxorubicin or most photosensitizers), making theranostic NPs less expensive and easier to fabricate. For efficient in vivo imaging, CT and MRI have been routinely used for many years. Recent studies have focused on the targeted delivery of contrast enhancers, allowing the efficient visualization of selected tissues. While selectively distributed AuNPs have typically been used for CT imaging,^400^ iron oxide containing NPs have been tested extensively as MRI imaging probes.^576^

### E. Glycan Enrichment and Separation

It can be concluded that the application of nanomaterials or mesoporous materials with various functionalities present on the surface can significantly decrease the time needed for glycan enrichment (i.e., from a typical time of 160^577^ to 1^515^ min). Moreover, when using hybrid MNPs, the separation time can be as short as 15 sec by the simple application of an external magnet,^476^ thus rendering the use of additional equipment (centrifuge) with a much longer separation time (few minutes) unnecessary.

Nanomaterial‐based glycan enrichment could be applied for a minute amount of a starting material. Glycans can be enriched with commercially available hydrophilic beads with an LOD of 40 fmol,^466^ and the most sensitive enrichment procedure involving MNPs with thin SiO_2_ layer and zwitterion modification could detect as low as 100 amol^475^ of glycan. Thus, nanomaterials can help to enrich glycans with an LOD 3–9 orders of magnitude lower compared to commercially available supports, but some approaches can be quite sophisticated, involving five different components. The high effectivity of glycan enrichment also means that an ultralow amount of often precious samples is required, that is, 50 nL of human serum with an application of a carbonized mesoporous silica composite.^460^ Glycan enrichment can be effective in the presence of nonglycoprotein or nonglycopeptides being in excess over glycoproteins/glycopeptides with a ratio of 1000:1^467^ and also in a 1 million‐fold excess of a competing monosaccharide^479^ due to high affinity toward glycans.

Not only glycan enrichment can be made more effective using nanomaterial‐based approaches, but nanomaterial can also be applied as a support for the immobilization of trypsin or PNGase to perform an enzymatic reaction on a surface. The advantage of using immobilized enzymes is their reuse, since, for example, the stability of PNGase F covalently immobilized on GO was up to 8 weeks when stored at 4°C, and as little as 2 μg uL^−1^ of the enzyme complex was needed to analyze plasma extracts.^465^ Moreover, the reaction was completed within 2 min, while usually overnight incubation was needed with a soluble enzyme.^465^ When PNGase F was covalently immobilized on a porous silica column, the reaction was completed within 6 min.^463^ Time needed for on‐line glycan removal was even shorter compared to that needed for the microwave‐assisted release of glycans in the presence of PNGase F (30 min at 37°C).^464^ Immobilized trypsin was also applied for on‐line protein digestion,^506^ which could be used for continuous protein digestion suitable for automatic glycan‐detecting systems.

Nanomaterials can have additional beneficial effects on glycan analysis. For example, the addition of DNPs to the MALDI target in a layer‐by‐layer approach improved spot morphology and enhanced the sensitivity of MALDI‐TOF‐MS detection 79‐ or 7‐fold compared to the dried‐droplet (a mixture of matrix and sample) or thin‐layer (layer of sample deposited over a matrix layer) method, respectively.^495^ The main principle behind this is the mediation of heat transfer between matrix and glycans by controlled architecture.^495^ Considering the efficiency of energy absorption from the laser, the most effective way was the use of hydrazinonicotinic acid as both the matrix and glycan‐binding agent.^505^ Thus, glycan enrichment directly on a plate was applied with an impressive LOD for glycan MALDI‐TOF‐MS analysis down to 1 amol, which is 5 orders of magnitude lower than that of 2,5‐dihydroxybenzoic acid, which is traditionally applied as the matrix.^505^

MIP with a controlled thickness over imprinted analyte at the nanometer scale could offer a quite high imprinting ratio (i.e., ratio of glycoprotein attached to imprinted vs. nonimprinted NPs) of 22^472^ and a quite high selectivity for its analyte of 30 over other glycoproteins,^470^ which is useful for selecting a particular glycoprotein even from a quite complex sample.

## 11. PERSPECTIVES

### A. Nanoglycosensing

Moreover, the next step for researchers dealing with lectin–glycan interactions should be to overcome lectin “promiscuity” by tuning their specificity (e.g., using recombinant technology to generate mutant proteins^578^) or to prepare more specific aptamers (DNA or peptide) with better binding properties for recognising glycans, which could be immobilized on surfaces with controlled orientation/precision because of their smaller size.^579^, ^580^ Molecular imprinting technology for the differentiation of two glycan moieties with the same composition but different glycosidic bond(s), as in the case of 3´‐ and 6´‐sialyllactose mentioned in the Section A..A, should also be investigated.^581^, ^582^ Most of the presented methods involve expensive equipment (SPR) or methods with a complicated theoretical background (EIS), which could be interpreted only by a skilled person. Cheap protocols (for instance, those using common microplates) with possible naked‐eye detection are welcome. In addition, some of the methods, even well understood, still cannot be used outside of the laboratory and thus integrated into commercially mass produced point‐of‐care devices. Such barriers to commercialization are significant problems in the case of impedimetric biosensors because of the lower stability and reliability of these biosensors compared to other types.^583^ Sensor regeneration and flow systems are an issue to focus on, even though they are not as important as the need for detection in an array format for highly parallel analysis. An array assay format in combination with more sensitive platforms compared to generally employed fluorescent techniques (ideally, electrochemical methods compatible with miniaturization and nanomaterial‐based modifications) still needs to be developed for a wider use.

### B. Carbohydrate‐Based Vaccines and Therapeutics

From a different perspective, there are barely any studies exploring the use of nanomaterials other than AuNPs and synthetic micelles or biopolymer scaffolds. In this regard, reported iron oxide NPs conjugated with glycans^226^ can be considered a way for the future preparation of immunogenic nanoconjugates based on nanomaterials being cheaper than AuNPs. Natural polysaccharides are also known to increase the stability of vaccine NPs. More importantly, the immunogenicity of these materials and their use as excellent adjuvants has to be tested. From this point of view, chitosan and its derivatives are polysaccharides that are frequently applied for such purposes.^202^, ^584^

Also very important is the recent progress in the controlled synthesis^585^ and evaluation of iminosugars^586^ conjugated with NPs, that is, pharmacological chaperones responsible for partial functional restoration of glycosidases, whose misfolding results in lysosomal storage diseases. It seems that in this particular field, the synthesis of dendrimers or scaffolded iminosugar carriers requires a meticulously defined conformal structure, conjugate size, valency, and spacers between a carrier and an iminosugar moiety.^586^

### C. Cell Targeting

The targeting, delivery, and release mechanisms are very similar to those employed in targeted drug delivery. Importantly, even though genotherapy itself may not provide the highest efficiency, it is possible to increase sensitivity of tumor cells to another treatment (e.g., to cytostatics) by the application of specific short nucleotides, thus allowing such a combined therapy to become more efficient.^351^ Furthermore, a reliable and specific gene delivery would probably be necessary for further progress in personalized medicine.

Finally, it should be noted that the most rapid tumor growth inhibition has been observed typically when a combination of methods was used; for example, chemophototherapy or chemothermal therapy.^288^ In the further development of carbohydrate‐based nano‐therapeutics, such synergy of effects would probably be the most promising path to follow.

### D. Cell Imaging

Considering that AuNPs are excellent thermodynamic therapy agents, we see another way for the development of multifunctional NPs without any additional fabrication steps or additional components used. From this perspective, graphene and its derivatives were found to have even more promising attributes—they could be used as drug (and glycan‐based targeting moieties) carriers, thermodynamic therapy agents, and fluorophores.^288^, ^587^ Furthermore, graphene QDs, that is, nanometer‐sized graphene sheets, can replace conventional semiconductors and usually cytotoxic (unless they are made biocompatible, for example, by carbohydrate coating) QDs.^588^ It is anticipated that graphene derivatives will be investigated more intensively in years to come to obtain cheap, easy‐to‐fabricate and efficient theranostic NPs.

### E. Glycan Enrichment and Separation

Finally, we can say that even though nanomaterials or nanoporous materials exhibit remarkable efficiency in glycan profiling due to their robustness, the reproducibility of the glycan enrichment process together with their mechanical/chemical stability has to be more rigorously compared, ideally in studies involving multiple institutions, as recently performed for the glycoprofiling of *N*‐glycans of IgG^589^, ^590^, ^591^ or *O*‐ and *N*‐glycans from cultured cell lines,^592^ to identify strong and weak attributes for each glycan enrichment technique.

The other class of affinity‐based molecules, which can be applied in the future to selectively detect glycoproteins/glycopeptides or for glycan enrichment, are DNA/RNA aptamers designed to recognize glycoproteins since their *K*_D_ for glycans can be lower compared to lectins.^459^ There is however hope for the more frequent application of modified lectins in future glycan enrichment, when lectin modified with boronate will be applied for the more sensitive binding of glycans.^485^ Moreover, various types of microengines/microrockets can also be applied for active glycan enrichment from quite complex samples.

**12. ABBREVIATIONS**
AgNPs: silver nanoparticles
AuNPs: gold nanoparticles
AuNRs: gold nanorods
BSA: bovine serum albumin
CEA: carcinoembryonic antigen
cfu: colony‐forming unit
CNTs: carbon nanotubes
Con A: Concanavalin A
CT: computational tomography
CuAAC: Cu^I^ catalyzed Huisgen azide‐alkyne cycloaddition
CVD: chemical vapor deposition
DC‐SIGN: Dendritic Cell‐Specific Intercellular adhesion molecule‐3‐Grabbing Non‐integrin
DNPs: nanodiamond particles
DPV: differential pulse voltammetry
ECL: electrochemiluminiscence
EIS: electrochemical impedance spectroscopy
ESI: electrospray ionization
FimH: type 1 fimbrial adhesin
FITC: fluorescein isothiocyanate
GCE: glassy carbon electrode
GlcNAc: *N*‐acetylglucosamine
GO: graphene oxide
GOx: glucose oxidase
HA: hyaluronic acid
HRP: horseradish peroxidase
IgG: immunoglobulin G
ITO: indium tin oxide
LacNAc: *N*‐acetyl‐d‐lactosamine
Le^a^: Lewis a antigen
Le^x^: Lewis x antigen
Le^y^: Lewis y antigen
LOD: limit of detection
LPS: lipopolysaccharide
LSPR: localized surface plasmon resonance
MALDI: matrix‐assisted laser desorption/ionization
MALDI‐TOF‐MS: matrix‐assisted laser desorption/ionization time‐of‐flight mass spectrometry
MIP: molecularly imprinted polymers
MNPs: magnetic nanoparticles
MS: mass spectrometry
MUC1: immunogenic synthetic mucin
MWCNTs: multiwalled carbon nanotubes
NIR: near infrared
NMR: nuclear magnetic resonance
NMRi: nuclear magnetic resonance imaging
NPs: nanoparticles
OEG: oligoethylene glycol
PAMAM: poly(amidoamine)
PBA: phenylboronic acid
PC: prostate cancer
PdNPs: palladium nanoparticles
PEG: polyethyleneglycol
PEI: poly(ethylenimine)
PNGase A: peptide‐*N*‐glycosidase A
PNGase F: peptide‐*N*‐glycosidase F
PSA: prostate‐specific antigen
PtNPs: platinum nanoparticles
QCM: quartz crystal microbalance
QDs: quantum dots
rHDL: reconstituted high‐density lipoprotein
ROS: reactive oxygen species
SAMs: self‐assembled monolayers
SERS: surface enhanced Raman scattering
SiNPs: silica NPs
siRNA: small interfering RNA
sLe^a^: sialyl Lewis a antigen
sLe^x^: sialyl Lewis x antigen
SNA: *Sambucus nigra* agglutinin
SPR: surface plasmon resonance
SR‐BI: Scavenger Receptor class B member 1
SWV: square‐wave voltammetry
RCA_120_: *Ricinus communis* agglutinin with *M*_w_ = 120 kDa
rGO: reduced graphene oxide
SWCNTs: single‐walled carbon nanotubes
TEOS: tetraethylorthosilicate
TF_ag_: Thomas Friedrich antigen
WGA: wheat germ agglutinin
